# Clinical efficacy, safety, and cost of nine Chinese patent medicines combined with ACEI/ARB in the treatment of early diabetic kidney disease: A network meta-analysis

**DOI:** 10.3389/fphar.2022.939488

**Published:** 2022-08-22

**Authors:** Jiarong Liu, Xuehan Zhang, Gaosi Xu

**Affiliations:** Department of Nephrology, The Second Affiliated Hospital of Nanchang University, Nanchang, China

**Keywords:** Chinese patent medicines, network meta-analysis, diabetic kidney disease, adverse effects, therapies

## Abstract

**Objectives:** To evaluate and compare the efficacy, safety, and cost of nine Chinese patent medicines (CPMs) combined with angiotensin-converting enzyme inhibitor (ACEI) or angiotensin receptor blocker (ARB) in treating early diabetic kidney disease (DKD).

**Design:** Systematic review and network meta-analysis.

**Data sources:** PubMed, Embase, Cochrane Library, Web of Science, clinicaltrials.gov, SinoMed, Chinese Biomedicine, China National Knowledge Infrastructure, WanFang, and Chongqing VIP Information databases were comprehensively searched from the beginning to February 2022.

**Review Methods:** Randomized controlled trials (RCTs) including Bailing capsule (BLC); Jinshuibao capsule (JSB); Huangkui capsule (HKC); Compound Xueshuantong capsule (CXC); uremic clearance granule (UCG); Shenyan Kangfu tablet (SYKFT); tripterygium glycosides (TG); Keluoxin capsule (KLX), and Shenshuaining tablet (SSNT) combined with ACEI/ARB for patients with early DKD were reviewed.

**Data Synthesis:** Two reviewers independently screened articles, extracted data, and assessed the risk of bias. Risk ratios (RRs) and mean difference (MD) were reckoned to assess dichotomous variable quantities and continuous variable quantities, respectively. Using the surface under the cumulative ranking curve (SUCRA), we then ranked each therapeutic regime.

**Results:** Ultimately, 160 RCTs involving 13,365 patients and nine CPMs were included. UCG showed significantly higher probabilities on urinary albumin excretion rate (UAER) when compared with ACEI/ARB group, with MD of −47 (95%CI) (−57, −37) and SUCRA 98.0%. The CXC group achieved a remarkable improvement in overall response rate (ORR) compared with ACEI/ARB (RR, 1.3, 95%CI (1.2, 1.5)) with SUCRA 91.9%. SSNT could be significantly superior to ACEI/ARB group in terms of serum creatinine (Scr) (−19 (−26, −12), SUCRA 99.3%) and adverse effects (AEs) (0.46 (0.17, 1.1), SUCRA 82.9%). BLC showed the greatest effectiveness on 24 h urinary total protein (24 h UTP) (−170 (−260, −83), SUCRA 78.5%) and triglyceride (Trig) (−0.89 (−1.2, −0.53), SUCRA 97.0%). From the cost-effectiveness analysis of CPMs in China, the cost of TG, SYKFT and CXC was 108, 600, and 648 RMB, respectively, per 3 months and were ranked in the top three.

**Conclusion:** UCG and CXC might be the optimum selection for improving UAER and ORR, and SSNT could be significantly superior to ACEI/ARB group in terms of Scr and AEs. BLC shows the best curative effect on 24 h UTP and Trig. TG shows the highest cost-effectiveness among the nine CPMs.

**Systematic Review Registration:** https://www.crd.york.ac.uk/prospero/, identifier CRD42022314843.

## 1 Introduction

Diabetes mellitus (DM) is thought to lead to microvascular damage most frequently to kidneys (Thomas et al., 2015). There has been a quadrupling of people worldwide with diabetes mellitus in the past 30 years (Zheng et al., 2018). Diabetic kidney disease (DKD), the leading cause of end-stage renal disease (ESRD) in the world, affects about 40% of diabetes mellitus patients. In 1987, according to the pathophysiological characteristics and evolution process of DKD, Mogensen, a Danish scholar, divided DKD into five stages. In clinical practice, it is difficult to detect DKD stage I or II, so once microalbuminuria is revealed, it is considered DKD stage III. Consequently, DKD stage III is known as early DKD. DKD can be reversed, and the progression of the disease can be slowed if it is detected and treated at an early stage. Otherwise, renal function will continue to deteriorate, even leading to renal failure once DKD progresses to stage IV or V. Therefore, a timely diagnosis and early intervention are crucial to preventing DKD (Mogensen et al., 2019). It is widely accepted that urinary albumin excretion rate (UAER) can be used for diagnosis and clinical grading of DKD (Chen et al., 2017). The effective treatments of DKD mainly include general treatments, such as healthy lifestyle and dietary habit, and pharmaceutical treatments, such as angiotensin-converting enzyme inhibitors (ACEIs) and angiotensin receptor blockers (ARBs). In comparison with ACEI/ARB alone, several studies have suggested that traditional Chinese medicine (TCM) in conjunction with ACEI/ARB has better efficacy with fewer side effects for patients with DKD (Xiao et al., 2013; Liu et al., 2014; Li and Xu, 2020).

The Chinese traditional medicine system has played an essential role in China’s health care for thousands of years. A variety of TCM extracts or combination preparations has shown remarkable curative effects on various diseases and also has been verified to have definite kidney-protection effects (Wang et al., 2019a). Chinese patent medicines (CPMs), as an important part of TCM, can be directly used for disease prevention and treatment after processing with a certain formula. CPMs, including Bailing capsule (BLC); Jinshuibao capsule (JSB); Huangkui capsule (HKC); Compound Xueshuantong capsule (CXC); uremic clearance granule (UCG); Shenyan Kangfu tablet (SYKFT); tripterygium glycosides (TG); Keluoxin capsule (KLX), and Shenshuaining tablet (SSNT), have been extensively used and have been confirmed to have a definite beneficial effect in treating DKD (Luo et al., 2015). There have been several related reviews and meta-analyses published in recent years, and they have evaluated the efficacy and safety of CPM alone and in combination with ACEI/ARB (Li and Xu, 2020; Sheng et al., 2020; Wu et al., 2020). However, these studies have some limitations for a variety of reasons, such as only involving a single CPM or including participants with DKD at all stages. Hence, which kind of CPM has a better curative effect in treating early DKD remains unknown, posing a great challenge for clinicians in choosing appropriate CPMs for DKD patients.

In contrast with traditional meta-analyses, network meta-analysis (NMA) offers greater value, which can rank multiple interventions simultaneously by combining direct and indirect comparison results (Salanti, 2012). Therefore, a NMA to rank the effectiveness, safety, and cost of the nine CPMs for patients with early DKD was performed. Clinical decision makers can use the data we provide to determine what treatment is most appropriate for patients with early DKD based on evidence-based medicine. The China State Food and Drug Administration has approved all nine CPMs.

## 2 Materials and methods

We followed the Guidelines for Preferred Reporting Items for Systematic Review and Meta-analysis (PRISMA) in conducting our study (Page et al., 2021). As an additional material (see Supplementary Material S1), the PRISMA checklist has been provided. Additionally, our study has been registered with the International Prospective Register of Systematic Reviews (PROSPERO: CRD42022314843).

### 2.1 Eligibility criteria

#### 2.1.1 Types of studies

We included RCTs in which they compared the efficacy of nine CPMs in combination with ACEI/ARB against ACEI/ARB alone in treating early DKD. Although there was no restriction on the language of RCTs, RCTs were required to include primary or secondary outcome indicators (see Supplementary Table S1 for specific selection criteria).

#### 2.1.2 Types of participants

Patients who were diagnosed with diabetes mellitus and met the diagnostic criteria of early diabetic kidney disease were included. The UAER of 20–200 g/min or the urinary albumin-creatinine ratio of 30–300 mg/24 h is considered early DKD as defined by internationally recognized stage criteria according to Mogensen (1987). Factors such as the patient’s age, gender, region, race, or course of the disease did not matter.

#### 2.1.3 Types of interventions

As part of the study, the treatment group was given one of nine CPMs (BLC, HKC, JSB, UGG, TG, CXC, SYKFT, KLX, and SSNT) in combination with an ACEI or ARB. Additionally, the control group was given an ACEI/ARB alone or an ACEI/ARB combined with any of eight other CPMs. Both groups continued to maintain general treatment, such as the management of blood pressure, blood glucose, and blood lipid. The courses of treatment could not be less than 2 weeks. There were no restrictions on the dosage of nine CPMs and ACEI/ARB.

#### 2.1.4 Types of outcomes

The primary outcomes include urinary albumin excretion rate (UAER), overall response rate (ORR), serum creatinine (Scr) and 24 h urinary total protein (24 h UTP). ORR was defined as either a complete or partial response rate (CRR or PRR). CRR was defined as the clinical symptoms disappearing or the urinary albumin excretion rate returning to normal or decreasing by more than 50%. PRR was defined as the clinical symptoms relieved or the urinary albumin excretion rate decreasing, but the decrease range was not obvious. Secondary outcomes include total cholesterol (TC), triglyceride (Trig), and glycosylated hemoglobin, type A1c (HbA1c). Safety outcome was adverse effects (AEs).

### 2.2 Excluded criteria

Publications that met the following criteria were excluded: 1) diagnostic criteria for early DKD were not met by the patients; 2) early DKD was mentioned in the titles or abstracts of articles, but the inclusion criteria were not described specifically in the body text; 3) patients took other various traditional Chinese medicines besides the nine CPMs mentioned in our study; 4) the courses of treatment were less than 2 weeks; and/or 5) the main outcome measures of RCTs were not included in our defined primary outcomes or secondary outcomes.

### 2.3 Search strategy

We extensively searched PubMed, EMbase, Cochrane Library, Web of Science, clinicaltrials.gov, SinoMed, Chinese Biomedicine, China National Knowledge Infrastructure, WanFang, and Chongqing VIP Information databases from the beginning to February 2022 for RCTs comparing the effectiveness of different CPMs and ACEI/ARB for patients with early DKD. There were no limitations on the language, publication year, or blinding methods. The retrieval strategies were implemented using a combination of MeSH terms and free words. The predefined key searching strategy was as follows [(Diabetic Kidney Disease) OR (Diabetic Nephropathy) OR (Diabetic Glomerulosclerosis) OR (Glomerulosclerosis, Diabetic) OR (Diabetic Nephropath*) OR (Kidney Disease*, Diabetic) OR (Nephropath*, Diabetic) OR (Diabetic Glomerulosclerosis) OR (Early) OR (Stage three) OR (Stage III)] AND [(Bailing Capsule) OR (Huangkui Capsule) OR (Jinshuibao) OR (Uremic Clearance*) OR (Tripterygium glycosides) OR (Xueshuantong Capsule) OR (Shenyan Kangfu Tablet) OR (Keluoxin Capsule) OR (Shenshuaining Tablet)]. In addition, we manually screened the literature list to prevent the omission of appropriate literature as well.

### 2.4 Literature screening and data extraction

We used endnote software to manage the retrieved literature. We obtained the articles that met the inclusion criteria for evaluating and obtaining the data after screening their titles and abstracts. Two reviewers (JRL and XHZ) extracted data independently using Microsoft Excel. Differences in opinions in the process of data extraction were solved by a third reviewer (GSX). The contents of data extraction included basic characteristics of the included literature (country, publication year, and first author), subjects for study information (mean age, sex ratio, sample size, and basal blood pressure), interventions (different CPMs, ACEI/ARB, course of treatment, and period of follow-up), and reported outcomes (Scr, UAER, ORR, 24 h UTP, HbA1c, TC, Trig, and AEs). For information that cannot be obtained directly, we tried to contact the author by email.

### 2.5 Risk of bias assessment

Two reviewers (JRL and XHZ) assessed the risk of bias for all included studies independently according to the Cochrane Risk of Bias tool [Cochrane Handbook for Systematic Reviews of Interventions, version 5.4.0] (Higgins et al., 2011). Each domain can be judged as high risk, low risk, or unclear risk for included studies. A third reviewer (GSX) adjudicated any disagreements.

### 2.6 Statistical analysis

The effect sizes of continuous and dichotomous outcomes were described by calculating mean differences (MD) and risk ratios (RR) with 95% confidence intervals (95% CI). A difference between two comparison groups is considered to be statistically significant if zero does not appear in the 95% confidence interval for MD or one does not appear in the 95% confidence interval for RR. Before carrying out our statistical analysis, the STATA 14.0 package (“mvmeta” and “network”) was installed to produce the network diagram and determine the size of SUCRA as well as to create funnel plots for assessing publication bias and R 3.5.1 package (“ggplot2” and “gemtc” packages) was installed to draw forest plots, make league tables, and perform heterogeneity analysis. A total of 1,000,000 simulations were generated for each of the two sets of initial values and the first 50,000 simulations were discarded as the burn-in period. Next, Brooks–Gelman–Rubin diagnostics and traces were used to examine the convergence and density diagrams. SUCRA measures the likelihood that a therapeutic schedule will produce the best results as a percentage, which is infinitesimally close to one when treatment is considered the best, and infinitesimally close to zero when it is deemed the worst (Salanti et al., 2011). It is considered that the heterogeneity is not significant if *p* > 0.05 in direct and indirect comparisons. Then, we used the consistency model for the following statistical analysis. In evaluating pairwise and network heterogeneity, *I* ^*2*^ greater than 50% indicates significant heterogeneity. The heterogeneity of both primary outcomes and secondary outcomes was less than 10%, indicating low heterogeneity overall (see Supplementary Table S2 for details).

## 3 Results

### 3.1 Selection and identification of studies

**FIGURE 1:**
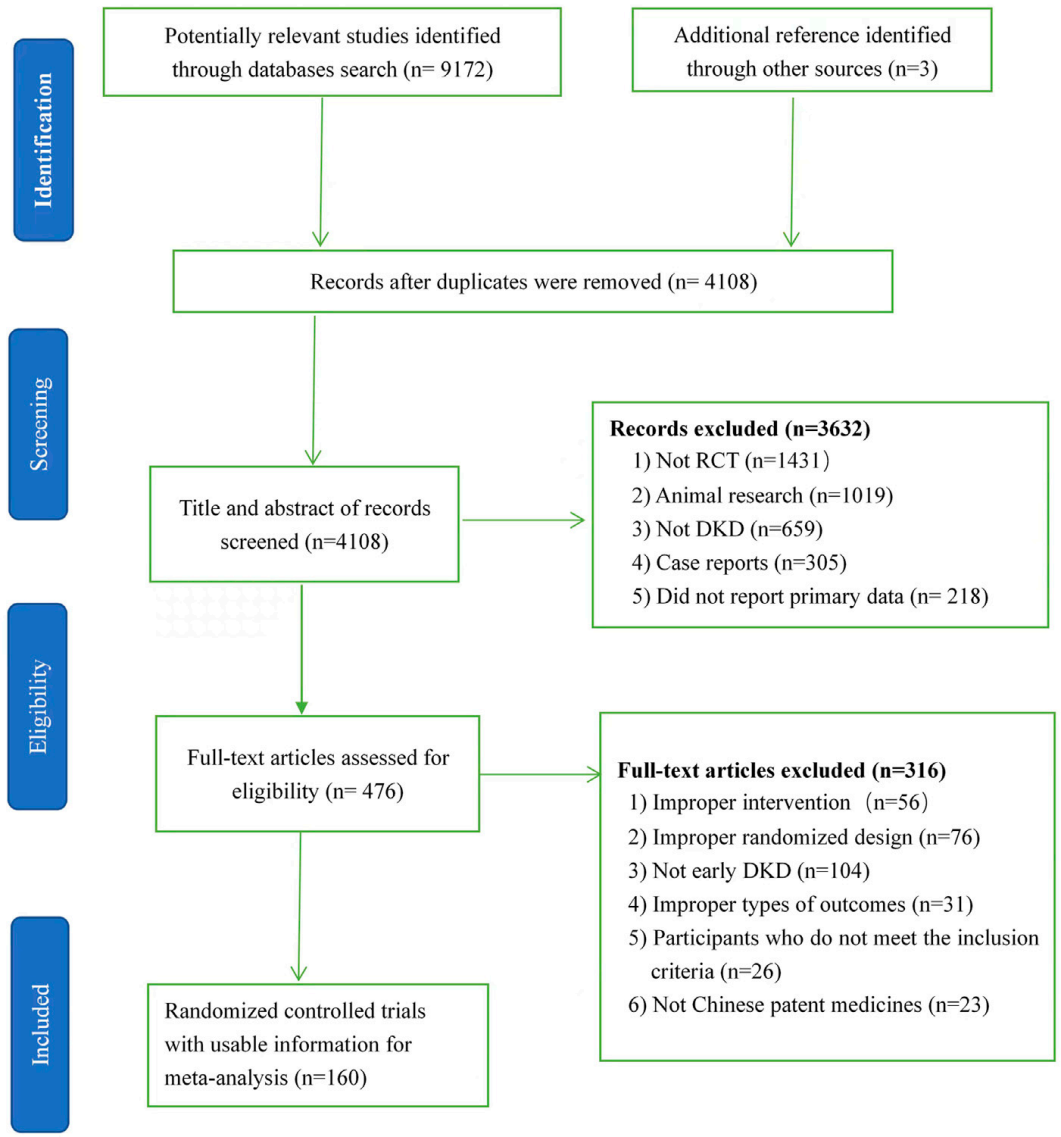
Flow chart of search and selection. DKD, diabetic kidney disease; RCT, randomized clinical trial.

Altogether, 9,175 studies were recognized, which included 5,067 reduplicated studies, and then 476 articles were identified after excluding 3,632 studies because of no RCT, animal research, no DKD, or case reports by means of the titles, key words, and abstracts. A total of 316 articles were removed after we skimmed 476 full texts because of improper interventions, randomized design, study object, outcomes or no CPMs. Ultimately, 160 two-armed RCTs (Supplementary Material S2) including 13,365 patients and nine kinds of CPMs were available for network meta-analysis. The nine Chinese patent medicines are Bailing capsule (BLC); Jinshuibao capsule (JSB); Huangkui capsule (HKC); Compound Xueshuantong capsule (CXC); uremic clearance granule (UCG); Shenyan Kangfu tablet (SYKFT); tripterygium glycosides (TG); Keluoxin capsule (KLX), and Shenshuaining tablet (SSNT). There are seven kinds of ACEIs (Ramipril (Ram); Benazepril (Ben); Perindopril (Per); Benazepril hydrochloride (Ben H); Enalapril (Ena); Captopril (Cap); and Enalapril maleate (Ena M)) and seven kinds of ARBs (Valsartan (Val); Irbesartan (Irb); Losartan (Los); Telmisartan (Tel); Losartan potassium (Los P); Olmesartan (Olm); and Candesartan (Can)) involved among enrolled 160 RCTs. All enrolled RCTs were performed in China and published between 2006 and 2022. The process of searching and selecting is presented in Figure 1.

### 3.2 Characteristics of included studies

Among enrolled 13,365 participants, 6,713 patients in the treatment group received nine CPMs (see Supplementary Table S3 for detailed characterizations of nine CPMs) in combination with an ACEI/ARB, and 6,652 participants in the control group took only an ACEI/ARB. In 160 eligible RCTs, four trials did not mention the concrete duration of treatment in their texts. The longest, shortest, and mean duration of treatment were 24, 2, and 11.6 weeks, respectively. In treatment groups, BLC was utilized in 45 RCTs and 2016 patients with highest frequency (45 RCTs, 2016 patients), JSB (41 RCTs, 1,692 patients), HKC (30 RCTs, 1,186 patients), UCG (13 RCTs, 470 patients), TG (4 RCTs, 215 patients), CXC (8 RCTs, 341 patients), SYKFT (5 RCTs, 234 patients), KLX (7 RCTs, 292 patients), and SSNT (7 RCTs, 267 patients). In control groups, there were 132 RCTs with 5,598 patients that used ARBs, and 28 RCTs with 1,054 patients that used ACEIs. As for outcomes, Scr was reported in 114 RCTs including nine CPMs and 9,368 patients with largest sample sizes (114 RCTs, 9 CPMs, 9,368 patients), UAER (90 RCTs, 9 CPMs, 7,105 patients), ORR (74 RCTs, 9 CPMs, 6,975 patients), AEs (67 RCTs, 9 CPMs, 5,841 patients), 24 h UTP (43 RCTs, 6 CPMs, 3,763 patients), TC (35 RCTs, 7 CPMs, 3,015 patients), Trig (38 RCTs, 7 CPMs, 3,341 patients), and HbA1c (34 RCTs, 6 CPMs, 2,751 patients). All patients received general treatments, such as management of blood pressure, blood glucose, and blood lipid. The basic characteristics of the 160 included RCTs are shown in Supplementary Table S4.

### 3.3 Quality assessment

The random grouping method has been mentioned among all eligible studies except 18 RCTs (11.25%). In 44 RCTs (27.5%), a specific randomization method was described, including 35 RCTs that used randomization number tables; 3 RCTs used the drawing of lots; 3 RCTs used computer serial number; 1 RCT used tossing a coin; 1 RCT used random envelope; and 1 RCT used block randomization. All of them were classified as “low risk” in random sequence generation. Except for two trials where several patients withdrew from the experiment due to the loss of follow-up, which were evaluated as “high risk” of bias in the complete outcome assessment domain, all other 158 RCTs were regarded as “low risk” of bias in the complete outcome assessment and selective reporting domain because of presenting complete data and no selecting outcomes to report. Nevertheless, due to a lack of sufficient information, RCTs were viewed as “unclear risks” because of possible performance biases, detection biases, and other biases. The risks of bias of each eligible study are shown in Supplementary Material S3.

### 3.4 Results of the NMA

#### 3.4.1 Primary outcomes

##### 3.4.1.1 UAER

**FIGURE 2:**
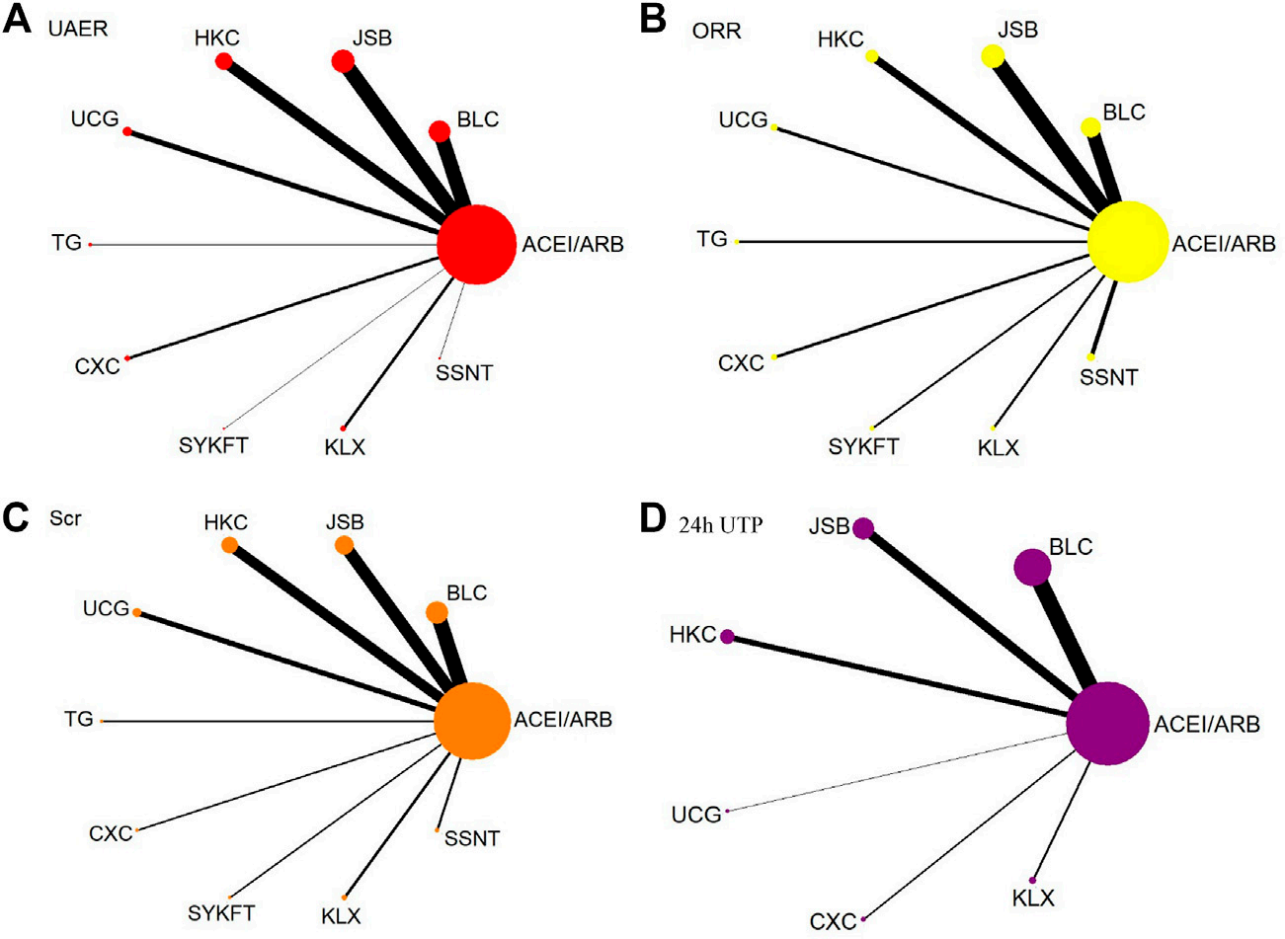
Network diagrams of primary outcomes. BLC, Bailing capsule; JSB, Jinshuibao capsule; HKC, Huangkui capsule; UCG, uremic clearance granule; TG, tripterygium glycosides; CXC, Compound Xueshuantong capsule; SYKFT, Shenyan Kangfu tablet; KLX, Keluoxin capsule; SSNT, Shenshuaining tablet; ACEI, angiotensin-converting enzyme inhibitor; ARB, angiotensin receptor blocker; UAER, urinary albumin excretion rate; ORR, overall response rate; Scr, serum creatinine; 24 h UTP, 24 h urinary total proteins.

UAER was reported in 90 publications involving 9 CPMs and 7,105 patients. Ten interventions were included: ACEI/ARB (90 trials, 3,534 patients), BLC (24 trials, 1,014 patients), JSB (25 trials, 991 patients), HKC (18 trials, 655 patients), UCG (9 trials, 314 patients), TG (2 trials, 135 patients), CXC (5 trials, 189 patients), SYKFT (1 trial, 36 patients), KLX (5 trials, 182 patients), and SSNT (1 trial, 55 patients). Network plot is presented in Figure 2A.

**FIGURE 3:**
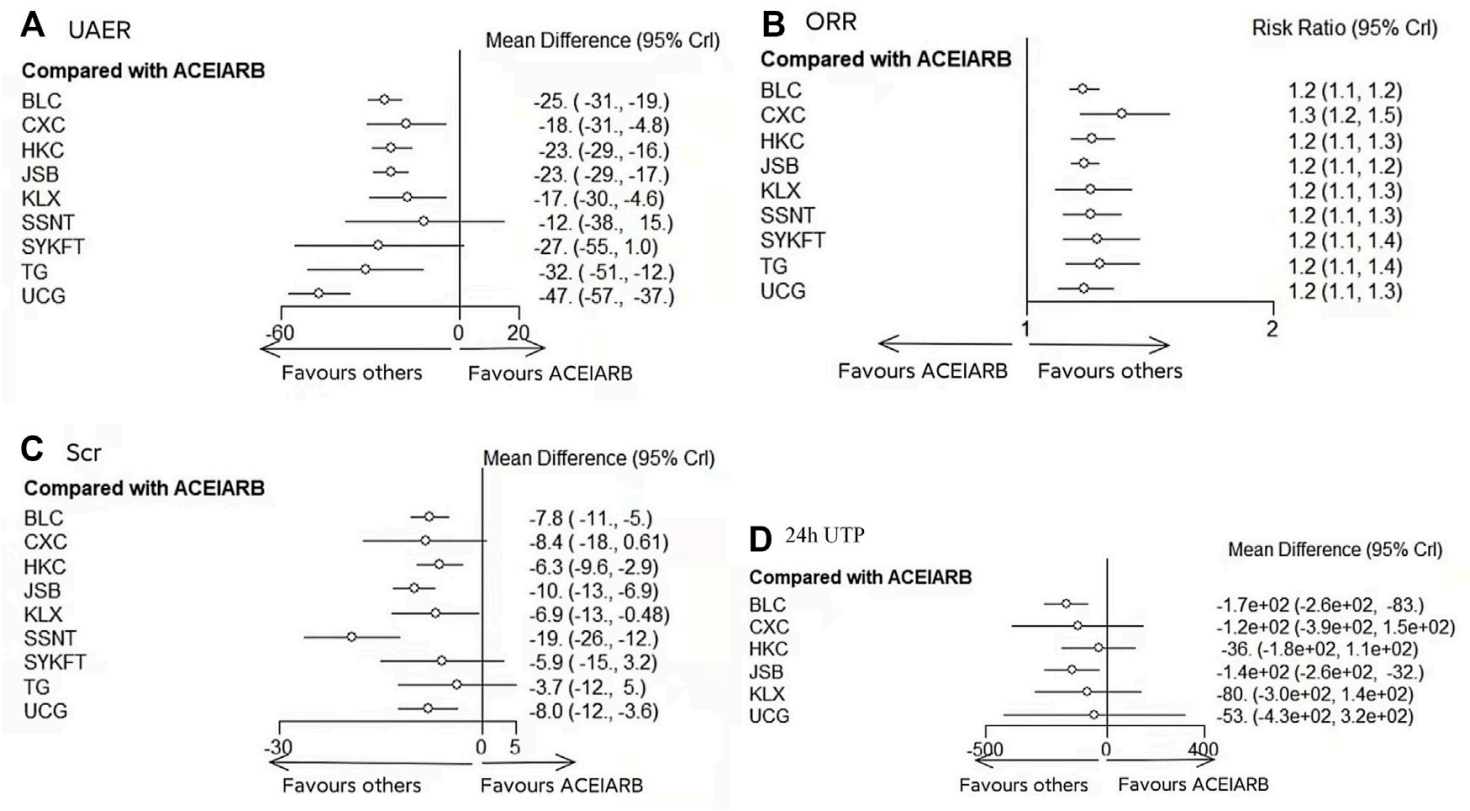
Forest plots of primary outcomes. BLC, Bailing capsule; JSB, Jinshuibao capsule; HKC, Huangkui capsule; UCG, uremic clearance granule; TG, tripterygium glycosides; CXC, Compound Xueshuantong capsule; SYKFT, Shenyan Kangfu tablet; KLX, Keluoxin capsule; SSNT, Shenshuaining tablet; ACEI, angiotensin-converting enzyme inhibitor; ARB, angiotensin receptor blocker; UAER, urinary albumin excretion rate; ORR, overall response rate; Scr, serum creatinine; 24 h UTP, 24 h urinary total proteins.

**FIGURE 4:**
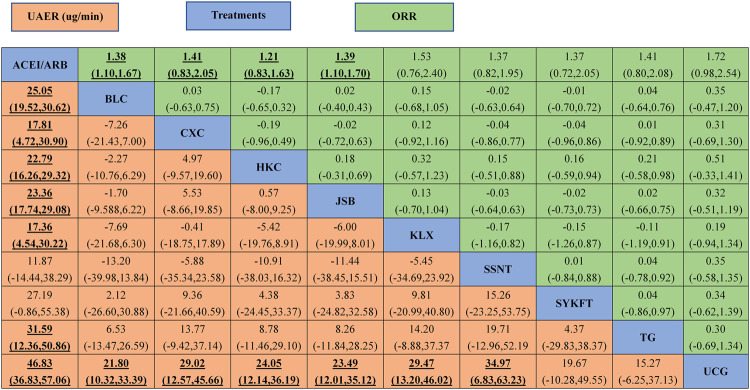
The league table of all comparisons of UAER and ORR. Data are RRs (95% CI) for ORR (upper-right quadrant) and MDs (95% CI) for UAER (lower-left quadrant) in the column-defining treatment compared with the row-defining treatment. Significant results are in bold and underscored. BLC, Bailing capsule; JSB, Jinshuibao capsule; HKC, Huangkui capsule; UCG, uremic clearance granule; TG, tripterygium glycosides; CXC, Compound Xueshuantong capsule; SYKFT, Shenyan Kangfu tablet; KLX, Keluoxin capsule; SSNT, Shenshuaining tablet; ACEI, angiotensin-converting enzyme inhibitor; ARB, angiotensin receptor blocker; UAER, urinary albumin excretion rate; ORR, overall response rate.

UCG (MD −47, 95%CI (−57, −37)), BLC (MD −25, 95%CI (−31, −19)), HKC (MD −23, 95%CI (−29, −16)), and JSB (MD −23, 95%CI (−29, −4.8)), KLX (MD −17, 95%CI (−30, −4.6)), CXC (MD −18, 95%CI (−31, −4.8)), TG (MD −32, 95%CI (−51, −12)) could be significantly superior to ACEI/ARB alone, except for SSNT (MD −12, 95%CI (−38, 15)), SYKFT (MD −27, 95%CI (−55, 1.0)). BLC, CXC, HKC, JSB, KLX, and SSNT demonstrated worse therapeutic effect compared with UCG in UAER with mean differences (MDs) of 21.80 (95% CI) (10.32, to 33.39), 29.02 (12.57, 45.66), 24.05 (12.14, 36.19), 23.49 (12.01, 35.12) 29.47 (13.20, 46.02), and 34.97 (6.83, 63.23) successively (see Figure 3A and Figure 4 for details).

The value of SUCRA about UAER was 98.0, 74.3, 62.6, 61.0, 54.0, 51.4, 35.3, 33.9, 27.2, and 2.4% for UCG, TG, BLC, SYKFT, JSB, HKC, CXC, KLX, SSNT, and ACEI/ARB alone, respectively. Specific details about the results of statistical analysis on the UAER are displayed in Table 1.

**TABLE 1:** SUCRA value of primary and secondary outcome indicators.

Treatment	UAER		ORR		Scr		24 h UTP		HbA1c		TC		Trig		AEs	
	SUCRA (%)	Mean rank	SUCRA (%)	Mean rank	SUCRA (%)	Mean rank	SUCRA (%)	Mean rank	SUCRA (%)	Mean rank	SUCRA (%)	Mean rank	SUCRA (%)	Mean rank	SUCRA (%)	Mean rank
ACEI/ARB	2.4	9.8	0.0	10.0	3.8	9.7	18.2	5.9	16.9	6.0	0.5	8.0	3.6	7.7	58.6	4.7
BLC	62.6	4.4	27.8	7.5	54.0	5.1	78.5	2.3	40.3	4.6	58.1	3.9	97.0	1.2	80.9	2.7
JSB	54.0	5.1	38.6	6.5	75.3	3.2	69.8	2.8	77.6	2.3	24.1	6.3	39.9	5.2	42.1	6.2
HKC	51.4	5.4	55.3	5.0	39.6	6.4	33.1	5.0	81.0	2.1	44.2	4.9	58.0	3.9	32.2	7.1
UCG	98.0	1.2	45.4	5.9	55.8	5.0	43.0	4.4	16.1	6.0	65.8	3.4	50.3	4.5	46.3	5.8
TG	74.3	3.3	62.9	4.3	26.9	7.6	NR	NR	NR	NR	75.8	2.7	49.2	4.6	13.4	8.8
CXC	35.3	6.8	91.9	1.7	57.1	4.9	59.7	3.4	45.8	4.3	41.3	5.1	50.9	4.4	37.2	6.6
SYKFT	61.0	4.5	64.3	4.2	40.7	6.3	NR	NR	NR	NR	NR	NR	NR	NR	51.8	5.3
KLX	33.9	6.9	55.7	5.0	47.7	5.7	47.7	4.1	NR	NR	90.2	1.7	51.0	4.4	54.5	5.1
SSNT	27.2	7.6	58.1	4.8	99.3	1.1	NR	NR	72.2	2.7	NR	NR	NR	NR	82.9	2.5

ACEI, angiotensin-converting enzyme inhibitors; ARB, angiotensin receptor blockers; BLC, Bailing capsule; JSB, Jinshuibao capsule; HKC, Huangkui capsule; UCG, uremic clearance granule; TG, tripterygium glycosides; CXC, Compound Xueshuantong capsule; SYKFT, Shenyan Kangfu tablet; KLX, Keluoxin capsule; SSNT, Shenshuaining tablet; UAER, urinary albumin excretion rate; ORR, overall response rate; Scr, serum creatinine; 24 h UTP, 24 h urinary total protein; HbA1c, glycosylated hemoglobin, type A1c; TC, total cholesterol; Trig, triglyceride; AEs, adverse effects.

##### 3.4.1.2 ORR

ORR was reported in 74 publications involving 9 CPMs and 6,975 patients. Ten interventions were included: ACEI/ARB (74 trials, 3,479 patients), BLC (18 trials, 989 patients), JSB (21 trials, 944 patients), HKC (11 trials, 461 patients), UCG (5 trials, 200 patients), TG (3 trials, 185 patients), CXC (4 trials, 165 patients), SYKFT (3 trials, 168 patients), KLX (3 trials, 139 patients), and SSNT (6 trials, 245 patients). Network plot is presented in Figure 2B.

As illustrated in Figure 3B, Compared with ACEI/ARB alone, all the remedies demonstrated better therapeutic effect in ORR with risk ratios (RRs) of 1.2 (95% CI) (1.1–1.2) for BLC, 1.3 (1.2–1.5) for CXC, 1.2 (1.1–1.3) for HKC, 1.2 (1.1–1.2) for JSB, 1.2 (1.1–1.3) for KLX, 1.2 (1.1–1.3) for SSNT, 1.2 (1.1–1.4) for SYKFT, 1.2 (1.1–1.4) for TG, and 1.2 (1.1–1.3) for UCG (See Figure 4 for more details).

CXC had the highest rate of ORR (SUCRA of 91.9%). It was followed by SYFKT (64.3%), TG (62.9%), SSNT (58.1%), KLX (55.7%), HKC (55.3%), UCG (45.4%), JSB (38.6%), and BLC (27.8%), while ACEI/ARB (0.00%) had the lowest SUCRA value (See Table 1 for details).

##### 3.4.1.3 Scr

Scr was reported in 114 publications involving 9 CPMs and 9,368 patients. Ten interventions were included: ACEI/ARB (114 trials, 4,659 patients), BLC (32 trials, 1,411 patients), JSB (27 trials, 1,154 patients), HKC (23 trials, 882 patients), UCG (12 trials, 440 patients), TG (3 trials, 185 patients), CXC (3 trials, 121 patients), SYKFT (3 trials, 98 patients), KLX (6 trials, 217 patients), and SSNT (5 trials, 201 patients). Network plot is presented in Figure 2C.

**FIGURE 5:**
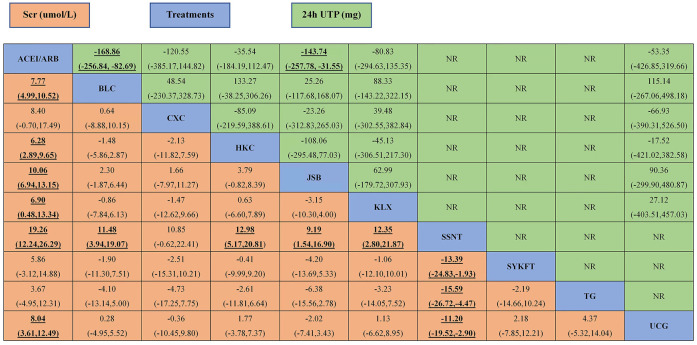
The league table of all comparisons of Scr and 24 h UTP. Data are MDs (95% CI) for 24 h UTP (upper-right quadrant) and MDs (95% CI) for Scr (lower-left quadrant) in the column-defining treatment compared with the row-defining treatment. Significant results are in bold and underscored. BLC, Bailing capsule; JSB, Jinshuibao capsule; HKC, Huangkui capsule; UCG, uremic clearance granule; TG, tripterygium glycosides; CXC, Compound Xueshuantong capsule; SYKFT, Shenyan Kangfu tablet; KLX, Keluoxin capsule; SSNT, Shenshuaining tablet; ACEI, angiotensin-converting enzyme inhibitor; ARB, angiotensin receptor blocker; Scr, serum creatinine; 24 h UTP, 24 h urinary total proteins.

According to the results of Figure 3C, compared with ACEI/ARB alone, all interventions, except for CXC, SYKFT, and TG, demonstrated a better therapeutic effect in Scr, with a mean difference (MD) of −7.8 (95% CI) (−11, −5) for BLC, −6.3 (−9.6, −2.9) for HKC, −10 (−13, −6.9) for JSB, −19 (−26, −12) for SSNT, −8 (−12, −3.6) for UCG, and −6.9 (−13, −0.48) for KLX according to the result of Figure 5C (See Figure 5 for more details).

The value of SUCRA about Scr was 99.3, 75.3, 57.1, 55.8, 54.0, 47.7, 40.7, 39.6, 26.9, and 3.8% for SSNT, JSB, CXC, UCG, BLC, KLX, SYKFT, HKC, TG, and ACEI/ARB alone, respectively. Specific details about the results of statistical analysis on the Scr are displayed in Table 1.

##### 3.4.1.4 24 h UTP

24 h UTP was reported in 43 publications involving 6 CPMs and 3,763 patients. Seven interventions were included: ACEI/ARB (43 trials, 1865 patients), BLC (19 trials, 856 patients), JSB (11 trials, 468 patients), HKC (7 trials, 274 patients), UCG (1 trial, 44 patients), CXC (2 trials, 97 patients), and KLX (3 trials, 159 patients). Network plot is presented in Figure 2D.

Compared with ACEI/ARB alone, only BLC (MD −170, 95% CI (−260, −83)) and JSB (MD −140, 95% CI (−260, −32) had greater effects on reducing 24 h UTP (Figure 3D). The other four CPMs presented no significant difference compared with ACEI/ARB in decreasing FBG with (MD) −120 95% CI (−390, 150) for CXC, −36 (−180, 110) for HKC, −80 (−300, 140) for KLX, and −53 (−430, 320) for UCG (See Figure 5 for more detailed results).

Unlike the results of NMA on other primary outcomes, BLC had the greatest rate of 24 h UTP (SUCRA of 78.5%) for patients with early DKD. It was followed by JSB, CXC, and KLX (SUCRA of 69.8, 59.7, and 47.7%, respectively). ACEI/ARB was the lowest in relieving 24 h UTP with SUCRA of 18.2% (See Table 1 for more information).

#### 3.4.2 Secondary outcomes

##### 3.4.2.1 TC

TC was reported in 35 publications involving 7 CPMs and 3,015 patients. A total of eight interventions were included. Network plot is presented in Supplementary Figure S1E. Compared with ACEI/ARB alone, all regimens, except for JSB, had significant advantages in decreasing TC with (MD) −0.88 95% CI (−1.4, −0.39) for BLC, −0.68 (−1.3, −0.077) for CXC, −0.73 (−1.0, −0.42) for HKC, −1.7 (−2.9, −0.46) for KLX, −1.2 (−2.0, −0.38) for TG, and −1.0 (−1.8, −0.29) for UCG according to Supplementary Figure S2E and Supplementary Figure S3. Based on the value of SUCRA, KLX was ranked first (SUCRA of 90.2%), and the worst treatment was ACEI/ARB, with 0.5% of SUCRA (See Table 1 for more detailed information).

##### 3.4.2.2 Trig

Trig was reported in 38 publications involving 7 CPMs and 3,341 patients. Network plot is presented in Supplementary Figure S1F. Compared with ACEI/ARB alone, four interventions, namely, BLC (−0.89 (−1.2, −0.53)), CXC (−0.39 (−0.70, −0.094)), HKC (−0.43 (−0.65, −0.22)), and JSB (−0.32, (−0.61, −0.019)), showed significant advantages in decreasing Trig, whereas other three interventions showed no significant advantages in reducing Trig according to Supplementary Figure S2F and Supplementary Figure S3. According to the value of SUCRA, BLC was superior to others in reducing Trig with SUCRA of 97.0% (See Table 1 for more information).

##### 3.4.2.3 HbA1c

HbA1c was reported in 34 publications involving 6 CPMs and 2,751 patients. Network plot is presented in Supplementary Figure S1G. Compared with ACEI/ARB alone, only two interventions, namely, JSB (−0.43 (−0.67, −0.20)) and HKC (−0.48 (−0.81, −0.13)), showed significant advantages in decreasing HbA1c according to Supplementary Figure S2G and Supplementary Figure S4. According to the value of SUCRA, HKC, JSB, and SSNT were ranked first, second, and third, with the SUCRA of 81.0, 77.9, and 88.7%, respectively (See Table 1 for more information).

### 3.4.3 Safety outcomes

AEs were reported in 67 publications involving 9 CPMs and 5,841 patients. Ten interventions were included: ACEI/ARB (67 trials, 2,900 patients), BLC (18 trials, 903 patients), JSB (8 trials, 315 patients), HKC (23 trials, 914 patients), UCG (7 trials, 285 patients), TG (2 trials, 135 patients), CXC (3 trials, 112 patients), KLX (3 trials, 159 patients), and SSNT (2 trials, 86 patients). Network plot is presented in Supplementary Figure S1H.

Compared with ACEI/ARB alone, three interventions, namely, BLC (0.39 (0.22, 0.66)), KLX (7.8 e-10 (4.6 e-24, 0.079)), and SYKFT (8.4 e-11 (9.0 e-44, 0.83)), showed significant advantages in AEs, whereas 95% CI of other six interventions all include one, implying that the difference was not statistically significant according to Supplementary Figure S3H and Supplementary Figure S4.

In terms of SUCRA, SSNT and BLC were ranked first and second, respectively, with SUCRAs of 82.9 and 80.9%. TG was ranked as the worst treatment, with the SUCRA of 13.4% (See Table 1 for more information). Gastrointestinal symptoms, including nausea, vomiting, gassiness and diarrhea, dizziness, headache, and thirst, were the common adverse events associated with most of the CPMs.

### 3.5 Cost-effectiveness analysis

Taking the Chinese market price as the standard and 3 months as a course of treatment, we counted the cost of nine kinds of CPMs for one course of treatment of early DKD (See Supplementary Table S5 for details). In our expense statistics, we selected the conventional dosage for each Chinese patent medicine after referring to drug instructions. It should be added that we took 60 kg as the reference standard weight when we calculated the cost of TG. According to the result, TG was ranked first with the least cost (108 RMB), followed by SYKFT (600 RMB) and CXC (648 RMB). HKC was ranked last with the most expenses (1440 RMB).

### 3.6 Heterogeneity

Overall, the *I* ^*2*^ values for all outcomes were less than 6%, which indicated low heterogeneity (See Supplementary Table S2 for more specific information).

### 3.7 Inconsistency test

We could not conduct an inconsistency test due to the lack of direct comparisons between different interventions and the lack of closed loops. For this reason, we applied the consistency model for statistical analysis.

### 3.8 Sensitivity analysis

In the outcomes of 24 h UTP and HbA1c, we deleted a single study from the pooled analysis each time to judge the reliability of the results on the basis of their heterogeneity. When omitting any single study, there was no significant impact on the overall effect sizes based on the sensitivity analysis, indicating our findings are robust in general (Supplementary Material S4).

### 3.9 Publication bias

In Egger’s tests, there were significant differences in 24 h UTP (*p* = 0.000), which indicates potential publication bias. For the other outcomes, no publication bias was found. In addition, comparison-adjusted funnel plots were also analyzed, and no significant publication bias was found (see Supplementary Material S5).

## 4 Discussion

In addition to being the most devastating and costly complication of diabetes worldwide, diabetic kidney disease (DKD) is also the leading cause of chronic kidney disease, especially in end-stage renal disease (ESRD) (Reutens, 2013). Hence, in this review, we focused on the outcomes most likely to be significant to patients with DKD, for example, UAER, ORR, Scr, 24 h UTP, and AEs. We ultimately included 160 RCTs involving 13,365 patients and 9 CPMs and then performed a network meta-analysis. As illustrated in our results, compared with ACEI/ARB alone, UCG and CXC might be the optimum selection for UAER and ORR; BLC showed the best curative effect on 24 h UTP and Trig; SSNT was the most effective on Scr and AEs; HKC showed the highest effectiveness in HbA1c; and KLX showed better effectiveness in TC. TG showed the highest cost-effectiveness among nine CPMs.

It has been found that many traditional Chinese medicines can improve the glucose and lipid metabolism of patients with DKD in varying degrees through multi-channel and multi-target, reduce the generation of cellular reactive oxygen species, inhibit the inflammatory response, slow down the degree of renal fibrosis, and improve renal function (Wu, 2022). The main components of BLC and JSB are Cordyceps sinensis. Cordyceps sinensis, known as “the king of the botanical drugs”, is one of the three great tonics in traditional Chinese medicine which can delay renal failure by regulating transforming growth factors, reducing blood glucose, anti-renal interstitial fibrosis, and anti-glomerulosclerosis (Zhan et al., 2012). The main components of HKC are the total flavonoids extracted from Abelmoschus manihot (Liu et al., 2015). Domestic and foreign studies have shown that HKC can reduce renal tubular injury, antagonize platelet aggregation, regulate immunity, reduce proteinuria, and protect glomerulus and renal tubular from injury by inhibiting NLRP3 inflammasome activation and blocking the TLR4/NF-κB signaling pathway (Lai et al., 2006; Han et al., 2019). UCG, composed of 16 traditional Chinese medicines such as *Astragalus* membranaceus, Atractylodes macrocephala, Poria cocos, Radix Polygoni Multiflori, Salvia miltiorrhiza, chrysanthemum, Rhizoma Pinelliae, licorice and so on, has been used in the clinic for more than 20 years (Zhang et al., 2021). Many clinical studies have also reported that UCG can not only improve renal function, anti-glomerulosclerosis, and anti-renal interstitial fibrosis but also protect endothelial function in patients with DKD (Hu et al., 2012). As a traditional Chinese medicine, TG is extracted from Tripterygium wilfordii Hook F. It has many pharmacological effects such as immune regulation, anti-inflammatory, anti-tumor, anti-fertility, and antibacterial effects (Li et al., 2014). The main components of CXC are Panax notoginseng, *Astragalus* membranaceus, Salvia miltiorrhiza, and Radix Scrophulariae, which can invigorate the circulation of blood, remove stasis of blood, and expand blood vessels (Huang and Sun, 2016). It is often combined with traditional antihypertensive drugs ACEI/ARB to assist in the treatment of DKD. SYKFT, the modified formula of Shenqi Pills derived from Synopsis of the Golden Chamber, are currently one of the few clinical available drugs for the treatment of chronic kidney diseases, with the effects of repairing damaged glomerular podocytes, regulating body immunity, reducing urine protein, and improving renal functions (Kou et al., 2014; Chen et al., 2021; Wang et al., 2022). KLX is composed of six traditional Chinese medicines: *Astragalus* membranaceus, Radix Pseudostellariae, leech, rhubarb, medlar, and Ligustrum lucidum. In recent years, KLX has been extensively used as an adjuvant drug in the treatment of DKD (Bo et al., 2019). Several clinical trials have reported that KLX can reduce proteinuria, blood creatinine, and urea nitrogen and protect the kidney (Wei et al., 2015). SSNT is a kind of commonly used CPM in the clinic. The prescription is composed of *Salvia miltiorrhiza*, *Radix pseudostellariae*, *Achyranthes bidentata*, rhubarb, tangerine peel, *Poria cocos*, safflower, *Coptis chinensis*, *Pinellia ternata*, and licorice. Modern pharmacological results show that SSNT can help regulate immune function in patients with DKD and have a good protective effect on renal tubulointerstitium (Wang et al., 2019b).

It is generally recognized that urinary albumin excretion rate (UAER) is the main diagnostic biomarker of DKD, especially for early DKD. In our review, UCG, BLC, HKC, JSB, KLX, CXC, and TG could be significantly superior to ACEI/ARB alone, except for SSNT (MD −12, 95%CI (−38, 15)), SYKFT (MD −27, 95%CI (−55, 1.0)). It should be noted that only one RCT observed the effects of SSNT and SYKFT in combination with ACEI/ARB on early DKD. Hence, more caution and prudence are needed to interpret the abovementioned result. At the same time, more RCTs with high-quality, large-sample, and multi-center about SSNT and SYKFT are needed to verify our findings in the future. As for the SUCRA, UCG combined with ACEI/ARB was ranked first among ten interventions with SUCRA 98.0%, which was consistent with the study conducted by Zhao et al. (2022).

For the outcome of ORR, we are surprised that all the remedies demonstrated better therapeutic effect than ACEI/ARBA alone in ORR. Li et al. published similar results when compared ACEI/ARB alone with JSB + ACEI/ARB in ORR (Li et al., 2020). Actually, these nine Chinese patent medicines, regardless of their monomers or prescriptions, have been proven beneficial in treating DKD in animal and human experiments in China. Traditional Chinese medical science believes that these nine Chinese patent medicines have important functions such as anti-inflammatory, regulating immune function, anti-lipid metabolism, reducing lipid peroxide, improving renal blood flow, and improving renal function. Hence, it is not difficult to understand such a result based on the theory of traditional Chinese medicine. In the past, it was thought that combined adjuvant therapy was undoubtedly superior to monotherapy, but now, our review provides evidence for this view.

For Scr, SSNT had the best effect on treating early DKD among all nine CPMs, which was different from the findings of Zhao et al. (2022). In their review, there was a significant decrease in SCr for CXC with the highest SUCRA. It is worth noting that only seven kinds of CPMs are involved, not including SSNT in their article. As far as we are concerned, this study is the first to include SSNT and compare its efficacy with other Chinese patent medicines. As an adjuvant treatment for chronic kidney failure, Cui et al. (2016) performed a meta-analysis of RCTs and determined that the SSNT group had better results than the control group in terms of clinical efficiency, such as Scr, which is consistent with our findings. In the treatment of diabetic kidney disease, Huang et al. (2022) performed a meta-analysis of SSNT in combination with Western medicine and showed that the combined use of SSNT and Western medicine not only significantly improved the total effective rate of DKD but also decreased levels of Scr, and UAER, which is highly consistent with our findings. Modern pharmacological studies have found that rhubarb, one of the components of SSNT, can not only inhibit hypermetabolism and renal compensatory hypertrophy and promote body excretion but also inhibit protein decomposition and promote its massive synthesis so as to improve azotemia (Xu, 2013; Wang et al., 2020). At present, there are few strong head-to-head clinical trials on SSNT; therefore, SSNT deserves more attention in the future based on the related findings of this review.

In terms of 24 h UTP and Trig, BLC in combination with ACEI/ARB could significantly reduce their levels and was regarded as the most beneficial intervention because of the highest SUCRA of 78.5 and 97.0%. BLC is prepared by low-temperature fermentation of *Cordyceps sinensis* strains isolated by the bioengineering method. In recent years, many studies (Yang et al., 2012; Tang et al., 2013) have confirmed the positive therapeutic effect of BLC on early DKD and is worthy of clinical promotion and application.

With regard to TC, relying on the highest SUCRA of 90.2%, KLX was ranked first and could remarkably reduce the level of TC. The theory of traditional Chinese medicine holds that KLX enhances Qi and nourishes Yin, promoting blood circulation and dispersing blood stagnation. DKD is linked to abnormal lipid metabolism, which plays an important role in the disease’s pathogenesis (Hirano, 2014). The importance of lipid-lowering therapy in delaying the progression of DKD is self-evident. Therefore, this review also included blood lipid indicators (TC and Trig) in the outcome indicators for analysis.

As for AEs, most publications did not observe adverse effects. Gastrointestinal symptoms, including nausea, vomiting, gassiness and diarrhea, dizziness, headache, and thirst, were the common adverse events associated with most of the CPMs in 67 trials that reported AEs. It is noteworthy that TG is ranked as the worst treatment in AEs, with the SUCRA of 13.4%, which suggests poor safety of TG. Therefore, clinicians should also pay attention to adverse reactions besides the curative effect when using TG in the clinical.

In terms of cost-effectiveness analysis, we roughly estimated the cost of using each Chinese patent medicine to treat DKD in China for 3 months. TG was ranked first with the least cost (108 RMB). This finding is not suitable for every country due to different national conditions and different prices of drugs. We all know that diabetic kidney disease, as a chronic disease, is a huge economic burden for both individuals and countries. Therefore, our results can be used as a reference for patients in low- and middle-income countries to a certain extent.

Taking into account the large sample size and the rich outcome indicators, this is the first study to comprehensively evaluate the efficacy and safety of nine CPMs for early DKD. It is also the first study to take into account the cost of drugs. Moreover, we registered our network meta-analysis in advance in the International Prospective Register of Systematic Reviews (CRD42022314843) and performed this network meta-analysis complying with the PRISMA guideline. In addition to providing data about the frequency of the most common adverse effects, the systematic review provides a statistical comparison of the basics of large amounts of data.

Despite this, there are a few inadequacies that should be considered when interpreting the results. First, 56 RCT studies (35%) clearly mentioned that included participants had type 2 diabetes. There were, however, 104 RCT studies that did not specify the type of diabetes participants had. Inconsistent types of diabetes among patients may lead to differing responses to drugs, causing deviations in data analysis. Second, for the same Chinese patent medicine, the dose taken by participants is not completely the same in the different RCT studies we included, which may have a certain impact on the final result. Third, the trials that we included about TG (4 trials), SYKFT (5 trials), and KLX (7 trials) were too few, perhaps leading to publication bias. Fourth, the quality of the enrolled trials was not high. A total of 116 trials (72.5%) did not provide specific randomization methods, and two (1.25%) trials did not report complete outcomes, leading to selection bias and incomplete outcome bias. In addition, the duration of treatment varied among the included studies, and some trials treated patients for a short time (only 2 weeks). Finally, we could not conduct an inconsistency test due to the lack of direct comparisons between different interventions, and all of the included RCTs were conducted in China.

## 5 Conclusion

In conclusion, our results show that UCG and CXC might be the optimum selection in UAER and ORR for patients with early DKD, and SSNT could be significantly superior to ACEI/ARB group in terms of Scr and AEs. BLC shows the best curative effect on 24 h UTP and Trig. TG shows the highest cost-effectiveness among nine CPMs. Although current estimates of the effects of most CPMs for DKD are significant and clinically relevant, enrolled reporting is not of high quality due to some information being missing. Thus, the use of these CPMs, including UCG, CXC, and BLC is worthy of further study, especially in terms of safety, and the evidence is currently insufficient to make any specific recommendations. For all of this, it is required to confirm these findings through more high-quality, large-sample, multi-center RCTs in the future.

## Data Availability

The original contributions presented in the study are included in the article/Supplementary Material; further inquiries can be directed to the corresponding author.

## Abbreviations

CPMs, Chinese patent medicines; DKD, diabetic kidney disease; NMA, network meta-analysis; RCTs, randomized controlled trials; BLC, Bailing capsule; JSB, Jinshuibao capsule; HKC, Huangkui capsule; UCG, uremic clearance granule; TG, tripterygium glycosides; CXC, Compound Xueshuantong capsule; SYKFT, Shenyan Kangfu tablet; KLX, Keluoxin capsule; SSNT, Shenshuaining tablet; ACEI, angiotensin-converting enzyme inhibitor; ARB, angiotensin receptor blocker; Scr, serum creatinine; UAER, urinary albumin excretion rate; ORR, overall response rate; HbA1c, glycosylated hemoglobin, type A1c; 24 h UTP, 24 h urinary total protein; TC, total cholesterol; Trig, triglyceride; AEs, adverse effects; RRs, risk ratios; MD, mean difference; SUCRA, surface under the cumulative ranking curve; DM, diabetes mellitus; GFR, glomerular filtration rate; ESRD, end-stage renal disease; TCM, traditional Chinese medicine.
