# Supplementary material for: Clinical efficacy, safety, and cost of nine Chinese patent medicines combined with ACEI/ARB in the treatment of early diabetic kidney disease: A network meta-analysis
Source: Front Pharmacol. 2022 Aug 22;13:939488. doi: 10.3389/fphar.2022.939488 (PMC9441488; doi:10.3389/fphar.2022.939488)
Supplement: Supplementary file 1 [file DataSheet1.pdf]

Supplementary Figure 1: Network diagrams of secondary outcomes

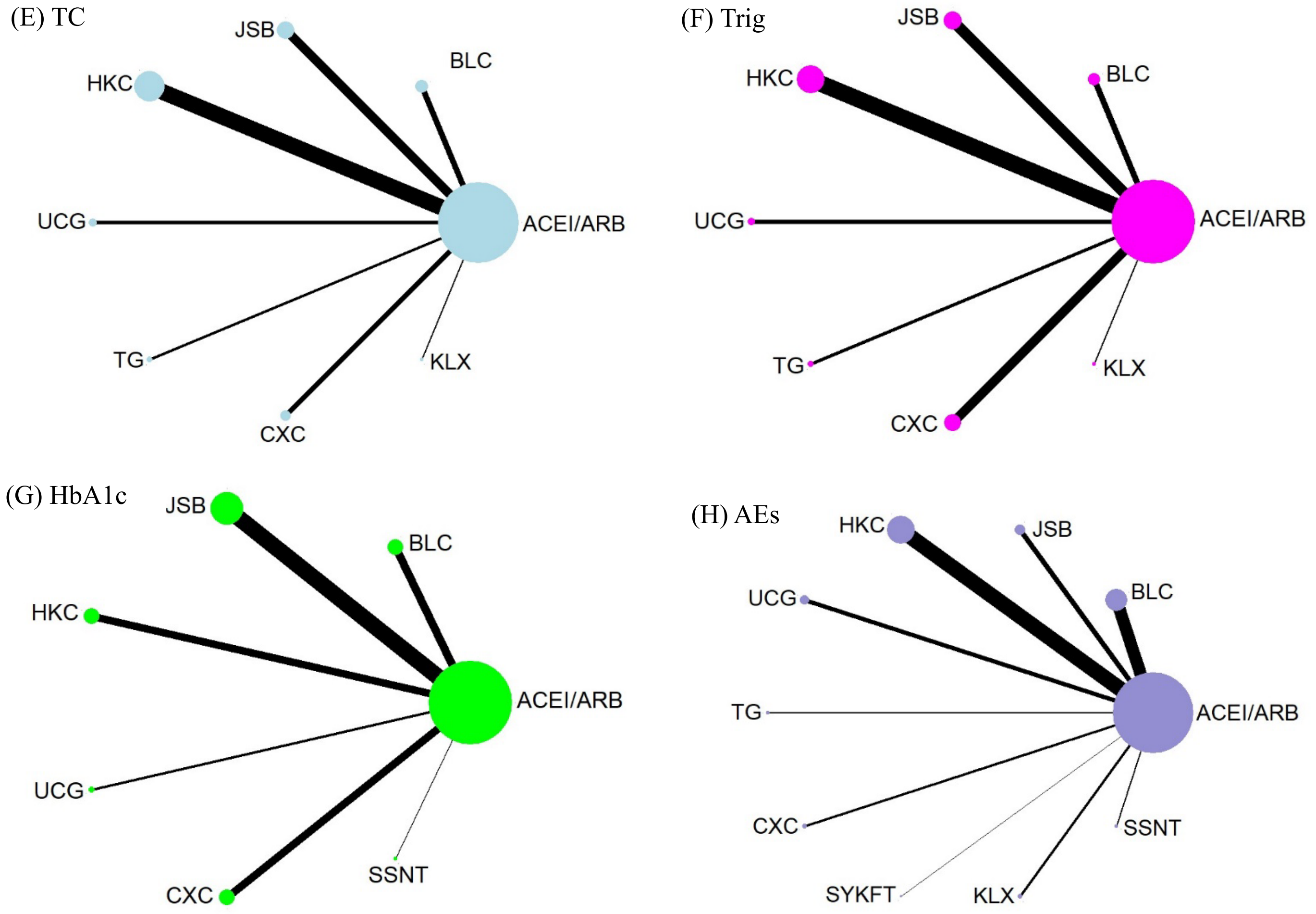

Supplementary Figure 1: Network diagrams of secondary outcomes. BLC, Bailing Capsule; JSB, Jinshuibao Capsule; HKC, Huangkui Capsule; UCG, Uremic Clearance Granule; TG, Tripterygium glycosides; CXC, Compound Xueshuantong Capsule; SYKFT, Shenyan Kangfu Tablet; KLX, Keluoxin Capsule; SSNT, Shenshuaining Tablet; ACEI, angiotensin-converting enzyme inhibitor; ARB, angiotensin receptor blocker; TC, Total cholesterol; Trig, Triglyceride; HbA1c, Glycosylated Hemoglobin, Type A1c; AEs, Adverse effects.

Supplementary Figure 2: Forest plots of Secondary outcomes

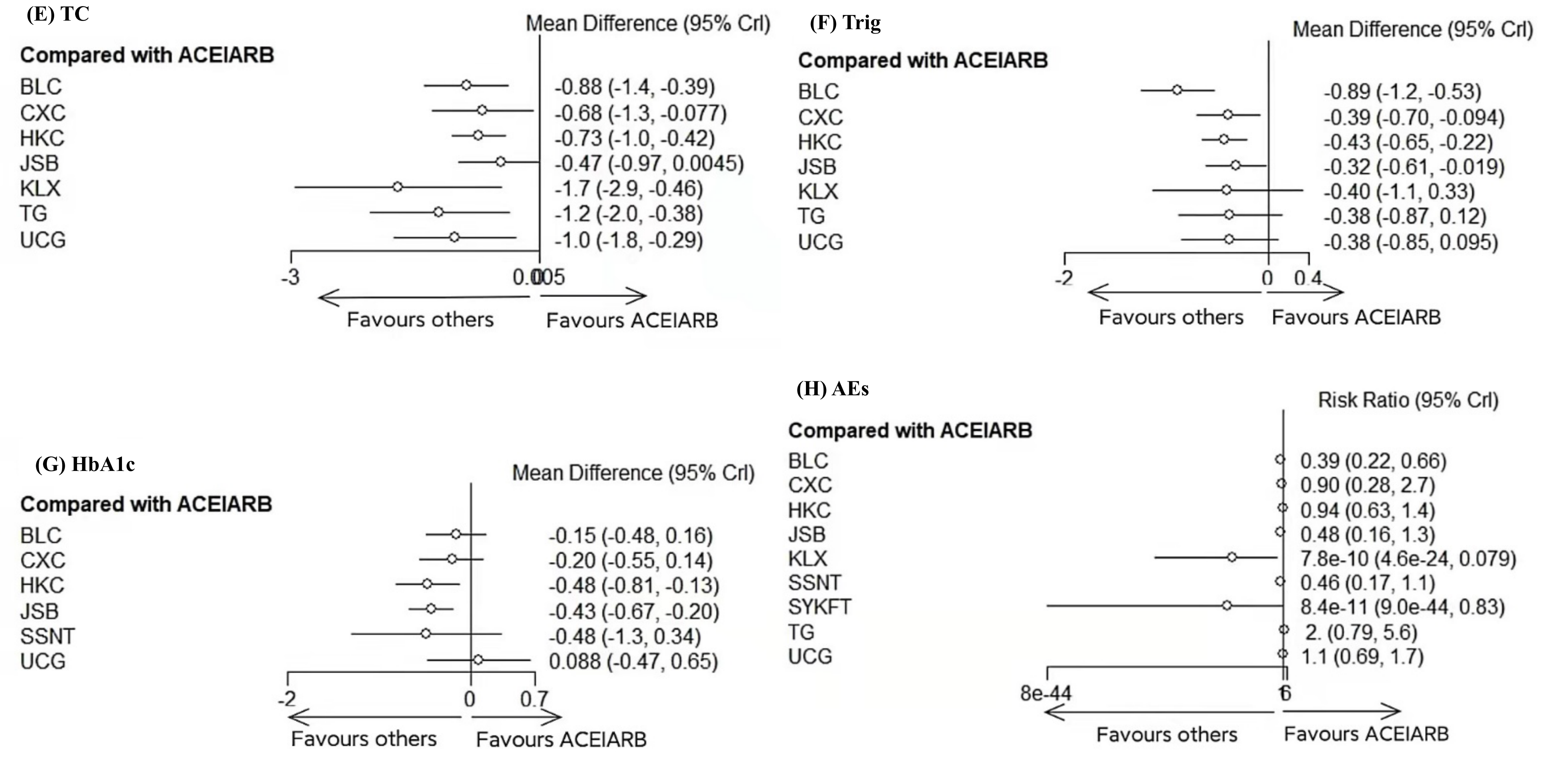

Supplementary Figure 2: Forest plots of Secondary outcomes. BLC, Bailing Capsule; JSB, Jinshuibao Capsule; HKC, Huangkui Capsule; UCG, Uremic Clearance Granule; TG, Tripterygium glycosides; CXC, Compound Xueshuantong Capsule; SYKFT, Shenyan Kangfu Tablet; KLX, Keluoxin Capsule; SSNT, Shenshuaining Tablet; ACEI, angiotensin-converting enzyme inhibitor; ARB, angiotensin receptor blocker; TC, Total cholesterol; Trig, Triglyceride; HbA1c, Glycosylated Hemoglobin, Type A1c; AEs, Adverse effects.

Supplementary Figure 3: League table of TC and Trig

TC (mmol/L)	Treatments	Trig (mmol/L)

**ACEI/ARB**	**<u>-0.89 (-1.25, -0.53)</u>**	**<u>-0.39 (-0.70, -0.09)</u>**	**<u>-0.43 (-0.65, -0.22)</u>**	**<u>-0.31 (-0.61, -0.02)</u>**	-0.40 (-1.13, 0.33)	-0.38 (-0.88, 0.12)	-0.38 (-0.85, 0.09)
**<u>0.89 (0.39, 1.39)</u>**	**BLC**	**<u>0.50 (0.02, 0.96)</u>**	**<u>0.46 (0.03, 0.87)</u>**	**<u>0.58 (0.11, 1.04)</u>**	0.49 (-0.33, 1.30)	0.52 (-0.11, 1.12)	0.52 (-0.09, 1.10)
**<u>0.69 (0.08, 1.29)</u>**	-0.20 (-0.99, 0.58)	**CXC**	-0.04 (-0.41, 0.33)	0.08 (-0.34, 0.50)	-0.01 (-0.80, 0.79)	0.01 (-0.56, 0.60)	0.01 (-0.55, 0.58)
**<u>0.73 (0.42, 1.046)</u>**	-0.15 (-0.75, 0.43)	0.05 (-0.64, 0.73)	**HKC**	0.12 (-0.24, 0.48)	0.03 (-0.73, 0.80)	0.06 (-0.49, 0.60)	0.06 (-0.47, 0.58)
**<u>0.47 (-0.01, 0.97)</u>**	-0.42 (-1.10, 0.29)	-0.21 (-0.98, 0.57)	-0.26 (-0.83, 0.33)	**JSB**	-0.09 (-0.88, 0.70)	-0.06 (-0.64, 0.52)	-0.06 (-0.63, 0.49)
**<u>1.70 (0.46, 2.94)</u>**	0.81 (-0.52, 2.15)	1.02 (-0.36, 2.39)	0.97 (-0.31, 2.25)	1.23 (-0.11, 2.55)	**KLX**	0.02 (-0.86, 0.91)	0.02 (-0.85, 0.89)
**<u>1.20 (0.37, 2.04)</u>**	0.319 (-0.65, 1.29)	0.52 (-0.51, 1.55)	0.47 (-0.41, 1.37)	0.74 (-0.24, 1.69)	-0.49 (-1.98, 1.00)	**TG**	-0.01 (-0.69, 0.69)
**<u>1.02 (0.29, 1.75)</u>**	0.14 (-0.75, 1.02)	0.34 (-0.61, 1.29)	0.29 (-0.50, 1.08)	0.56 (-0.34, 1.41)	-0.68 (-2.11, 0.76)	-0.18 (-1.29, 0.92)	**UCG**

Supplementary Figure 3: The league table of all comparisons of TC and Trig. Data are MDs (95% CI) for Trig (upper-right quadrant) and MDs (95% CI) for TC (lower-left quadrant) in the column-defining treatment compared with the row-defining treatment. Significant results are in bold and underscored. BLC, Bailing Capsule; JSB, Jinshuibao Capsule; HKC, Huangkui Capsule; UCG, Uremic Clearance Granule; TG, Tripterygium glycosides; CXC, Compound Xueshuantong Capsule; KLX, Keluoxin Capsule; ACEI, angiotensin-converting enzyme inhibitor; ARB, angiotensin receptor blocker; TC, Total cholesterol; Trig, Triglyceride.

Supplementary Figure 4: League table of AEs and HbA1c

AEs | **Treatments** | **HbA1c (%)**

**ACEIARB**	-0.15<br>(-0.48, 0.16)	-0.20<br>(-0.55, 0.14)	**-0.48**<br>**(-0.81, -0.13)**	**-0.43**<br>**(-0.67, -0.20)**	NR	NR	-0.48<br>(-1.30, 0.34)	NR	0.09<br>(-0.47, 0.65)
0.94<br>(0.41, 1.51)	**BLC**	-0.05<br>(-0.52, 0.42)	-0.32<br>(-0.77, 0.16)	-0.28<br>(-0.67, 0.12)	NR	NR	-0.33<br>(-1.20, 0.56)	NR	0.24<br>(-0.40, 0.89)
0.10<br>(-0.98, 1.26)	-0.84<br>(-2.08, 0.43)	**CXC**	-0.28<br>(-0.74, 0.23)	-0.23<br>(-0.64, 0.18)	NR	NR	-0.28<br>(-1.16, 0.61)	NR	0.29<br>(-0.36, 0.95)
0.06<br>(-0.33, 0.45)	-0.89<br>(-1.57, -0.21)	-0.05<br>(-1.26, 1.12)	**HKC**	0.05<br>(-0.38, 0.44)	NR	NR	-0.01<br>(-0.90, 0.87)	NR	0.57<br>(-0.11, 1.21)
0.73<br>(-0.25, 1.84)	-0.22<br>(-1.35, 1.01)	0.62<br>(-0.86, 2.16)	0.67<br>(-0.37, 1.86)	**JSB**	NR	NR	-0.05<br>(-0.89, 0.81)	NR	0.52<br>(-0.08, 1.13)
**20.97**<br>**(2.54, 53.74)**	**20.03**<br>**(1.61, 52.80)**	**20.96**<br>**(2.36, 53.63)**	**20.94**<br>**(2.50, 53.68)**	**20.20**<br>**(1.75, 53.04)**	**KLX**	NR	NR	NR	NR
0.78<br>(-0.11, 1.75)	-0.16<br>(-1.22, 0.95	0.68<br>(-0.79, 2.14)	0.73<br>(-0.26, 1.76)	0.05<br>(-1.35, 1.44)	**-20.15**<br>**(-53.00, -1.72)**	**SSNT**	NR	NR	NR
23.21<br>(0.19, 99.12)	22.30<br>(-0.77, 98.29)	23.09<br>(-0.05, 98.82)	23.16<br>(0.12, 99.09)	22.49<br>(-0.66, 98.30)	0.41<br>(-29.17, 57.82)	22.39<br>(-0.72, 98.29)	**SYKFT**	NR	0.57<br>(-0.42, 1.56)
-0.68<br>(-1.72, 0.24)	-1.63<br>(-2.80, -0.56)	-0.80<br>(-2.33, 0.64)	-0.74<br>(-1.84, 0.26)	-1.42<br>(-2.95, -0.07)	**-21.71**<br>**(-54.46, -3.16)**	-1.48<br>(-2.87, -0.18)	-23.91<br>(-99.78, -0.86)	**TG**	NR
-0.07<br>(-0.51, 0.37)	-1.01<br>(-1.74, -0.31)	-0.18<br>(-1.41, 1.01)	-0.12<br>(-0.73, 0.45)	-0.79<br>(-2.00, 0.27)	**-21.05**<br>**(-53.82, -2.62)**	-0.86<br>(-1.91, 0.15)	-23.28<br>(-99.22, -0.21)	0.62<br>(-0.41, 1.74)	**UCG**

Supplementary Figure 4: The league table of all comparisons of AEs and HbA1c. Data are MDs (95% CI) for HbA1c (upper-right quadrant) and RRs (95% CI) for AEs (lower-left quadrant) in the column-defining treatment compared with the row-defining treatment. Significant results are in bold and underscored. BLC, Bailing Capsule; JSB, Jinshuibao Capsule; HKC, Huangkui Capsule; UCG, Uremic Clearance Granule; TG, Tripterygium glycosides; CXC, Compound Xueshuantong Capsule; SYKFT, Shenyan Kangfu Tablet; KLX, Keluoxin Capsule; SSNT, Shenshuaining Tablet; ACEI, angiotensin-converting enzyme inhibitor; ARB, angiotensin receptor blocker; HbA1c, Glycosylated Hemoglobin, Type A1c; AEs, Adverse effects.

Supplementary File 1: PRISMA 2020 Checklist

Section and Topic	Item #	Checklist item	Location where item is reported
**TITLE**			
Title	1	Identify the report as a systematic review.	1
**ABSTRACT**			
Abstract	2	See the PRISMA 2020 for Abstracts checklist.	2-3
**INTRODUCTION**			
Rationale	3	Describe the rationale for the review in the context of existing knowledge.	4
Objectives	4	Provide an explicit statement of the objective(s) or question(s) the review addresses.	4-5
**METHODS**			
Eligibility criteria	5	Specify the inclusion and exclusion criteria for the review and how studies were grouped for the syntheses.	5-6
Information sources	6	Specify all databases, registers, websites, organisations, reference lists and other sources searched or consulted to identify studies. Specify the date when each source was last searched or consulted.	6
Search strategy	7	Present the full search strategies for all databases, registers and websites, including any filters and limits used.	6
Selection process	8	Specify the methods used to decide whether a study met the inclusion criteria of the review, including how many reviewers screened each record and each report retrieved, whether they worked independently, and if applicable, details of automation tools used in the process.	6 and Figure 1
Data collection process	9	Specify the methods used to collect data from reports, including how many reviewers collected data from each report, whether they worked independently, any processes for obtaining or confirming data from study investigators, and if applicable, details of automation tools used in the process.	5-7
Data items	10a	List and define all outcomes for which data were sought. Specify whether all results that were compatible with each outcome domain in each study were sought (e.g. for all measures, time points, analyses), and if not, the methods used to decide which results to collect.	5-6
	10b	List and define all other variables for which data were sought (e.g. participant and intervention characteristics, funding sources). Describe any assumptions made about any missing or unclear information.	5-7

Study risk of bias assessment	11	Specify the methods used to assess risk of bias in the included studies, including details of the tool(s) used, how many reviewers assessed each study and whether they worked independently, and if applicable, details of automation tools used in the process.	7 and Supplementary File 3
Effect measures	12	Specify for each outcome the effect measure(s) (e.g. risk ratio, mean difference) used in the synthesis or presentation of results.	7
Synthesis methods	13a	Describe the processes used to decide which studies were eligible for each synthesis (e.g. tabulating the study intervention characteristics and comparing against the planned groups for each synthesis (item #5)).	6-7
	13b	Describe any methods required to prepare the data for presentation or synthesis, such as handling of missing summary statistics, or data conversions.	6-7
	13c	Describe any methods used to tabulate or visually display results of individual studies and syntheses.	6-7
	13d	Describe any methods used to synthesize results and provide a rationale for the choice(s). If meta-analysis was performed, describe the model(s), method(s) to identify the presence and extent of statistical heterogeneity, and software package(s) used.	6-7
	13e	Describe any methods used to explore possible causes of heterogeneity among study results (e.g. subgroup analysis, meta-regression).	7-8 and Supplementary Table 1
	13f	Describe any sensitivity analyses conducted to assess robustness of the synthesized results.	Supplementary File 4
Reporting bias assessment	14	Describe any methods used to assess risk of bias due to missing results in a synthesis (arising from reporting biases).	Supplementary File 3
Certainty assessment	15	Describe any methods used to assess certainty (or confidence) in the body of evidence for an outcome.	-
**RESULTS**			
Study selection	16a	Describe the results of the search and selection process, from the number of records identified in the search to the number of studies included in the review, ideally using a flow diagram.	8 and Figure 1
	16b	Cite studies that might appear to meet the inclusion criteria, but which were excluded, and explain why they were excluded.	8 and Supplementary File 2
Study characteristics	17	Cite each included study and present its characteristics.	8-9 and Supplementary

			Table 2
Risk of bias in studies	18	Present assessments of risk of bias for each included study.	9 and Supplementary File 3
Results of individual studies	19	For all outcomes, present, for each study: (a) summary statistics for each group (where appropriate) and (b) an effect estimate and its precision (e.g. confidence/credible interval), ideally using structured tables or plots.	Figure 2 and Figure 3
Results of syntheses	20a	For each synthesis, briefly summarise the characteristics and risk of bias among contributing studies.	9
	20b	Present results of all statistical syntheses conducted. If meta-analysis was done, present for each the summary estimate and its precision (e.g. confidence/credible interval) and measures of statistical heterogeneity. If comparing groups, describe the direction of the effect.	9-13 and Figure3, Figure4
	20c	Present results of all investigations of possible causes of heterogeneity among study results.	13 and Supplementary File 4
	20d	Present results of all sensitivity analyses conducted to assess the robustness of the synthesized results.	Supplementary File 4
Reporting biases	21	Present assessments of risk of bias due to missing results (arising from reporting biases) for each synthesis assessed.	Supplementary File 3
Certainty of evidence	22	Present assessments of certainty (or confidence) in the body of evidence for each outcome assessed.	-
**DISCUSSION**			
Discussion	23a	Provide a general interpretation of the results in the context of other evidence.	14
	23b	Discuss any limitations of the evidence included in the review.	17
	23c	Discuss any limitations of the review processes used.	17
	23d	Discuss implications of the results for practice, policy, and future research.	17
**OTHER INFORMATION**			
registration and	24a	Provide registration information for the review, including register name and registration number, or state that the review was not registered.	1

protocol	24b	Indicate where the review protocol can be accessed, or state that a protocol was not prepared.	-
	24c	Describe and explain any amendments to information provided at registration or in the protocol.	-
Support	25	Describe sources of financial or non-financial support for the review, and the role of the funders or sponsors in the review.	18
Competing interests	26	Declare any competing interests of review authors.	18
Availability of data, code and other materials	27	Report which of the following are publicly available and where they can be found: template data collection forms; data extracted from included studies; data used for all analyses; analytic code; any other materials used in the review.	-

Supplementary File 2: Reference List of Included Studies

1. Chen J. The clinical studies on the prophylactic and therapeutic effects of bailing capsule in early diabetic kidney disease. Chinese Journal of Health Care and Medicine. 2009;11(5):371-3.
2. Dai X, Yuan L, Li Y. Clinical Efficacy and Safety of Huangkui Capsule Combined with Valsartan in the Treatment of Early Diabetes Nephropathy. Tianjin Journal of Traditional Chinese Medicine. 2017;34(03):163-4.
3. Ding H, Yu X. The Effect of Huangkui Capsule Combined with Valsartan on Serum Vaspin, NGAL and KIM-1 levels in Early Diabetes Nephropathy. Hebei Medical Journal. 2019;41(01):78-81.
4. Ding T. Clinical Effect of Irbesartan Combined with Jinshuibao in the Treatment of Diabetes Nephropathy. Journal of Mathematical Medicine. 2014;27(02):207-8.
5. Guan H, He K, Huan W, Lv L, Huang H, Wang B. Effect of Irbesartan with Bailing Capsules on Microalbuminuria-to-creatinine Ratio and High-sensitive C-reactive Protein in Patients with Early Diabetic Nephropathy. Chinese General Practice. 2010;13(26):2934-6.
6. He X. Clinical Analysis of Valsartan Combined with Bailing Capsule in Early Diabetes Nephropathy. Journal of Frontiers of Medicine. 2017;7(19):151-2.
7. Huang J, Zhou C. The Effect of Bailing Capsule Combined with Losartan on UMA and Lipid Metabolism in Patients with Early Diabetes Nephropathy. Chinese Journal of Biochemical Pharmaceutics. 2016;36(12):144-6+50.
8. Jiang T. Clinical Observation of Jinshuibao Capsule Combined with Ramipril in the Treatment of Early Diabetes Nephropathy. Journal of Practical Traditional Chinese Internal Medicine. 2021;35(02):39-42.
9. Jin S, Qin H. Clinical Observation of Huangkui Capsule Combined with Valsartan in the Treatment of Diabetes Nephropathy. China Practical Medicine. 2018;13(29):138-9.
10. Jin X. Clinical analysis of 50 cases of early diabetes nephropathy treated with Bailing Capsule Combined with valsartan capsule. Journal of New Chinese Medicine. 2016;48(08):99-101.
11. Lei S, Li J, Lai X, Zhang M, Zhang L, Xiong Y. The Effect of Irbesartan Combined with Jinshuibao in the Treatment of Non-hypertensive Diabetes Nephropathy. SHANDONG MEDICAL JOURNAL. 2009;49(1):98-9.
12. Li R, Lv Q, Yang X, Chen N. Therapeutic Effect of Niaoduqing Granule on Early Diabetes Nephropathy. The Journal of Medical Theory and Practice. 2016;29(11):1406-7+17.
13. Li Y. Clinical Effect of Huangkui Capsule Combined with Valsartan in the Treatment of

Diabetes Nephropathy. Journal of Community Medicine. 2014;12(15):36-7.

14. Li Z, Zhang G, Zheng S, Zheng J, Zhang Y, Yuan P. The Effects of Bailing Capsule Combined with Irbesartan on Oxidative stress, Inflammatory Response and Immune Function in Patients with Diabetes Nephropathy. Journal of Hainan Medical University. 2019;v.25;No.232(09):670-3.

15. Liang F, Guo J. Clinical Observation of Huangkui Capsule Combined with Valsartan in the Treatment of Diabetes Nephropathy. Shenzhen Journal of Integrated Traditional Chinese and Western Medicine. 2015;25(14):47-8.

16. Liu C, Li M. The Effect of Irbesartan Combined with Bailing Capsule on Urinary Albumin Excretion Rate and C-reactive Protein in Patients with Early Diabetes Nephropathy. Hebei Medical Journal. 2011;33(13):1990-1.

17. Liu J, Li H, Wang H, Li T, Zhang W. The Effect of Tripterygium Wilfordii Polyglycoside on MCP-1 in Blood and Urine of Patients with Diabetes Nephropathy. Modern Chinese Doctor. 2015;53(17):8-10.

18. Liu W, Zhang H. The Curative Effect of Jinshuibao Capsule Combined with Valsartan in the Treatment of Early Diabetes Nephropathy. Health Guide. 2017(30):267.

19. Luo F, Cao S, Sun X. Clinical Study of Bailing Capsule Combined with Irbesartan in the Treatment of Early Diabetes Nephropathy. Acta Chinese Medicine. 2011;26(04):466-7.

20. Luo J, Su X, Dai S, Cai J. Observation on the Efficacy of Bailing Capsule Combined with Irbesartan in the Treatment of Early Diabetic Nephropathy. Chinese And Foreign Medical Research. 2018;16(18):1-3.

21. Ma Y, Chen F, Chen B. The Effect of Bailing Capsule Combined with Irbesartan on IL-18 in Early Diabetes Nephropathy. China Pharmacist. 2011;14(07):1027-8.

22. Pan J, Shang S. Therapeutic Effect of Telmisartan Combined with Jinshuibao Capsule on Diabetes Nephropathy. Chinese Journal of Geriatric Care. 2016;14(01):40-1.

23. Qi M, Yu H, Li R. Clinical Analysis of Valsartan Combined with Huangkui Capsule in the Treatment of Early Diabetes Nephropathy. Inner MongoliaMedical Journal. 2016;48(03):296-8.

24. Qiu F, Fu A. Clinical Observation of Telmisartan Combined with Bailing Capsule in the Treatment of Early Type 2 Diabetic Nephropathy. Journal of New Chinese Medicine. 2016;48(9):52-3.

25. Shen S. The Effect of Valsartan Combined with Bailing Capsule on 86 Cases of Early Diabetes Nephropathy. Chinese Journal of Primary Medicine and Pharmacy. 2012;19(1):127-8.

26. Shen X, Wu J, Shao X, Zhu C, Zhou J, Lu K, et al. Effect of Jinshui Bao Capsule combined with Irbesartan Tablets on early diabetic nephropathy and its influence on urinary albumin and

oxidative stress. Modern Journal of Integrated Traditional Chinese and Western Medicine. 2018;27(21):2281-4,8.

27. Shi G, Su C, Cui L, Wang G, Lu D. The Therapeutic Effect of Perindopril Combined with Niaoduqing Granule on Diabetes Nephropathy. Clinical Journal of Traditional Chinese Medicine. 2014;26(07):679-81.

28. Tang W. Clinical Study on Huangkui Capsule Combined With Valsartan in the Treatment of Early Diabetes Nephropathy. Journal of Hubei University of Science and Technolog. 2017;31(5):396-9.

29. Wang F, Wang Z, Wu J. Clinical Study of Telmisartan Combined with Niaoduqing Granule in the Treatment of Early Diabetes Nephropathy. CHINESE JOURNAL OF CLINICAL RATIONAL DRUG USE. 2009;2(6):9-10.

30. Wang N, Zeng X. The Effect of Compound Xueshuantong Capsule Combined with Losartan Potassium on Early Diabetes Nephropathy. Journal of Clinical and Experimental Medicine. 2012;11(12):928-9+31.

31. Wang S, Wu C, Zhang H, Shi J, Chen B. The Effect of Bailing Capsule Combined with benazepril on early diabetes nephropathy. CHINA PHARMACIST. 2009;12(4):501-2.

32. Wang T, Zhang F, He Z, Long J. The Effects of Shenyankangfu Tablet on Renal Function, Inflammatory Factors and Hemorheology in Patients with Early Diabetes Nephropathy. Journal of North Sichuan Medical College. 2019;v.34;No.169(04):381-3+95.

33. Wang X, Gao F. Therapeutic Effect of Huangkui Capsule Combined with Valsartan on Diabetes Nephropathy. Journal of Hebei Medical University. 2010;31(06):733-4.

34. Wang Y, Yuan G. Clinical Observation of Bailing Capsule Combined with Telmisartan in the Treatment of Early Diabetes Nephropathy. Journal of Clinical Research. 2008(05):927-8.

35. Wei X, Ruan S. The Effect of Niaoduqing granule combined with olmesartan on early diabetes nephropathy and liver and kidney function. Chinese Journal of Gerontology. 2018;38(4):874-6.

36. Wei S. The Effect of Jinshuibao Capsule Combined with Benazepril on Early Type 2 Diabetes Nephropathy. HEILONGJIANG MEDICINE AND PHARMACY. 2010;33(1):50.

37. Wu P. The Effect of Niaoduqing granule Combined with Valsartan on Renal Function in Patients with Early Diabetes Nephropathy. Chronic Pathematology Journal. 2021;22(03):474-5+8.

38. Wu Q, Pan X. The Effect of Candesartan Axetil Combined with Jinshuibao on Early Type 2 Diabetic Nephropathy. Chinese Manipulation and Rehabilitation Medicine. 2016;7(21):42-4.

39. Wu L, Yan D, Jing S, Yu J, Geng J. The Effect of Valsartan Combined with Bailing Capsule on Urinary Albumin Excretion Rate in Patients with Early Diabetes Nephropathy. China

Pharmacist. 2014;17(09):1532-4.

40. Xiao Z, Sun H. The Effect of Huangkui Capsule Combined with Valsartan on Microalbuminuria in Patients with Early Diabetes Nephropathy. Modern Journal of Integrated Traditional Chinese and Western Medicine. 2010;19(03):263-4.

41. Xu L. Clinical Observation of Jinshuibao Combined with Irbesartan in the Treatment of Diabetes Nephropathy. Journal of Henan Medical College. 2015;27(03):355-6.

42. Yang C. The Effect of Jinshuibao Capsule Combined with Losartan on Early Diabetes Nephropathy. Modern Journal of Integrated Traditional Chinese and Western Medicine. 2013;22(01):65-6.

43. Yang G, Peng H, Liu D. The Effect of Bailing Capsule Combined with Irbesartan in the Treatment of diabetes nephropathy. Evaluation and Analysis of Drug-Use in Hospitals of China. 2016;16(10):1320-2.

44. Yang W. Clinical observation of Huangkui capsule combined with valsartan in the treatment of early diabetes nephropathy. Journal of Practical Traditional Chinese Medicine. 2020;v.36;No.324(01):76-7.

45. Ye F. The Effects of Tripterygium Wilfordii Polyglycoside Combined with Valsartan on Cytokines in Patients with Early Diabetes Nephropathy. Modern Journal of Integrated Traditional Chinese and Western Medicine. 2016;25(08):842-4.

46. Ye J, Liu Z, Li J, Lin H, Lu J. Clinical Observation of Bailing Capsule Combined with Candesartan in the Treatment of Early Diabetes Nephropathy. Internal Medicine. 2012;7(06):612-3.

47. Yu H, Shi H, Xiao L, Dong C. The Effect of Jinshuibao Combined with Olmesartan Kamedoxomil on Early Type 2 Diabetic Nephropathy. Contemporary Medicine. 2013(23):64-5.

48. Yun P, Gong T, Ma L, Xiao H. Clinical Study of Losartan Combined with Compound Xueshuantong Capsule in the Treatment of Early Diabetes Nephropathy. China Journal of Modern Medicine. 2013;23(04):67-70.

49. Zhang C, Zuo Z. The Effect of Jinshuibao Capsule Combined with Losartan Potassium on Patients with Early Diabetes Nephropathy. Journal of Clinical Medicine in Practice. 2014;18(05):84-6.

50. Zhou J, Jiang G, Li P. Observation on Treatment of Diabetes Nephropathy with Shenyan Kangfu Tablets Combined with Irbesartan. Xinjiang Journal of Traditional Chinese Medicine. 2012;30(05):28-30.

51. Zhou X, Jing H, Nie M. To Observe the Clinical Efficacy of Huangkui Capsule Combined with Valsartan in the Treatment of Diabetes Nephropathy. China Continuing Medical Education.

2016;8(12):180-2.
52. Zhu H. The Effect of Bailing Capsule Combined with Losartan in the Treatment of Diabetes Nephropathy. Journal of New Chinese Medicine. 2015;47(06):88-9.
53. Cai J, Wang Y, Cao P. Clinical Study of Niaoduqing granule in the treatment of Early Diabetes Nephropathy. Journal of Practical Diabetology. 2010;6(04):37-8.
54. Cai X, Huang B, Wang Y, Chen Z, Lin M. Clinical observation of Huangkui capsule combined with valsartan in the treatment of diabetes nephropathy. CONTEMPORARY MEDICINE. 2010;16(31):153-4.
55. Cao X. The Effect of Valsartan Combined with Bailing Capsule on Early Diabetes Nephropathy. China Modern Medicine. 2015;22(04):97-8+101.
56. Cao X, Zhang P, Yang J. Clinical observation on treatment of diabetic nephropathy with Jinshuibao capsules and valsartan. Chinese Journal of New Drugs. 2007;16(16):1303-6.
57. Cao Y. Clinical Observation of Benazepril Combined with Jinshuibao Capsule in the Treatment of Early Diabetes Nephropathy. Diabetes New World. 2019;22(6):184-5.
58. Chen F, Chen B, Shi J. Clinical Observation of Valsartan Combined with Bailing Capsule in Early Diabetes Nephropathy. China Pharmacist. 2010;13(4):551-2.
59. Chen Q, Zhang P, Liang H. Application of Benazepril Combined with Bailing Capsule in patients with Early Diabetes Nephropathy. China Practical Medicine. 2016;11(31):102-3.
60. Chen Q. The Effect of Losartan Potassium Combined with Jinshuibao on Early Diabetes Nephropathy. Chinese Journal of Urban and Rural Industrial Hygien. 2017;32(11):101-2.
61. Chen S, Cheng H, Yan H, Liu X. Clinical Study of Keluoxin Capsule Combined with Benazepril in the Treatment of Diabetes Nephropathy. Drugs & Clinic. 2020;35(9):1763-6.
62. Chen S. Clinical Observation of Shenshuaining Combined with Kesuya in the Treatment of Early Diabetes Nephropathy. Medical Information. 2018;31(5):20-2.
63. Chen Y. Clinical Observation of Losartan Potassium Combined with Shenshuaining Capsule in the Treatment of Early Diabetes Nephropathy. Drug Evaluation. 2020;17(2):34-5,7.
64. Chen Y, Ou Y, Wang Y. Clinical study on Huangkui capsule combined with ACEI and ARB in the treatment of early diabetes nephropathy. Journal of Bethune Military Medical College. 2015;13(01):14-6.
65. Chi P. Clinical Observation on 60 Cases of Diabetes Nephropathy Treated with Benazepril Combined with Jinshuibao Capsule. Chinese Manipulation and Rehabilitation Medicine. 2012;3(10):86-7.
66. Dai X, Liu D, Xiao Z, Shen X, Liu F, Wu H. Clinical Efficacy of Compound Xueshuantong Capsule Combined with Benazepril in the Treatment of Early Diabetes Nephropathy. Guangdong

Medical Journal. 2012;33(17):2676-8.
67. Deng S. Clinical Observation and Nursing of Huangkui Capsule Combined with Valsartan in the Treatment of Early Diabetes Nephropathy. Medical Information. 2014(24):78-.
68. Deng S. Clinical Observation of Shenshuaining Combined with Captopril in the Treatment of Early Diabetes Nephropathy. Nei Mongol Journal of Traditional Chinese Medicine. 2016;35(12):42-3.
69. Dou J, Weng X. The Clinical Studies on the Prophylactic and Therapeutic Effects of Bailing Capsule in diabetic nephropathy. Lishizhen Medicine and Materia Medica Research. 2006;17(6): 1121-1122.
70. Fan Y, Lin L, Fan G. Clinical Observation of Candesartan and Jinshuibao Capsules in the Treatment of Early Diabetes Nephropathy. Journal of Clinical Medical Literature (Electronic Edition). 2015;2(24):5003-4.
71. Feng Z. Clinical Evaluation of Jinshuibao Capsules Combined with Valsartan for Treating Early Diabetic Nephropathy with Micro-Inflammation in 43 Cases. China Pharmaceuticals. 2017;26(23):62-4.
72. Gao X. Clinical Observation of Losartan Potassium Combined with Jinshuibao Capsule in the Treatment of 76 Cases of Early type 2 Diabetic Nephropathy. Guide of China Medicine. 2018;16(06):102-3.
73. Ge Q. Therapeutic Effect of Benazepril Hydrochloride Combined with Jinshuibao Capsule on Diabetes Nephropathy. China Health Industry. 2011(20):59-.
74. Gu R. Clinical Observation of Huangkui capsule Combined with Valsartan in the Treatment of Early Diabetes Nephropathy. Journal of Cardiovascular Surgery. 2018;7(03):522-3.
75. Guan Y. Clinical Analysis of Irbesartan Combined with Nephritis Rehabilitation Tablets in the Treatment of Diabetes Nephropathy. Guide of China Medicine. 2020;18(23):53-4.
76. Guan C, Chen D, Duan X, Jiang Z, Chen L. The Effects of Bailing Capsule Combined with candesartan axetil on renal function, inflammatory factors and hemorheology in patients with early diabetes nephropathy. Chinese Archives of Traditional Chinese Medicine. 2021;39(05):247-50.
77. Guo G. Clinical Efficacy of Huangkui Capsule Combined with Valsartan in the Treatment of Early Diabetes Nephropathy. Journal of clinical rational drug use. 2015(21):142-3.
78. Guo T, Yi S. Observation on the curative effect of Benazepril Hydrochloride Tablet combined with Jinshuibao Capsule in the treatment of early diabetic nephropathy. China Modern Medicine. 2016;23(27):116-8.
79. He P, Lu B. The Effect of Valsartan Combined with Jinshuibao Capsule on Type 2 Diabetes Nephropathy. Medical Journal of Chinese People's Health. 2012;24(12):1452-3.

80. He X, Liu L, Lin B, Liu C. The Efficacy of Keloxin combined with Candesartan kamedoxomil in the Treatment of Diabetes Nephropathy. Chongqing Medicine. 2012;41(28):2983-5.
81. He Y. The Effects of Niaoduqing granule Combined with valsartan on renal function, Inflammatory Factors and Immune Function in Patients with Early Diabetes Nephropathy. Journal of New Chinese Medicine. 2021;53(10):60-3.
82. He Y. Clinical Observation on 40 Cases of Early Diabetes Nephropathy Treated with Huangkui Capsule Combined with Valsartan. Yunnan Journal of Traditional Chinese Medicine and Materia Medica. 2010;31(6):24-5.
83. Hu Q. Effect of Bailing Capsule on CRP and UMA level in patients with early diabetes nephropathy. Tianjin Journal of Traditional Chinese Medicine. 2007;24(4):286-8.
84. Hu W, Wei Y. Clinical Observation of Bailing Capsule Combined with Irbesartan on Early Diabetes Nephropathy. Chinese Journal of Traditional Medical Science and Technology. 2012;19(2):173-4.
85. Hu X. Clinical Evaluation of Jinshuibao Capsule Combined with Benazepril in the Treatment of Early Diabetes Nephropathy. Diabetes New World. 2019;22(7):174-5.
86. Hu Y, Gao M, Tan Y. Clinical Observation of Valsartan Combined with Huangkui capsule in the Treatment of Early Diabetes Nephropathy. Nei Mongol Journal of Traditional Chinese Medicine. 2016;35(14):77-9.
87. Hu Y. Clinical Efficacy of Bailing Capsule Combined with Irbesartan in the Treatment of Diabetes Nephropathy. Journal of Baotou Medical College. 2016;32(10):71-2.
88. Huang T, Sun H, Wu T. The Effect of Jinshuibao Capsule Combined with Perindopril on Renal Function in Elderly Patients with Early Diabetes Nephropathy. Herald of Medicine. 2010;29(7):890-2.
89. Jia Z. Clinical Observation on 38 Cases of Early Diabetes Nephropathy Treated with Huangkui Capsule Combined with Candesartan. Chinese Journal of Integrated Traditional and Western Nephrology. 2015;16(07):623-4.
90. Xie W. Effect of Irbesartan Combined with Jinshuibao on Early Type 2 Diabetic Nephropathy. Doctor. 2019;4(21):96-7.
91. Lan Y, Du L, Jiang C, Feng F. The Effect of Niaoduqing granule Combined with Losartan Potassium in the Treatment of Early Diabetes Nephropathy and its Effect on Oxidative Stress Indexes. Chinese Archives of Traditional Chinese Medicine. 2021;39(04):56-9.
92. Li H. Clinical Observation of Bailing Capsule Combined with Irbesartan in Early Diabetes Nephropathy. Harbin Medical Journal. 2013;33(4):291.

93. Li B, Peng H, Xiong D, Yi J, Chen H. Efficacy Observation of Treating Diabetic Nephropathy by Shenshuaining Granule Combined Telmisartan Tablet. Chinese Journal of Integrated Traditional and Western Medicine. 2015;35(2):142-6.
94. Li H. The Effect of Huangkui Capsule Combined with Losartan on 120 cases of Early Diabetes Nephropathy and its Effect on Serum Inflammatory Factors. Medical Journal of Chinese People's Health. 2017;29(24):84-5.
95. Li H, Lin S, Liu S. The Effect of RAAS Blocker Combined with Bailing Capsule in the Treatment of Early Diabetic Nephropathy and the Effect of Blood Glu-cose Level. Diabetes New World. 2022;25(2):191-4.
96. Li Q. Clinical effect of Bailing Capsule in the treatment of early diabetes nephropathy. Diabetes World. 2020;17(9):28.
97. Li Q, Cao M, Li K, Li J. The Effect of Niaoduqing Granule Combined with Irbesartan on Early Diabetes Nephropathy. Chinese Medical Innovations. 2021;18(16):16-20.
98. Li Q, He J. Clinical Observation of Huangkui Capsule Combined with Valsartan in the Treatment of Proteinuria in Early Stage of Type 2 Diabetes Mellitus. Chinese Journal of Integrated Traditional and Western Nephrology. 2010;11(2):142-3.
99. Li Y, Yao W. Irbesartan Combined with Jinshuibao Capsule in the Treatment of Early Diabetes Nephropathy. Chinese Journal of Modern Drug Application. 2013;7(1):66-7.
100. Liang Y. Observation on Curative Effect of Huangkui Capsule Combined with Valsartan Capsule in the Treatment of Early Diabetes Nephropathy. Journal of China Prescription Drug. 2014;12(10):25-6.
101. Lin M. Clinical Study of Irbesartan Tablets Combined with Shenyan Kangfu Tablets in the Treatment of Early Diabetes Nephropathy. China Health Care & Nutrition. 2013;23(3):1378-9.
102. Lin M, Cai X, Zeng L, Guo J. The Effect of Huangkui Capsule Combined with Irbesartan on Early Diabetes Nephropathy. Chinese Community Doctors. 2011;13(32):198.
103. Lin Z. Clinical observation of Bailing Capsule Combined with benazepril in the treatment of early diabetes nephropathy. Contemporary Medicine. 2016;22(24):150-1.
104. Liu C, Li M. Efficacy of Irbesartan Combined with Bailing Capsule in The Treatment of Early Diabetes Nephropathy. Hebei Medical Journal. 2011;33(11):1661-2.
105. Liu L. The Effect of Niaoduqing Granule Combined with Olmesartan in the Treatment of Early Diabetes Nephropathy and its Effect on Liver and Kidney Function. Chinese And Foreign Medical Research. 2021;19(02):62-4.
106. Liu L, Li L, He X, Tang Y, Shi J, Liu C, et al. Clinical Study of Keloxin Combined with Enalapril in the Treatment of Diabetes Nephropathy. Chinese Journal of Clinical Pharmacology

and Therapeutics. 2012;17(2):211-4.
107. Liu X, Zhang Z, Guan Y. The Effect of Irbesartan Combined with Niaoduqing Granule in the Treatment of Early Diabetes Nephropathy. The Journal of Practical Medicine. 2012;28(09):1567-8.
108. Liu X. Clinical Study of Cordyceps Preparation Combined with Benazepril in the Treatment of Early Diabetes Nephropathy. Asia-Pacific Traditional Medicine. 2011;7(12):135-6.
109. Liu Y. Clinical Observation of Huangkui Capsule Combined with Telmisartan on Micro Inflammation in Patients with Early Diabetes Nephropathy. Chinese Journal of Integrated Traditional and Western Nephrology. 2018;19(06):513-4.
110. Liu Y, Zhao L, Wang L, Gao A. Effect of Irbesartan Combined with Bailing Capsule on Microalbuminuria in Patients with Early Diabetes Nephropathy. Journal of Chinese Physician. 2011(02):261-3.
111. Lou P, Long X, Li L. Clinical Observation of Valsartan Combined with Bailing Capsule in the Treatment of Early Diabetes Nephropathy. The Journal of Practical Medicine. 2010;26(20):3780-2.
112. Luo J, Wu J. The Effect of Bailing Capsule Combined with Losartan in the Treatment of Early Diabetes Nephropathy. Diabetes World. 2021;18(12):78-80.
113. Lv F. Efficacy observation of treating early DN with Jin Shui Bao capsule. Clinical Journal of Chinese Medicine. 2012;4(13):23-4.
114. Lv M, Yu Y. Clinical Observation of Huangkui Capsule Combined with Valsartan in the Treatment of Diabetes Nephropathy. World Latest Medicine Information. 2015;15(A4):134+7.
115. Nie X. The Effect of Tripterygium Wilfordii Polyglycoside Combined with Valsartan in Patients with Diabetes Nephropathy. Chinese Journal of Convalescent Medicine. 2018;27(07):772-3.
116. Ou Y, Luo W, Chen Y. Clinical effect of Huangkui capsule combined with ACEI and ARB in the treatment of early diabetes nephropathy. China Modern Medicine. 2015;22(21):67-9+72.
117. Pan C, Zhang L. Clinical Observation of Shenshuaining Capsule Combined with Irbesartan in the Treatment of Early Diabetes Nephropathy. Chinese Journal of Convalescent Medicine. 2016;25(1):69-70.
118. Qi J, Yang J. Clinical Observation of Jinshuibao Capsule Combined with Western Medicine in the Treatment of Early Diabetes Nephropathy. Journal of North Pharmacy. 2018;15(9):55.
119. Qiao A. The Effect of Telmisartan Combined with Bailing Capsule on 62 Cases of Early Diabetes Nephropathy. China Practical Medicine. 2013;8(28):177-8.
120. Ren X, Yuan Q, Gan X, Li B, Shi M. The Effect of Bailing Capsule on Early Diabetes

Nephropathy and its effect on Hemorheology and Inflammatory Factors. Journal of Clinical Medicine in Practice. 2020;24(10):64-7.
121. Shen M, Tan L, Li Y. The Effect of Candesartan Combined With Jinshuibao Capsule on Early Type 2 Diabetes Nephropathy. Chinese Journal of Modern Drug Application. 2015(21):186-7.
122. Su J, Chen W. The effect of Compound Xueshuantong Capsule Combined with Jinshuibao Capsule on Early Diabetes Nephropathy. Nei Mongol Journal of Traditional Chinese Medicine. 2019;38(7):16-7.
123. Sun F. The Effect of Jinshuibao Combined with Irbesartan on Early Diabetes Nephropathy. China Health Standard Management. 2021;12(22):105-7.
124. Sun J. The Effect of Jinshuibao Capsule Combined with Losartan Potassium Tablets on Early Diabetes Nephropathy. Chinese Medicine Modern Distance Education of China. 2015;13(20):144-5.
125. Sun S. Clinical Observation of Irbesartan Combined with Shenyan Kangfu Tablets in the Treatment of 82 Cases of Diabetes Nephropathy. Guide of China Medicine. 2014(17):291-2.
126. Tang Y. Clinical Observation of Low-dose Telmisartan Combined with Jinshuibao in the Treatment of Early Diabetes Nephropathy with Normal Blood Pressure. Guide of China Medicine. 2011;9(27):210-1.
127. Tian X. The Effect of Bailing Capsule Combined with Valsartan on Patients with Early Diabetes Nephropathy. Practical Clinical Journal of Integrated Traditional Chinese and Western Medicine. 2019;19(07):18-9.
128. Wang A, Zhang T, Wu W, Tang W, Wang Y, Han X. The Effect of Jinshuibao Capsule Combined with Candesartan Axetil Tablets on Early Diabetes Nephropathy. Contemporary Medicine Forum. 2020;18(12):58-9.
129. Wang G, Wu Z, Guo X. Therapeutic Effect of Jinshuibao Capsule on Early Diabetes Nephropathy. Journal of Hebei Traditional Chinese Medicine and Pharmacology. 2006;21(2):9-10.
130. Wang J, Wang J, Yang L, Liu L. The Effect of Bailing Capsule Combined with Losartan on Early Diabetes Nephropathy. The Medical Forum. 2015;19(21):2890-1.
131. Wang K, Li Y, Dai Z. The Effect of Bailing Capsule Combined with Valsartan in the Treatment of Diabetes Nephropathy and its Effect on UAR and UAER. Modern Practical Medicine. 2020;32(02):171-3.
132. Wang L. The Effect of Candesartan Axetil Tablets Combined with Jinshuibao in the Treatment of Early Nephropathy in Type 2 Diabetes. Nei Mongol Journal of Traditional Chinese Medicine. 2018;37(03):43-4.
133. Wang X. Clinical Observation of Valsartan Combined with Bailing Capsule in the Treatment

of 60 Cases of Early Diabetes Nephropathy. Practical Clinical Journal of Integrated Traditional Chinese and Western Medicine. 2015;15(11):13-5.
134. Wu N, Yan Y, Gu J. Clinical Observation of Compound Xueshuantong Capsule Combined with Valsartan in the Treatment of Diabetes Nephropathy. China Health Industry. 2013;v.10;No.160(05):68+70.
135. Xiao X, Zhou R, Chen F. Clinical Observation on 40 Cases of Early Diabetes Nephropathy Treated with Niaoduqing Granule. Journal of New Chinese Medicine. 2011;43(08):48-9.
136. Xiao Y. Clinical Study of Bailing Capsule Combined with Irbesartan in the Treatment of Early Diabetes Nephropathy. Journal of Practical Traditional Chinese Medicine. 2021;37(08):1356-8.
137. Xu J. Effect of Irbesartan Combined with Niaoduqing Granule on Early Diabetes Nephropathy. Nei Mongol Journal of Traditional Chinese Medicine. 2016;35(06):44-5.
138. Xu W, Chen H, Cao C, Zhan H, Pan H. Clinical observation of Huangkui capsule combined with irbesartan on oxidative stress in patients with early diabetes nephropathy. Chinese Journal of Integrated Traditional and Western Nephrology. 2013;14(10):910-1.
139. Xu X. The Effect of Integrated Traditional Chinese and Western Medicine on Early Diabetes Nephropathy. Oriental Medicated Diet. 2020(18):109.
140. Yang P, Sun S, Wei Y. Comparison of Effects of Compound Xueshuantong Capsule and Benazepril on Serum Inflammatory Cytokines in Early Diabetic Nephropathy. Tianjin Medical Journal. 2014(7):716-8.
141. Yang T. Clinical Efficacy of Tripterygium Wilfordii Polyglycoside Tablets Combined with Valsartan in the Treatment of Early Diabetes Nephropathy. Qingdao Medical Journal. 2013;45(04):270-1.
142. Yang X, Yang K, Gao W, Cao X. Clinical Observation of Enalapril Combined with Jinshuibao Capsule in the Treatment of Diabetes nephropathy. Hebei Medical Journal. 2012;34(6):938-9.
143. Yu H. The Effect of Jinshuibao Capsule Combined with Candesartan Axetil Tablets in the Treatment of Early Diabetes Nephropathy. Modern Chinese Doctor. 2022;60(05):34-7.
144. Yu J, Liang Y. The Effect of Keluoxin Capsule Combined with Valsartan on Serum Oxidative Stress in Patients with Diabetes Nephropathy. Journal of Huaihai Medicine. 2021;39(4):406-8.
145. Yuan J, Liu L, Suo X, Wang X. Clinical Study of Shenshuaining Granule Combined with Valsartan in the Treatment of Early Diabetes Nephropathy. Drugs & Clinic. 2019;34(6):1784-8.
146. Yuan Y. Clinical Observation of Telmisartan Combined with Bailing Capsule in the Treatment of 51 Cases of Early type 2 Diabetes Nephropathy. Journal of Aerospace Medicine.

2017;28(03):270-2.
147. Zhang L. Jinshuibao Capsule Combined with Candesartan Kamedoxomil Tablets in the Treatment of 40 Cases of Early Type 2 Diabetic Nephropathy. Zhejiang Journal of Traditional Chinese Medicine. 2010;45(10):779.
148. Zhang M. Clinical Study of Shenshuaining Granule Combined with Valsartan in the Treatment of Early Diabetes Nephropathy. Diabetes World. 2021;18(1):20.
149. Zhang Q. Clinical Observation of Valsartan Combined with Bailing Capsule in the Treatment of Early Diabetes Nephropathy. Chinese Journal of Clinical Research. 2020;28(9):49-50.
150. Zhang R. Clinical Observation of Valsartan Capsule Combined with Huangkui Capsule in the Treatment of Diabetes Nephropathy. Journal of clinical rational drug use. 2016;9(25):23-4.
151. Zhang X. Clinical Study on Keluoxin Capsule Combined with Irbesartan Tablets in the Treatment of Early Diabetes Nephropathy. International Journal of Traditional Chinese Medicine. 2016;38(10):884-7.
152. Zhang Y. Clinical Effect of Jinshuibao Capsule Combined with Irbesartan Tablets in the Treatment of Early Diabetes Nephropathy. Chinese Journal of Medical Device. 2021;34(10):105-6.
153. Zhang Y, Zhang B. Clinical Observation of Jinshuibao Capsule Combined with Irbesartan Tablets in the Treatment of Diabetes Nephropathy. Journal of Practical Traditional Chinese Medicine. 2019;35(11):1359-60.
154. Zhang Z. Clinical research on early diabetic nephropathy treated with therapy of combination of Chinese traditional and Western medicine. Journal of Medical Forum. 2019;40(8):85-6,9.
155. Zhang Z, Li Y, Miao R. The Effect of Candesartan Axetil Tablets Combined with Jinshuibao on Early Nephropathy of Type 2 Diabetes. Journal of Guangdong Pharmaceutical university. 2014;30(02):241-4.
156. Zhao L. Clinical Analysis of Keluoxin Capsule Combined with Benazepril Hydrochloride Tablets in the Treatment of Diabetes Nephropathy. Women´s Health. 2021(16):110.
157. Zheng J. Clinical Observation of Irbesartan Combined with Bailing Capsule in the Treatment of Type 2 Diabetes Nephropathy. China Modern Medicine. 2011;18(24):66-7.
158. Zhou G. Clinical Study of Keluoxin Combined with Irbesartan in the Treatment of Proteinuria in Early Diabetes Nephropathy. CHINESE JOURNAL OF CLINICAL RATIONAL DRUG USE. 2013;6(34):20-1.
159. Zhou S. Clinical Study of Compound Xueshuantong Capsule Combined with Irbesartan Tablets in the Treatment of Diabetes Nephropathy. Shanxi Medical Journal. 2015;44(08):944-6.
160. Zhu H. Clinical Efficacy and Safety of Huangkui Capsule Combined with Valsartan in the

Treatment of Early Diabetes Nephropathy. Diabetes New World. 2020;23(21):176-7+80.

Supplementary File 3: Risk of bias table for included studies

eTable1. Quality evaluation of enrolled RCTs according to Cochrane Handbook.

Study	Random sequence generation	Random concealment	Blinding of participants and personnel	Blinding of outcome assessment	Incomplete outcome data	Selective reporting	Other bias
Chen J 2009	The trial only reported "randomized"	No information was provided.	No information was provided.	No information was provided.	The trial did not report any dropouts.	Results of all primary outcomes were reported.	Unclear
Dai X 2017	The trial used a random number–producing algorithm in central computer systems for simple randomization.	Allocation concealment was done by sealed sequentially numbered opaque envelopes. They were consecutively numbered and were provided to the patients according to the number allocated	This article was a double-blind study	Data were collected in a paper case report form and entered into a database.	The trial did not report any dropouts.	Results of all primary outcomes were reported.	Unclear
Ding HH 2019	The trial only reported "randomized"	No information was provided.	No information was provided.	No information was provided.	The trial did not report any dropouts.	Results of all primary outcomes were reported.	Unclear
Ding T 2014	Patients were randomly assigned to one of two treatment groups	The subject numbers were assigned sequentially as each subject entered the study.	Both the patients, treating physicians, and individuals were blinded to patient treatment assignments.	No information was provided.	The trial did not report any dropouts.	Results of all primary outcomes were reported.	Unclear
Guan HB 2010	Patients were randomly assigned to one of two treatment groups	No information was provided.	No information was provided.	No information was provided.	The trial did not report any dropouts.	Results of all primary outcomes were reported.	Unclear
He XJ 2017	Patients were randomly assigned to one of two treatment groups	No information was provided.	No information was provided.	No information was provided.	The trial did not report any dropouts.	Results of all primary outcomes	Unclear

						were reported.	
Huang JY 2016	Patients were divided into one of two groups with the method of random sampling	The person doing the randomization was blinded.	This article was a double-blind study.	No information was provided.	The trial did not report any dropouts.	Results of all primary outcomes were reported.	Unclear
Jiang T 2021	The trial only reported "randomized"	No information was provided.	No information was provided.	No information was provided.	The trial did not report any dropouts.	Results of all primary outcomes were reported.	Unclear
Jin S 2018	The trial only reported "randomized"	No information was provided.	No information was provided.	No information was provided.	The trial did not report any dropouts.	Results of all primary outcomes were reported.	Unclear
Jin XB 2016	Participants were randomly assigned following computer-based random numbers to one of the two treatment groups.	An analyst, without clinical involvement in the trial, kept randomization lists concealed.	Both the patients, treating physicians, and individuals were blinded to patient treatment assignments.	Data were collected in a paper case report form and entered into a database.	The trial did not report any dropouts.	Results of all primary outcomes were reported.	Unclear
Lei SH 2009	The trial only reported "randomized"	No information was provided.	No information was provided.	No information was provided.	The trial did not report any dropouts.	Results of all primary outcomes were reported.	Unclear
Li RN 2016	The trial only reported "randomized"	No information was provided.	No information was provided.	No information was provided.	The trial did not report any dropouts.	Results of all primary outcomes were reported.	Unclear
Li YT 2014	The trial only reported "randomized"	No information was provided.	No information was provided.	No information was provided.	The trial did not report any dropouts.	Results of all primary outcomes	Unclear

						were reported.	
Li Z 2019	The trial only reported "randomized"	No information was provided.	No information was provided.	No information was provided.	The trial did not report any dropouts.	Results of all primary outcomes were reported.	Unclear
Liang F 2015	Patients were randomly assigned to one of two treatment groups	The subject numbers were assigned sequentially as each subject entered the study.	No information was provided.	Data were collected by an analyst, with no clinical involvement in the trial.	The trial did not report any dropouts.	Results of all primary outcomes were reported.	Unclear
Liu CP 2011	Patients were randomly assigned to one of two treatment groups	No information was provided.	No information was provided.	No information was provided.	The trial did not report any dropouts.	Results of all primary outcomes were reported.	Unclear
Liu JL 2015	The trial only reported "randomized"	No information was provided.	No information was provided.	No information was provided.	The trial did not report any dropouts.	Results of all primary outcomes were reported.	Unclear
Liu WY 2017	Patients were randomly assigned to one of two treatment groups	Allocation concealment was performed by enclosing assignments in sequentially numbered, opaque-closed envelopes.	The recruiting and treating doctors, as well as the patients, were blinded on the type of treatment throughout the medication period.	No information was provided.	The trial did not report any dropouts.	Results of all primary outcomes were reported.	Unclear
Luo F 2011	Patients were randomly assigned to one of two treatment groups	No information was provided.	No information was provided.	No information was provided.	The trial did not report any dropouts.	Results of all primary outcomes were reported.	Unclear
Luo JG 2018	Patients were divided into one of two groups using random	The person doing the randomization was blinded.	This article was a double-blind study	No information was provided.	The trial did not report any	Results of all primary	Unclear

	number table method				dropouts.	outcomes were reported.	
Ma YL 2011	The trial only reported "randomized"	No information was provided.	No information was provided.	No information was provided.	The trial did not report any dropouts.	Results of all primary outcomes were reported.	Unclear
Pan J 2016	Patients were randomly assigned to one of two treatment groups	No information was provided.	No information was provided.	No information was provided.	The trial did not report any dropouts.	Results of all primary outcomes were reported.	Unclear
Qi MG 2016	The trial only reported "randomized"	No information was provided.	No information was provided.	No information was provided.	The trial did not report any dropouts.	Results of all primary outcomes were reported.	Unclear
Qiu FP 2016	Patients were randomly assigned to one of two treatment groups	Allocation concealment was performed by enclosing assignments in sequentially numbered envelopes.	Both the patients and treating physicians were blinded to patient treatment assignments.	No information was provided.	The trial did not report any dropouts.	Results of all primary outcomes were reported.	Unclear
Shen SM 2012	Patients were randomly assigned to one of two treatment groups	No information was provided.	No information was provided.	No information was provided.	The trial did not report any dropouts.	Results of all primary outcomes were reported.	Unclear
Shen XY 2018	The trial only reported "randomized"	No information was provided.	No information was provided.	No information was provided.	The trial did not report any dropouts.	Results of all primary outcomes were reported.	Unclear
Shi GC 2014	Patients were randomly assigned to one of two treatment groups	No information was provided.	No information was provided.	No information was provided.	The trial did not report any	Results of all primary	Unclear

					dropouts.	outcomes were reported.	
Tang W 2017	Patients were divided into one of two groups using random number table method	The author who performed the randomization did not participate in the enrolment and allocation of treatment to the participants and concealment was done using sequentially labeled sealed envelopes containing the specified intervention.	This article was a double-blind study	Data were collected in a paper case report form and then entered into a database.	The trial did not report any dropouts.	Results of all primary outcomes were reported.	Unclear
Wang FX 2009	Patients were divided into one of two groups using random number table method	The person doing the randomization was blinded.	No information was provided.	Data were collected in a paper case report form and entered into a database.	The trial did not report any dropouts.	Results of all primary outcomes were reported.	Unclear
Wang NN 2012	The trial only reported “randomized”	No information was provided.	No information was provided.	No information was provided.	The trial did not report any dropouts.	Results of all primary outcomes were reported.	Unclear
Wang SY 2009	Patients were randomly assigned to one of two treatment groups	No information was provided.	No information was provided.	No information was provided.	The trial did not report any dropouts.	Results of all primary outcomes were reported.	Unclear
Wang T 2019	The trial only reported “randomized”	No information was provided.	No information was provided.	No information was provided.	The trial did not report any dropouts.	Results of all primary outcomes were reported.	Unclear

Wang XC 2010	Patients were randomly assigned to one of two treatment groups	No information was provided.	No information was provided.	No information was provided.	The trial did not report any dropouts.	Results of all primary outcomes were reported.	Unclear
Wang YH 2008	The trial only reported "randomized"	No information was provided.	No information was provided.	No information was provided.	The trial did not report any dropouts.	Results of all primary outcomes were reported.	Unclear
Wei XF 2018	The trial used a random number–producing algorithm in central computer systems for simple randomization.	Allocation concealment was done by sealed sequentially numbered opaque envelopes.	The patients were masked in regards to active versus placebo assignment.	Data were collected in a paper case report form and entered into a database.	The trial did not report any dropouts.	Results of all primary outcomes were reported.	Unclear
Wei SJ 2010	The trial only reported "randomized"	No information was provided.	No information was provided.	No information was provided.	The trial did not report any dropouts.	Results of all primary outcomes were reported.	Unclear
Wu P 2021	Patients were divided into one of two groups using random number table method	An analyst from a distant site, with no clinical involvement in the trial, kept randomization lists concealed.	This article was a double-blind study	No information was provided.	The trial did not report any dropouts.	Results of all primary outcomes were reported.	Unclear
Wu QF 2016	Patients were randomly assigned to one of two treatment groups	No information was provided.	No information was provided.	No information was provided.	The trial did not report any dropouts.	Results of all primary outcomes were reported.	Unclear
Wu L 2014	Patients were divided into one of two groups using random number table method	The person doing the randomization was blinded.	This article was a double-blind study.	Data were collected in a paper case report form and entered into a database.	The trial did not report any dropouts.	Results of all primary outcomes were reported.	Unclear

Xiao ZZ 2010	Patients were randomly assigned to one of two treatment groups	No information was provided.	No information was provided.	No information was provided.	The trial did not report any dropouts.	Results of all primary outcomes were reported.	Unclear
Xu L 2015	The trial only reported "randomized"	No information was provided.	No information was provided.	No information was provided.	The trial did not report any dropouts.	Results of all primary outcomes were reported.	Unclear
Yang CH 2013	Patients were randomly assigned to one of two treatment groups	No information was provided.	No information was provided.	No information was provided.	The trial did not report any dropouts.	Results of all primary outcomes were reported.	Unclear
Yang G 2016	Patients were divided into one of two groups using random number table method	Allocation concealment was done by sealed sequentially numbered opaque envelopes.	Both the patients, treating physicians, and individuals were blinded to patient treatment assignments.	No information was provided.	The trial did not report any dropouts.	Results of all primary outcomes were reported.	Unclear
Yang WQ 2020	The trial only reported "randomized"	No information was provided.	No information was provided.	No information was provided.	The trial did not report any dropouts.	Results of all primary outcomes were reported.	Unclear
Ye FL 2016	Patients were divided into one of two groups using random number table method	The subject numbers were assigned sequentially as each subject entered the study.	No information was provided.	Data were collected in a paper case report form and entered into a database. Data assessors were blinded to treatment allocation.	The trial did not report any dropouts.	Results of all primary outcomes were reported.	Unclear
Ye JB 2012	The trial only reported "randomized"	No information was provided.	No information was provided.	No information was provided.	The trial did not report any dropouts.	Results of all primary outcomes	Unclear

						were reported.	
Yu HT 2013	Patients were randomly assigned to one of two treatment groups	No information was provided.	No information was provided.	No information was provided.	The trial did not report any dropouts.	Results of all primary outcomes were reported.	Unclear
Yun P 2013	The trial only reported "randomized"	No information was provided.	No information was provided.	No information was provided.	Five cases were withdrawn from the trial after losing follow-up	Results of all primary outcomes were reported.	Unclear
Zhang C 2014	Patients were randomly assigned to one of two treatment groups	Allocation concealment was done by sealed sequentially numbered opaque envelopes.	The patients were masked in regards to active versus placebo assignment.	No information was provided.	Three cases were withdrawn from the trial after losing follow-up	Results of all primary outcomes were reported.	Unclear
Zhou J 2012	The trial only reported "randomized"	No information was provided.	No information was provided.	No information was provided.	The trial did not report any dropouts.	Results of all primary outcomes were reported.	Unclear
Zhou XJ 2016	The trial only reported "randomized"	No information was provided.	No information was provided.	No information was provided.	The trial did not report any dropouts.	Results of all primary outcomes were reported.	Unclear
Zhu HY 2015	The trial used a random number–producing algorithm in central computer systems for simple randomization.	The author who performed the randomization did not participate in the enrolment	The patients were masked in regards to active versus placebo assignment	Data were collected in a paper case report form and entered into a database.	The trial did not report any dropouts.	Results of all primary outcomes were reported.	Unclear

Cai JY 2010	Patients were divided into one of two groups using random number table method	Allocation concealment was performed by enclosing assignments in sequentially numbered, opaque-closed envelopes.	This article was a double-blind study.	No information was provided.	The trial did not report any dropouts.	Results of all primary outcomes were reported.	Unclear
Cai XY 2010	The trial only reported "randomized"	No information was provided.	Using single blind randomization	No information was provided.	The trial did not report any dropouts.	Results of all primary outcomes were reported.	Unclear
Cao XC 2015	Patients were divided into one of two groups using random number table method	The person doing the randomization was blinded.	The recruiting and treating doctors, as well as the patients, were blinded on the type of treatment throughout the medication period.	Data were collected in a paper case report form and entered into a database.	The trial did not report any dropouts.	Results of all primary outcomes were reported.	Unclear
Cao XX 2017	Patients were randomly assigned to one of two treatment groups	No information was provided.	No information was provided.	No information was provided.	The trial did not report any dropouts.	Results of all primary outcomes were reported.	Unclear
Cao YX 2019	The trial only reported "randomized"	No information was provided.	No information was provided.	No information was provided.	The trial did not report any dropouts.	Results of all primary outcomes were reported.	Unclear
Chen F 2010	The trial only reported "randomized"	No information was provided.	No information was provided.	No information was provided.	The trial did not report any dropouts.	Results of all primary outcomes were reported.	Unclear
Chen QS 2016	Patients were randomly assigned to one of two treatment groups	The author who performed the randomization did not participate in the enrolment	This article was a double-blind study.	Data were collected in a paper case report form and entered into a database. Data	The trial did not report any dropouts.	Results of all primary outcomes were	Unclear

				assessors were blinded to treatment allocation.		reported.	
Chen QJ 2017	The trial only reported "randomized"	No information was provided.	No information was provided.	No information was provided.	The trial did not report any dropouts.	Results of all primary outcomes were reported.	Unclear
Chen SS 2020	Patients were randomly assigned to one of two treatment groups	No information was provided.	No information was provided.	No information was provided.	The trial did not report any dropouts.	Results of all primary outcomes were reported.	Unclear
Chen SW 2018	Patients were randomly assigned to one of two treatment groups	The author who performed the randomization did not participate in the enrolment	Both the patients, treating physicians, and individuals were blinded to patient treatment assignments.	No information was provided.	The trial did not report any dropouts.	Results of all primary outcomes were reported.	Unclear
Chen Y 2020	The trial only reported "randomized"	No information was provided.	No information was provided.	No information was provided.	The trial did not report any dropouts.	Results of all primary outcomes were reported.	Unclear
Chen Y 2015	Patients were randomly assigned to one of two treatment groups	No information was provided.	No information was provided.	No information was provided.	The trial did not report any dropouts.	Results of all primary outcomes were reported.	Unclear
Chi PW 2012	Patients were randomly assigned to one of two treatment groups	No information was provided.	No information was provided.	No information was provided.	The trial did not report any dropouts.	Results of all primary outcomes were reported.	Unclear

Dai XM 2012	Random grouping method is not mentioned in the trial	The subject numbers were assigned sequentially as each subject entered the study.	No information was provided.	Data were collected in a paper case report form and entered into a database.	The trial did not report any dropouts.	Results of all primary outcomes were reported.	Unclear
Deng SY 2014	Patients were randomly assigned to one of two treatment groups	No information was provided.	No information was provided.	No information was provided.	The trial did not report any dropouts.	Results of all primary outcomes were reported.	Unclear
Deng SY 2016	Patients were randomly assigned to one of two treatment groups	The person doing the randomization was blinded.	This article was a double-blind study.	No information was provided.	The trial did not report any dropouts.	Results of all primary outcomes were reported.	Unclear
Dou JF 2006	Patients were randomly assigned to one of two treatment groups	No information was provided.	No information was provided.	No information was provided.	The trial did not report any dropouts.	Results of all primary outcomes were reported.	Unclear
Fan YT 2015	The trial only reported “randomized”	No information was provided.	No information was provided.	No information was provided.	The trial did not report any dropouts.	Results of all primary outcomes were reported.	Unclear
Feng ZL 2017	Patients were randomly assigned to one of two treatment groups	The person doing the randomization was blinded.	This article was a double-blind study.	No information was provided.	The trial did not report any dropouts.	Results of all primary outcomes were reported.	Unclear
Gao X 2018	Patients were randomly assigned to one of two treatment groups	No information was provided.	No information was provided.	No information was provided.	The trial did not report any dropouts.	Results of all primary outcomes were reported.	Unclear

Ge QR 2011	Patients were divided into one of two groups using random number table method	Allocation concealment was done by sealed sequentially numbered opaque envelopes. They were consecutively numbered and bottles were provided to the patients according to the number allocated	The patients were masked in regards to treatment versus placebo assignment, but the physicians were not for safety reasons and because the end points were objective and measured centrally by a lab blinded to patient designation.	Data were collected in a paper case report form and entered into a database. Data assessors were blinded to treatment allocation.	The trial did not report any dropouts.	Results of all primary outcomes were reported.	Unclear
Gu RY 2018	The trial only reported "randomized"	No information was provided.	No information was provided.	No information was provided.	The trial did not report any dropouts.	Results of all primary outcomes were reported.	Unclear
Guan YH 2020	Patients were grouping according to medication regimen	No information was provided.	No information was provided.	No information was provided.	The trial did not report any dropouts.	Results of all primary outcomes were reported.	Unclear
Guan CA 2021	Using random number table	The author who performed the randomization did not participate in the enrolment	This article was a double-blind study.	Data were collected in a paper case report form and entered into a database.	The trial did not report any dropouts.	Results of all primary outcomes were reported.	Unclear
Guo G 2015	Patients were randomly assigned to one of two treatment groups	No information was provided.	No information was provided.	No information was provided.	The trial did not report any dropouts.	Results of all primary outcomes were reported.	Unclear
Guo T 2016	Patients were grouping according to medication regimen	No information was provided.	No information was provided.	No information was provided.	The trial did not report any dropouts.	Results of all primary outcomes were	Unclear

reported.

He P 2012	Using random number table	The person doing the randomization was blinded.	This article was a double-blind study.	No information was provided.	The trial did not report any dropouts.	Results of all primary outcomes were reported.	Unclear
He XH 2012	Patients were randomly assigned to one of two treatment groups	No information was provided.	No information was provided.	No information was provided.	The trial did not report any dropouts.	Results of all primary outcomes were reported.	Unclear
He YY 2021	Patients were divided into one of two groups using random number table method	The subject numbers were assigned sequentially as each subject entered the study.	This article was a double-blind study.	Data were collected in a paper case report form and entered into a database.	The trial did not report any dropouts.	Results of all primary outcomes were reported.	Unclear
He YN 2010	Patients were randomly assigned to one of two treatment groups	No information was provided.	No information was provided.	No information was provided.	The trial did not report any dropouts.	Results of all primary outcomes were reported.	Unclear
Hu QS 2007	Patients were semi-randomly divided into one of two treatment groups	The person doing the randomization was blinded.	Using single blind semi random grouping	No information was provided.	The trial did not report any dropouts.	Results of all primary outcomes were reported.	Unclear
Hu WF 2012	Patients were randomly assigned to one of two treatment groups	No information was provided.	No information was provided.	No information was provided.	The trial did not report any dropouts.	Results of all primary outcomes were reported.	Unclear

Hu XJ 2019	Patients were divided into one of two groups using random number table method	The author who performed the randomization did not participate in the enrolment	Both the patients, treating physicians, and individuals were blinded to patient treatment assignments.	Data were collected in a paper case report form and entered into a database.	The trial did not report any dropouts.	Results of all primary outcomes were reported.	Unclear
Hu YG 2016	Patients were randomly assigned to one of two treatment groups	No information was provided.	No information was provided.	No information was provided.	The trial did not report any dropouts.	Results of all primary outcomes were reported.	Unclear
Hu Y 2016	Patients were divided into one of two groups using random number table method	Allocation concealment was performed by enclosing assignments in sequentially numbered, opaque-closed envelopes.	This article was a double-blind study.	No information was provided.	The trial did not report any dropouts.	Results of all primary outcomes were reported.	Unclear
Huang T 2010	Patients were divided into one of two groups using random number table method	The person doing the randomization was blinded.	Both the patients, treating physicians, and individuals were blinded to patient treatment assignments.	No information was provided.	The trial did not report any dropouts.	Results of all primary outcomes were reported.	Unclear
Jia ZW 2015	Patients were randomly assigned to one of two treatment groups	No information was provided.	No information was provided.	No information was provided.	The trial did not report any dropouts.	Results of all primary outcomes were reported.	Unclear
Xie WY 2019	Patients were grouping according to medication regimen	No information was provided.	No information was provided.	No information was provided.	The trial did not report any dropouts.	Results of all primary outcomes were reported.	Unclear
Lan YL 2021	Patients were divided into one of two groups using random number table method	Allocation concealment was done by sealed sequentially numbered opaque envelopes.	The patients were masked in regards to active versus placebo	No information was provided.	The trial did not report any dropouts.	Results of all primary outcomes were reported.	Unclear

Li HJ 2013	Patients were randomly assigned to one of two treatment groups	No information was provided.	No information was provided.	No information was provided.	The trial did not report any dropouts.	Results of all primary outcomes were reported.	Unclear
Li BY 2015	Patients were divided into one of two groups using random number table method	The subject numbers were assigned sequentially as each subject entered the study.	This article was a double-blind study.	No information was provided.	The trial did not report any dropouts.	Results of all primary outcomes were reported.	Unclear
Li HS 2017	Using random sequential comprehensive equilibrium method	The author who performed the randomization did not participate in the enrolment	The recruiting and treating doctors, as well as the patients, were blinded on the type of treatment throughout the medication period.	No information was provided.	The trial did not report any dropouts.	Results of all primary outcomes were reported.	Unclear
Li HN 2022	Patients were grouping according to medication regimen	No information was provided.	No information was provided.	No information was provided.	The trial did not report any dropouts.	Results of all primary outcomes were reported.	Unclear
Li Q 2020	Dividing into groups according to the number of people	No information was provided.	No information was provided.	No information was provided.	The trial did not report any dropouts.	Results of all primary outcomes were reported.	Unclear
Li Q 2021	Patients were randomly assigned to one of two treatment groups	No information was provided.	No information was provided.	No information was provided.	The trial did not report any dropouts.	Results of all primary outcomes were reported.	Unclear
Li QH 2010	The trial used a random number–producing algorithm in central computer systems for simple	Allocation concealment was performed by enclosing assignments in sequentially numbered, opaque-closed	This article was a double-blind study.	Data were collected in a paper case report form and entered into a database.	The trial did not report any dropouts.	Results of all primary outcomes were	Unclear

	randomization.	envelopes.				reported.	
Li YN 2013	Patients were randomly assigned to one of two treatment groups	No information was provided.	No information was provided.	No information was provided.	The trial did not report any dropouts.	Results of all primary outcomes were reported.	Unclear
Liang YP 2014	Patients were randomly assigned to one of two treatment groups	The subject numbers were assigned sequentially as each subject entered the study.	This article was a double-blind study.	No information was provided.	The trial did not report any dropouts.	Results of all primary outcomes were reported.	Unclear
Lin M 2013	The trial only reported "randomized"	No information was provided.	No information was provided.	No information was provided.	The trial did not report any dropouts.	Results of all primary outcomes were reported.	Unclear
Lin M 2011	Patients were semi-randomly divided into one of two treatment groups	No information was provided.	Using single blind random grouping	No information was provided.	The trial did not report any dropouts.	Results of all primary outcomes were reported.	Unclear
Lin ZN 2016	Patients were randomly assigned to one of two treatment groups	No information was provided.	No information was provided.	No information was provided.	The trial did not report any dropouts.	Results of all primary outcomes were reported.	Unclear
Liu CP 2011	Patients were divided into one of two groups using random number table method	The person doing the randomization was blinded.	Both the patients, treating physicians, and individuals were blinded to patient treatment assignments.	No information was provided.	The trial did not report any dropouts.	Results of all primary outcomes were reported.	Unclear

Liu L 2021	Patients were randomly assigned to one of two treatment groups	The subject numbers were assigned sequentially as each subject entered the study.	This article was a double-blind study.	No information was provided.	The trial did not report any dropouts.	Results of all primary outcomes were reported.	Unclear
Liu L 2012	Patients were randomly assigned to one of two treatment groups	No information was provided.	No information was provided.	No information was provided.	The trial did not report any dropouts.	Results of all primary outcomes were reported.	Unclear
Liu XQ 2012	Patients were randomly assigned to one of two treatment groups	No information was provided.	No information was provided.	No information was provided.	The trial did not report any dropouts.	Results of all primary outcomes were reported.	Unclear
Liu XD 2011	Patients were randomly assigned to one of two treatment groups	The person doing the randomization was blinded.	This article was a double-blind study.	No information was provided.	The trial did not report any dropouts.	Results of all primary outcomes were reported.	Unclear
Liu YP 2018	Patients were randomly assigned to one of two treatment groups	The author who performed the randomization did not participate in the enrolment	Both the patients, treating physicians, and individuals were blinded to patient treatment assignments.	No information was provided.	The trial did not report any dropouts.	Results of all primary outcomes were reported.	Unclear
Liu YH 2011	Random grouping method is not mentioned in the trial	No information was provided.	No information was provided.	No information was provided.	The trial did not report any dropouts.	Results of all primary outcomes were reported.	Unclear
Lou PH 2010	Patients were randomly assigned to one of two treatment groups	No information was provided.	No information was provided.	No information was provided.	The trial did not report any dropouts.	Results of all primary outcomes were reported.	Unclear

Luo JJ 2021	The trial used a random number–producing algorithm in central computer systems for simple randomization.	Allocation concealment was done by sealed sequentially numbered opaque envelopes.	The recruiting and treating doctors, as well as the patients, were blinded on the type of treatment	No information was provided.	The trial did not report any dropouts.	Results of all primary outcomes were reported.	Unclear
Lv F 2012	Patients were randomly assigned to one of two treatment groups	No information was provided.	No information was provided.	No information was provided.	The trial did not report any dropouts.	Results of all primary outcomes were reported.	Unclear
Lv MF 2015	Random grouping method is not mentioned in the trial	No information was provided.	No information was provided.	No information was provided.	The trial did not report any dropouts.	Results of all primary outcomes were reported.	Unclear
Nie XY 2018	Patients were divided into one of two groups using random number table method	Allocation concealment was performed by enclosing assignments in sequentially numbered, opaque-closed envelopes.	Both the patients, treating physicians, and individuals were blinded to patient treatment assignments.	No information was provided.	The trial did not report any dropouts.	Results of all primary outcomes were reported.	Unclear
Ou YL 2015	Patients were randomly assigned to one of two treatment groups	No information was provided.	No information was provided.	No information was provided.	The trial did not report any dropouts.	Results of all primary outcomes were reported.	Unclear
Pan CY 2016	Patients were randomly assigned to one of two treatment groups	No information was provided.	No information was provided.	No information was provided.	The trial did not report any dropouts.	Results of all primary outcomes were reported.	Unclear
Qi JY 2018	Patients were randomly assigned to one of two treatment groups	The person doing the randomization was blinded.	This article was a double-blind study.	No information was provided.	The trial did not report any dropouts.	Results of all primary outcomes were reported.	Unclear

Qiao AM 2013	Patients were randomly assigned to one of two treatment groups	No information was provided.	No information was provided.	No information was provided.	The trial did not report any dropouts.	Results of all primary outcomes were reported.	Unclear
Ren X 2020	Patients were divided into one of two groups using random number table method	The author who performed the randomization did not participate in the enrolment and allocation of treatment to the participants and concealment was done using sequentially labeled sealed envelopes	This article was a double-blind study.	No information was provided.	The trial did not report any dropouts.	Results of all primary outcomes were reported.	Unclear
Shen ML 2015	Random according to the order of admission	No information was provided.	No information was provided.	No information was provided.	The trial did not report any dropouts.	Results of all primary outcomes were reported.	Unclear
Su JF 2019	Patients were divided into one of two groups using random number table method	The subject numbers were assigned sequentially as each subject entered the study.	The recruiting and treating doctors, as well as the patients, were blinded on the type of treatment	No information was provided.	The trial did not report any dropouts.	Results of all primary outcomes were reported.	Unclear
Sun FY 2021	Patients were divided into one of two groups using random number table method	The person doing the randomization was blinded.	This article was a double-blind study	No information was provided.	The trial did not report any dropouts.	Results of all primary outcomes were reported.	Unclear
Sun JY 2015	Patients were randomly assigned to one of two treatment groups	No information was provided.	No information was provided.	No information was provided.	The trial did not report any dropouts.	Results of all primary outcomes were reported.	Unclear
Sun SR 2014	Patients were randomly assigned to one of two treatment groups	No information was provided.	No information was provided.	No information was provided.	The trial did not report any dropouts.	Results of all primary outcomes	Unclear

						were reported.	
Tang YZ 2011	Patients were randomly assigned to one of two treatment groups	No information was provided.	No information was provided.	No information was provided.	The trial did not report any dropouts.	Results of all primary outcomes were reported.	Unclear
Tian XY 2019	Patients were divided into one of two groups using random number table method	Allocation concealment was done by sealed sequentially numbered opaque envelopes.	This article was a double-blind study.	Data were collected in a paper case report form and entered into a database and data assessors were blinded to treatment allocation.	The trial did not report any dropouts.	Results of all primary outcomes were reported.	Unclear
Wang AY 2020	Patients were randomly assigned to one of two treatment groups	No information was provided.	No information was provided.	No information was provided.	The trial did not report any dropouts.	Results of all primary outcomes were reported.	Unclear
Wang G 2006	Patients were divided into one of two groups using random number table method	Allocation concealment was performed by enclosing assignments in sequentially numbered, opaque-closed envelopes.	Both the patients, treating physicians, and individuals were blinded to patient treatment assignments.	No information was provided.	The trial did not report any dropouts.	Results of all primary outcomes were reported.	Unclear
Wang JM 2015	Random according to the order of admission	No information was provided.	No information was provided.	No information was provided.	The trial did not report any dropouts.	Results of all primary outcomes were reported.	Unclear
Wang KY 2020	Random allocation by drawing lots	No information was provided.	No information was provided.	No information was provided.	The trial did not report any dropouts.	Results of all primary outcomes were reported.	Unclear

Wang LW 2018	Dividing into groups with random envelope method	The subject numbers were assigned sequentially as each subject entered the study.	This article was a double-blind study.	No information was provided.	The trial did not report any dropouts.	Results of all primary outcomes were reported.	Unclear
Wang XH 2015	Double blind grouping	No information was provided.	No information was provided.	No information was provided.	The trial did not report any dropouts.	Results of all primary outcomes were reported.	Unclear
Wu N 2013	Patients were randomly assigned to one of two treatment groups	No information was provided.	No information was provided.	No information was provided.	The trial did not report any dropouts.	Results of all primary outcomes were reported.	Unclear
Xiao XY 2011	Grouping by visit order	No information was provided.	No information was provided.	No information was provided.	The trial did not report any dropouts.	Results of all primary outcomes were reported.	Unclear
Xiao Y 2021	Patients were divided into one of two groups using random number table method	An analyst from a distant site, with no clinical involvement in the trial, kept randomization lists concealed.	The patients were masked in regards to active versus placebo assignment.	No information was provided.	The trial did not report any dropouts.	Results of all primary outcomes were reported.	Unclear
Xu JZ 2016	Patients were grouping according to medication regimen	No information was provided.	No information was provided.	No information was provided.	The trial did not report any dropouts.	Results of all primary outcomes were reported.	Unclear
Xu WM 2013	Patients were randomly assigned to one of two treatment groups	No information was provided.	No information was provided.	No information was provided.	The trial did not report any dropouts.	Results of all primary outcomes were reported.	Unclear

Xu XM 2020	Random allocation by drawing lots	The person doing the randomization was blinded.	This article was a double-blind study.	Data were collected in a paper case report form and entered into a database and data assessors were blinded to treatment allocation.	The trial did not report any dropouts.	Results of all primary outcomes were reported.	Unclear
Yang P 2014	Using mechanical sampling method	The person doing the randomization was blinded.	This article was a double-blind study.	No information was provided.	The trial did not report any dropouts.	Results of all primary outcomes were reported.	Unclear
Yang T 2013	Patients were randomly assigned to one of two treatment groups	No information was provided.	No information was provided.	No information was provided.	The trial did not report any dropouts.	Results of all primary outcomes were reported.	Unclear
Yang XM 2012	Patients were randomly assigned to one of two treatment groups	No information was provided.	No information was provided.	No information was provided.	The trial did not report any dropouts.	Results of all primary outcomes were reported.	Unclear
Yu HJ 2022	Group randomly by tossing a coin	The author who performed the randomization did not participate in the enrolment and allocation of treatment to the participants and concealment was done using sequentially labeled sealed envelopes containing the specified intervention.	Both the patients, treating physicians, and individuals were blinded to patient treatment assignments.	No information was provided.	The trial did not report any dropouts.	Results of all primary outcomes were reported.	Unclear
Yu JW 2021	Patients were divided into one of two groups using random number table method	No information was provided.	No information was provided.	No information was provided.	The trial did not report any dropouts.	Results of all primary outcomes were	Unclear

						reported.	
Yuan JF 2019	Patients were randomly assigned to one of two treatment groups	No information was provided.	This article was a double-blind study.	No information was provided.	The trial did not report any dropouts.	Results of all primary outcomes were reported.	Unclear
Yuan YH 2017	Patients were randomly assigned to one of two treatment groups	The subject numbers were assigned sequentially as each subject entered the study.	The patients were masked in regards to active versus placebo assignment.	No information was provided.	The trial did not report any dropouts.	Results of all primary outcomes were reported.	Unclear
Zhang LF 2010	Random grouping method is not mentioned in the trial	No information was provided.	No information was provided.	No information was provided.	The trial did not report any dropouts.	Results of all primary outcomes were reported.	Unclear
Zhang M 2021	Random according to the order of admission	No information was provided.	No information was provided.	No information was provided.	The trial did not report any dropouts.	Results of all primary outcomes were reported.	Unclear
Zhang Q 2020	Random grouping according to the mantissa of the inpatient number	No information was provided.	No information was provided.	No information was provided.	The trial did not report any dropouts.	Results of all primary outcomes were reported.	Unclear
Zhang RX 2016	Patients were randomly assigned to one of two treatment groups	No information was provided.	No information was provided.	No information was provided.	The trial did not report any dropouts.	Results of all primary outcomes were reported.	Unclear

Zhang XD 2016	Patients were divided into one of two groups using random number table method	Allocation concealment was performed by enclosing assignments in sequentially numbered, opaque-closed envelopes.	The patients were masked in regards to active versus placebo assignment, but the physicians were not for safety reasons and because the end points were objective and measured centrally by a lab blinded to patient designation.	Data were collected in a paper case report form and entered into a database. Data assessors were blinded to treatment allocation.	The trial did not report any dropouts.	Results of all primary outcomes were reported.	Unclear
Zhang Y 2021	Patients were divided into one of two groups using random number table method	The author who performed the randomization did not participate in the enrolment	Both the patients, treating physicians, and individuals were blinded to patient treatment assignments.	No information was provided.	The trial did not report any dropouts.	Results of all primary outcomes were reported.	Unclear
Zhang YY 2019	Patients were randomly assigned to one of two treatment groups	No information was provided.	No information was provided.	No information was provided.	The trial did not report any dropouts.	Results of all primary outcomes were reported.	Unclear
Zhang Z 2019	Patients were randomly assigned to one of two treatment groups	No information was provided.	No information was provided.	No information was provided.	The trial did not report any dropouts.	Results of all primary outcomes were reported.	Unclear
Zhang ZY 2014	Patients were randomly assigned to one of two treatment groups	No information was provided.	No information was provided.	No information was provided.	The trial did not report any dropouts.	Results of all primary outcomes were reported.	Unclear
Zhao LP 2021	The trial used a random number–producing algorithm in central computer systems for simple randomization.	Allocation concealment was performed by enclosing assignments in sequentially numbered, opaque-closed envelopes.	The recruiting and treating doctors, as well as the patients, were blinded on the type of treatment throughout the	No information was provided.	The trial did not report any dropouts.	Results of all primary outcomes were reported.	Unclear

medication period.

Zheng JQ 2011	Random grouping method is not mentioned in the trial	No information was provided.	No information was provided.	No information was provided.	The trial did not report any dropouts.	Results of all primary outcomes were reported.	Unclear
Zhou GJ 2013	Patients were randomly assigned to one of two treatment groups	No information was provided.	No information was provided.	No information was provided.	The trial did not report any dropouts.	Results of all primary outcomes were reported.	Unclear
Zhou SQ 2015	Patients were randomly assigned to one of two treatment groups	No information was provided.	No information was provided.	No information was provided.	The trial did not report any dropouts.	Results of all primary outcomes were reported.	Unclear
Zhu HY 2020	Patients were divided into one of two groups using random number table method	The subject numbers were assigned sequentially as each subject entered the study.	This article was a double-blind study.	Data were collected in a paper case report form and entered into a database. Data assessors were blinded to treatment allocation.	The trial did not report any dropouts.	Results of all primary outcomes were reported.	Unclear

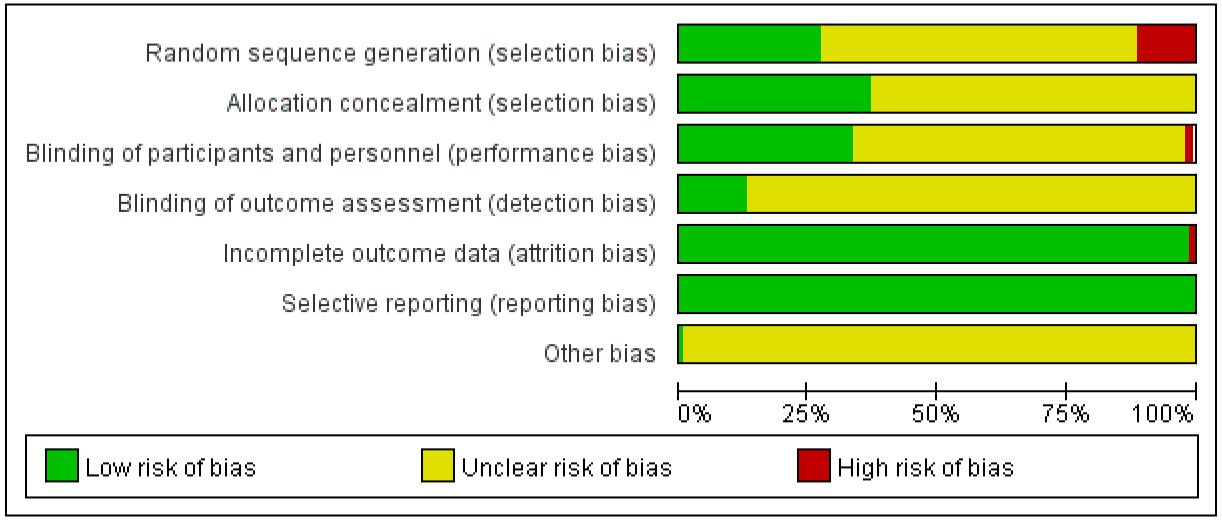

eFig.1 Risk of bias graph

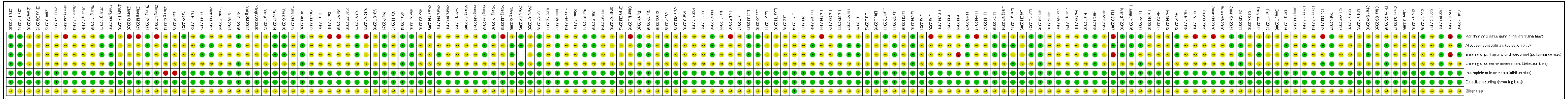

eFig.2 Risk of bias summary

Supplementary File 4: results of sensitivity analysis

1. Glycosylated Hemoglobin,Type A1c

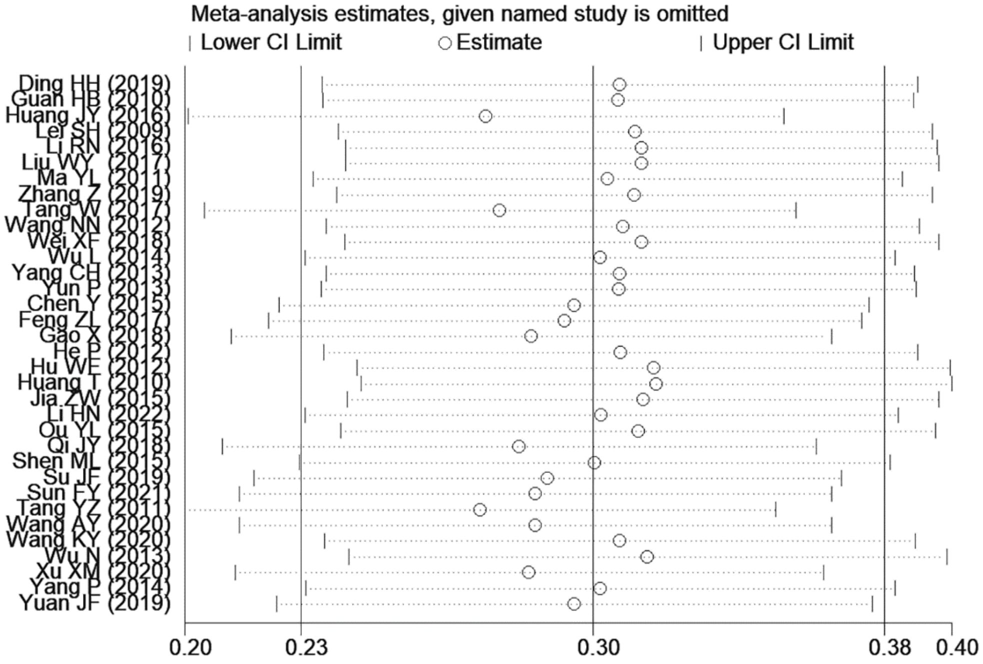

2. 24 hours urinary total protein

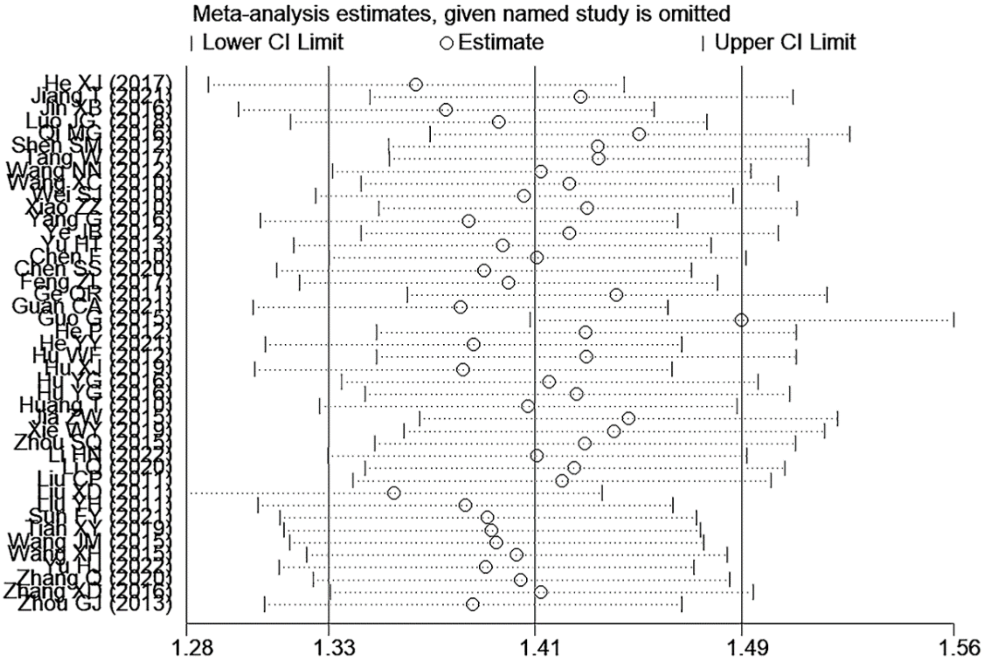

Supplementary File 5: Funnel plots

1. urinary albumin excretion rate

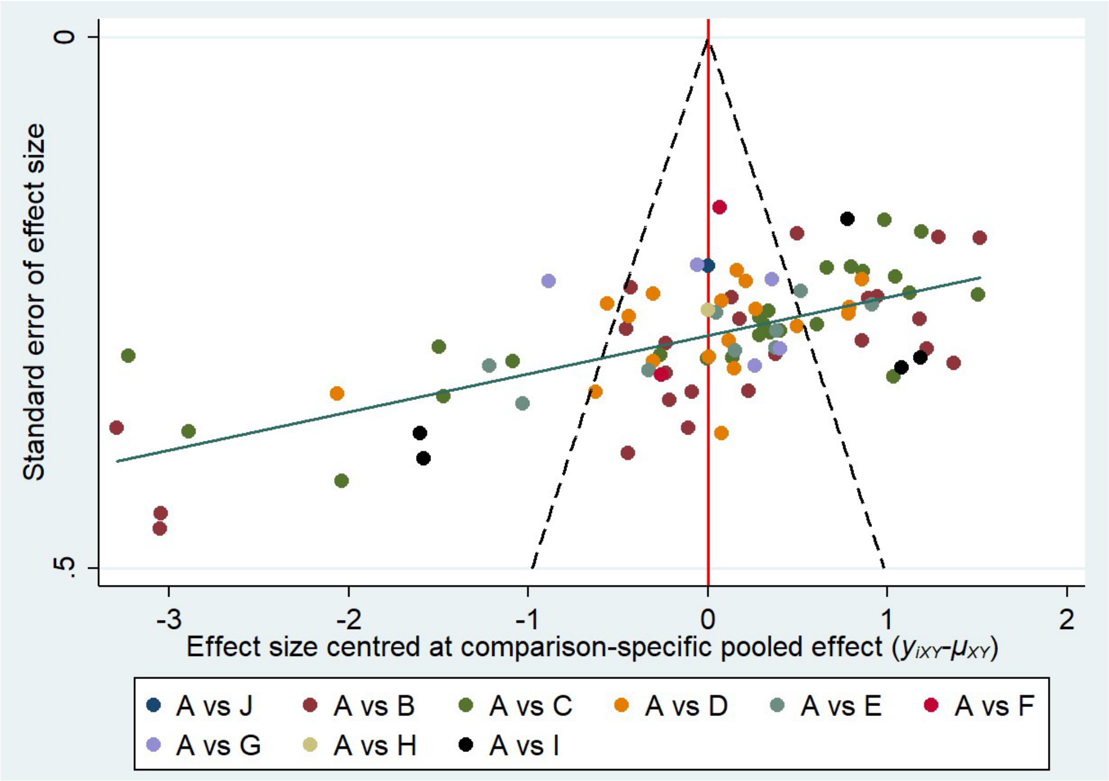

2. overall response rate

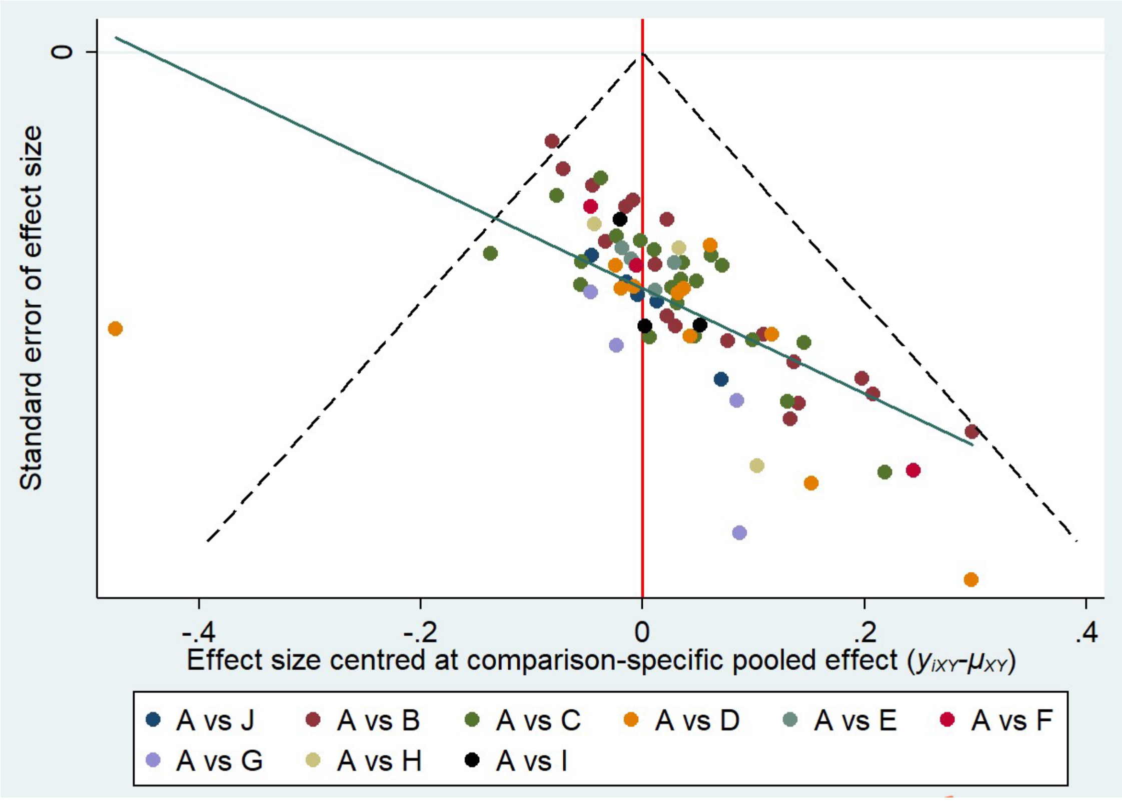

3. serum creatinine

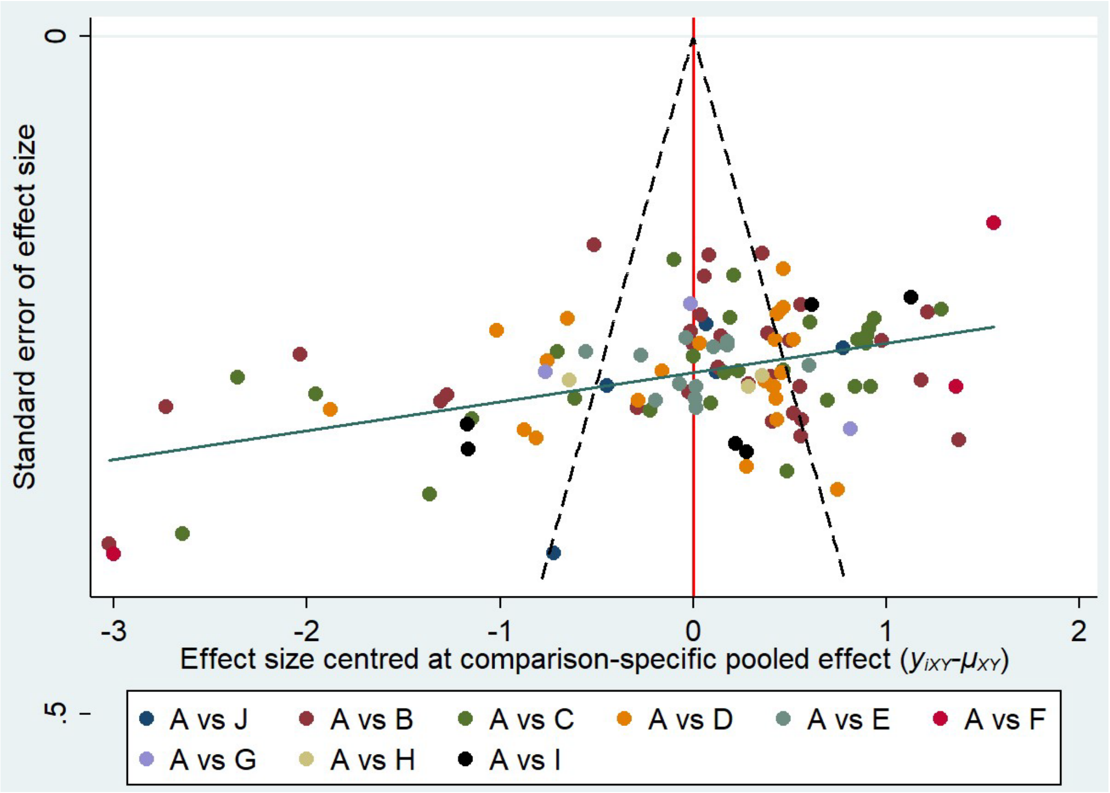

4. 24 hours urinary total protein

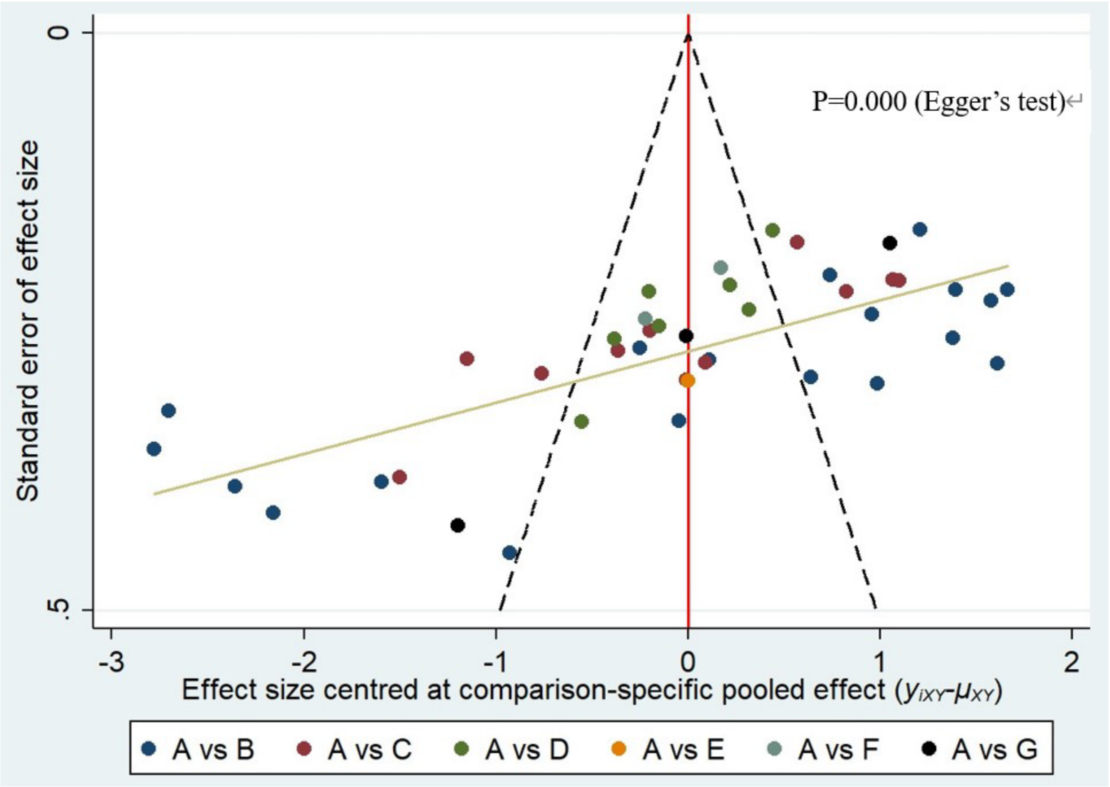

5. Total cholesterol

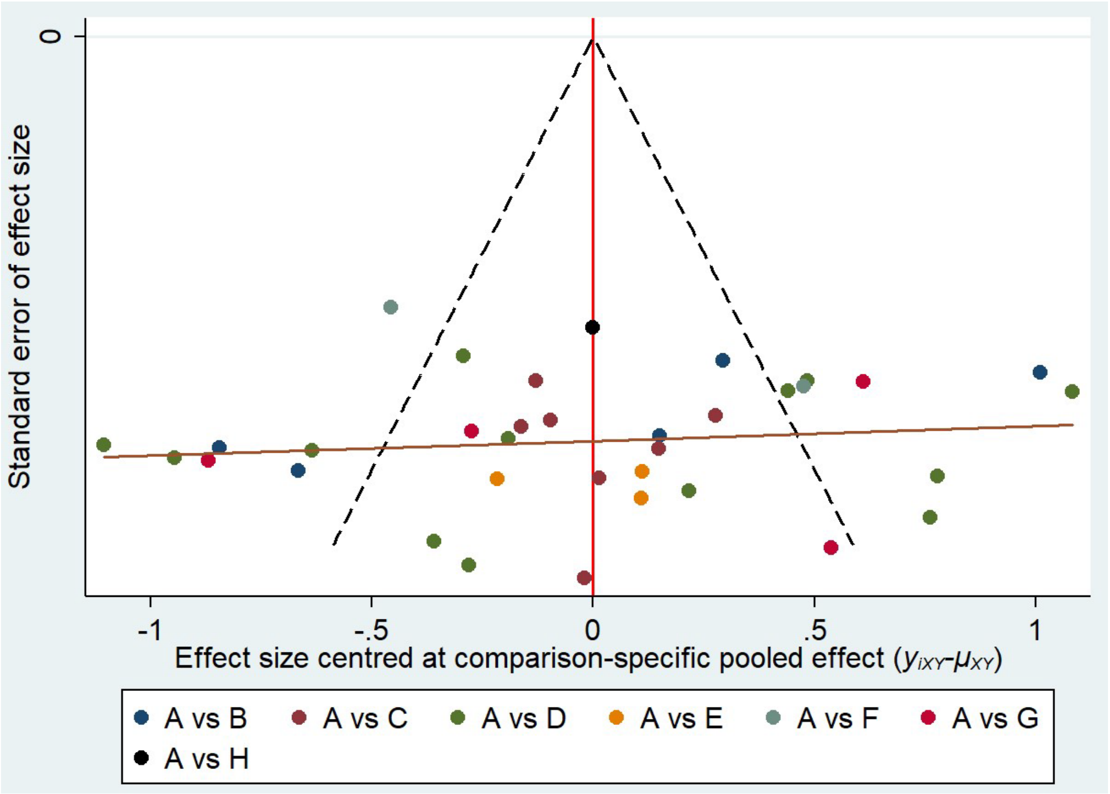

6. Triglyceride

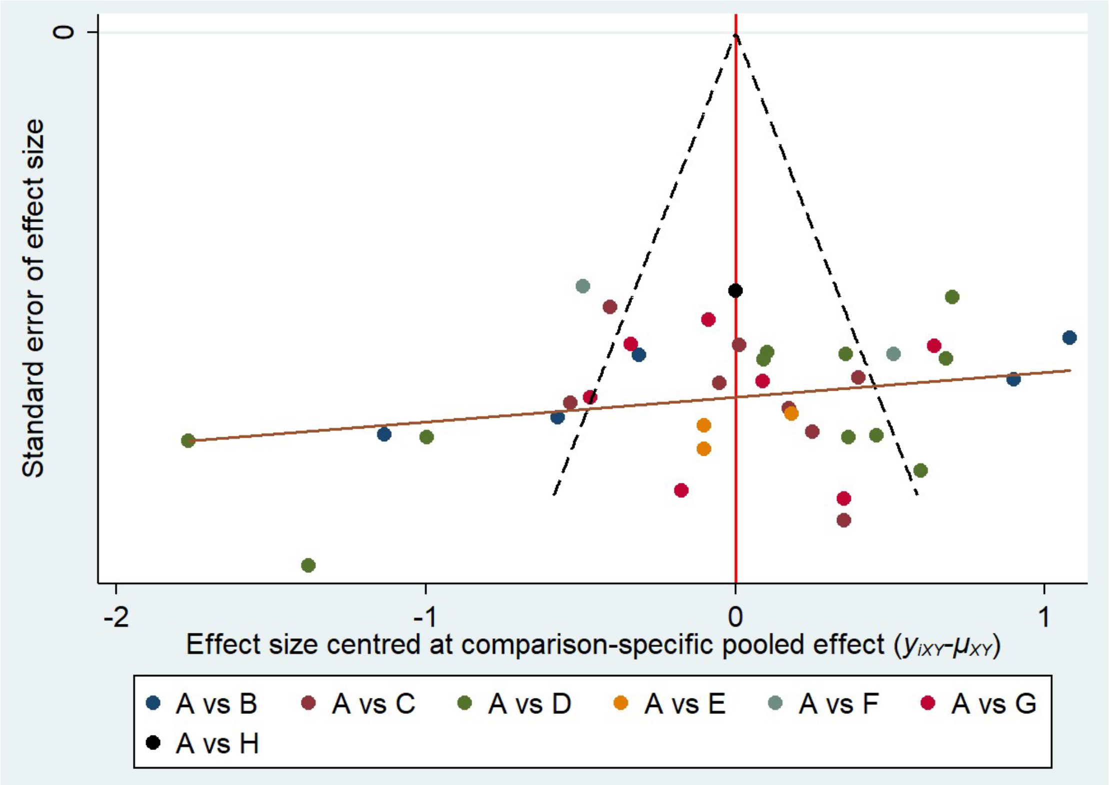

7. Glycosylated Hemoglobin,Type A1c

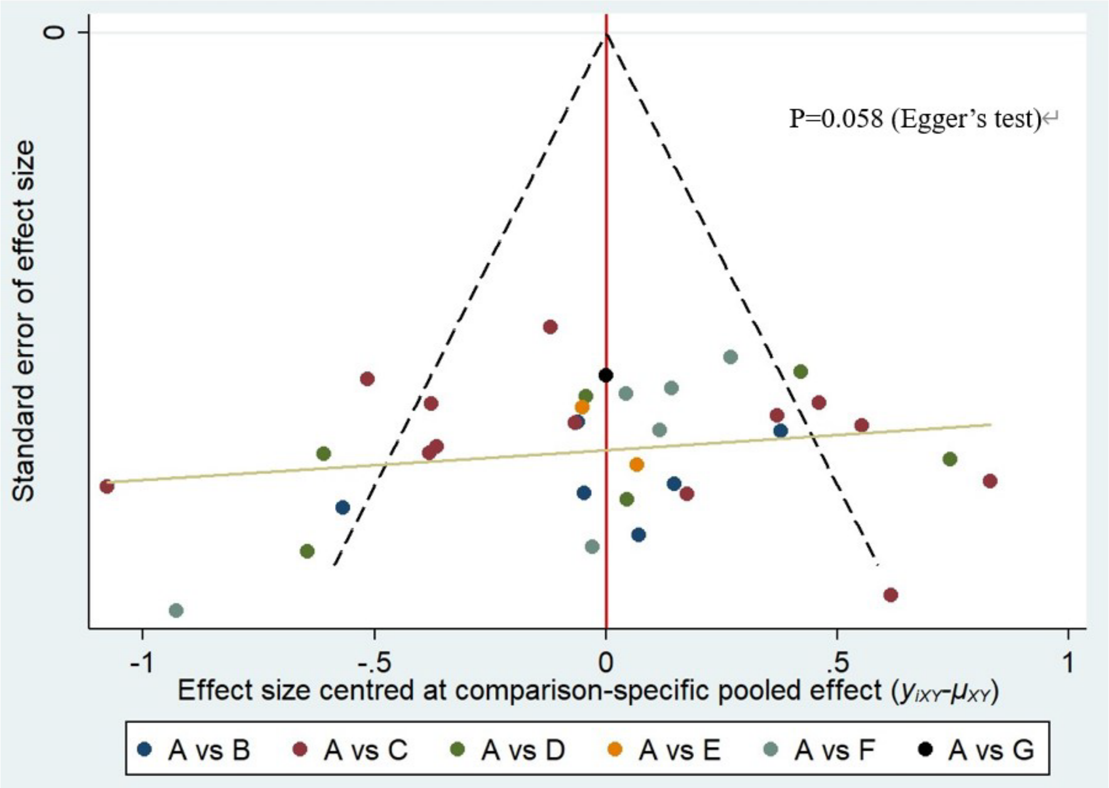

8. Adverse effects

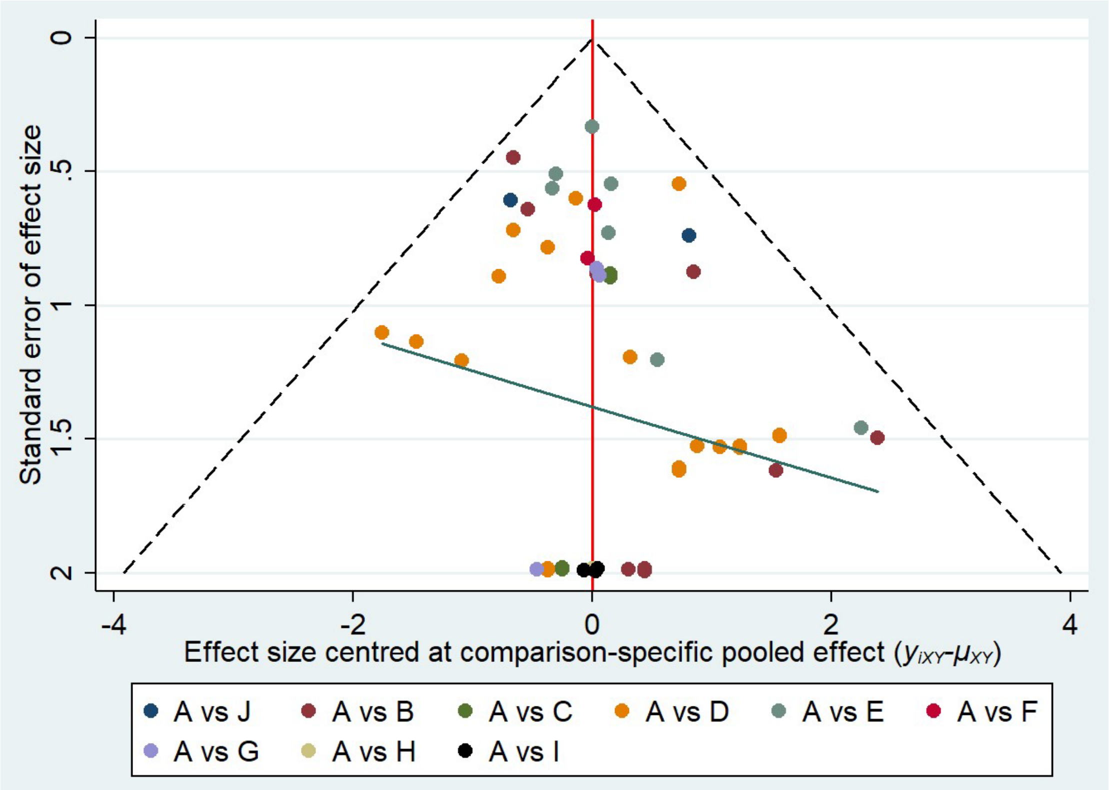

Notes: ACEI, angiotensin-converting enzyme inhibitors, ARB, angiotensin receptor blockers; BLC, Bailing Capsule; JSB, Jinshuibao Capsule; HKC, Huangkui Capsule; UCG, Uremic Clearance Granule; TG, Tripterygium glycosides; CXC, Compound Xueshuantong Capsule; SYKFT, Shenyan Kangfu Tablet; KLX, Keluoxin Capsule; SSNT, Shenshuaining Tablet;

Supplementary Table 1: The selection criteria with a "PICOS" structure

eTable 2. The selection criteria with a "PICOS" structure for the enrolled studies.

Items	Specific Criteria
Patients	Patients who have been diagnosed with diabetes mellitus and meet the diagnostic criteria (UAER of 20-200g/min or the urinary albumin-creatinine ratio of 30-300mg/24h) of early DKD were included.
Interventions/Comparisons	Interventions/Comparisons included BLC, HKC, JSB, UGG, TG, CXC, SYKFT, KLX, SSNT and ACEI/ARB.
Outcomes	The primary outcomes include UAER, ORR, Scr and 24h UTP. Secondary outcomes include TC, Trig, HbA1c and AEs.
Study designs	Studies were randomized controlled trials (RCTs)

DKD, diabetic kidney disease; RCTs, randomized controlled trials; BLC, Bailing Capsule; JSB, Jinshuibao Capsule; HKC, Huangkui Capsule; UCG, Uremic Clearance Granule; TG, Tripterygium glycosides; CXC, Compound Xueshuantong Capsule; SYKFT, Shenyan Kangfu Tablet; KLX, Keluoxin Capsule; SSNT, Shenshuaining Tablet; ACEI, angiotensin-converting enzyme inhibitor; ARB, angiotensin receptor blocker; Scr, serum creatinine; UAER, urinary albumin excretion rate; ORR, overall response rate; HbA1c, glycosylated hemoglobin, Type A1c; 24h UTP, 24 hours urinary total protein; TC, total cholesterol; Trig, triglyceride; AEs, adverse effects.

Supplementary Table 2: Heterogeneity of outcomes

eTable 3. Heterogeneity of outcomes

Outcomes	Dbar	Data points	Pd	DIC	I2
UAER	181.705	180	169.647	351.352	1%
ORR	145.018	148	74.235	219.253	0%
Scr	225.111	228	211.038	436.149	0%
24h UTP	88.035	86	84.702	172.737	3%
HbA1c	70.901	68	59.835	130.736	6%
TC	69.773	70	64.711	134.484	1%
Trig	76.77	76	69.415	146.185	2%

UAER, urinary albumin excretion rate; ORR, overall response rate; Scr, serum creatinine; 24h UTP, 24 hours urinary total protein; HbA1c, glycosylated hemoglobin, type A1c; TC, total cholesterol; Trig, triglyceride.

Supplementary Table 3: Detailed characterizations of nine Chinese patent medicines

eTable 4. Detailed characterizations of nine Chinese patent medicines.

CPMs	Source	SFDA approval number	Raw material	Quality control reported (Y/N)
Bailing capsule	Hangzhou zhongmei huadong Pharmaceutical Co., Ltd.	Z10910036	Fungus Powder of *Cordyceps sinensis* (Berk.) Sace. [clavicipitaceae] (Corayceps) (Cs-C-Q80).	Y*
Jinshuibao capsule	Jiangxi Jiminxin Jinshuibao Pharmaceutical Co., Ltd.	Z10890003	Fungus Powder of *Cordyceps sinensis* (Berk.) Sac.e [clavicipitaceae] (Corayceps) (Cs-4).	Y*
Huangkui capsule	Jiangsu Suzhong Pharmaceutical Co., Ltd	Z19990040	Abelmoschus *manihot* (L.) Medik. [malvaceae] (manihot).	Y*
Uremic clearance granule	Kangchen Pharmaceutical (Inner Mongolia) Co., Ltd	Z20072356	*Rheum palmatum* L. [polygonaceae] (Radix Rhei Et Rhizome); *Astragalus membranaceus* (Fisch.) Bunge. [leguminosae] (Hedysarum Multijugum Maxim.); *Morus alba* L. [Moraceae] (Mori Cortex); *Codonopsis pilosula* (Franch.) Nannf. [campanulaceae] (Codonopsis Radix); *Atractylodes macrocephala Koidz*. [compositae] (Atractylodes Macrocephala Koidz.); *Poria cocos* (Schw.) Wolf [polyporaceae] (Poria Cocos Wolf.); *Polygonum multiflorum Thunb*. [polygonaceae] (Fallopia multiflora Harald); *Paeonia lactiflora* Pall. [ranunculaceae] (ranunculaceae); *Salvia miltiorrhiza* Bge. [lamiaceae] (Radix Salviae); *Plantago asiatica* L. [plantaginaceae] (Plantaginis Herba); *Sophora flavescens* Ait. [leguminosae] (Sophorae Flavescentis Radix); *Pinellia ternata* (Thunb.) Breit. [araceae] (Arum Ternatum Thunb.); *Ligusticum chuanxiong* Hort. [umbelliferae] (Chuanxiong Rhizoma); *Bupleurum chinense* DC. [umbelliferae] (Radix Bupleuri); *Chrysanthemum morifolium* Ramat. [compositae] (Chrysanthemi Flos); *Glycyrrhiza uralensis* Fisch. [leguminosae] (licorice).	Y*
Compound xueshuantong capsule	Guangdong Zhongsheng Pharmaceutical Co., Ltd.	Z20030017	*Panaxnotoginseng* (Burk.) F.H.Chen. [araliaceae] (Panaxnotoginseng); *Astragalus membranaceus* (Fisch.) Bunge. [leguminosae] (Hedysarum Multijugum Maxim.); *Salvia miltiorrhiza* Bge. [lamiaceae] (Radix Salviae); *Scrophularia ningpoensis* Hemsl. [scrophulariaceae] (Figwort Root).	Y*

Shenyan kangfu tablet	Tianjin Tongrentang Group Co., Ltd	Z10940029/Z10940034	*Panax quiquefolium* L. [araliaceae] (Panacis Quinquefolii Radix); *Panax ginseng* C. A. Meyer [araliaceae] (Panax ginseng); *Rehmannia glutinosa* (Gaetn.) Libosch. [scrophulariaceae] (Figwort Root); *Eucommia ulmoides* Oliv. [eucommiaceae] (Eucommiae Cortex); *Dioscorea opposita* Thunb. [dioscoreaceae] (Rhizoma Dioscoreae); Oldenlandia diffusa (Will.) Roxb. [rubiaceae] (Hedyotis Diffusae Herba); Glycinemax(L.)merr. [leguminosae] (Sojae Semen Nigrum); *Smilax glabra* Roxb. [liliaceae] (Smilacis Glabrae Rhixoma); *Leonurus japonicus Houtt.* [lamiaceae] (Leonuri Herba); *Salvia miltiorrhiza* Bge. [lamiaceae] (Radix Salviae); *Alisma orientalis* (Sam.) Juzep. [alismataceae] (Alisma Orientale Juz.); *Imperata cylindrica* Beauv.var.major (Nees)C.E.Hubb. [graminaceous] (Imperatae Rhizoma); *Platycodon grandiflorum* (Jacq.) A.DC. [campanulaceae] (Platycodon Grandiforus).	Y*
Tripterygium glycosides	Shanghai Fudan Fuhua Pharmaceutical Co., Ltd	Z31020415	*Tripterygium wilfordii Hook.f.*[Celastraceae] (Tripterygii Radix).	Y*
Keluoxin Capsule	Chengdu Kanghong Pharmaceutical Co., Ltd	Z20090035	*Astragalus membranaceus* (Fisch.) Bunge. [leguminosae] (Hedysarum Multijugum Maxim); *Ligustrum lucidum* W.T.Aiton [Oleaceae] (Ligustrum lucidum Ait); *Hirudo nipponica Whitman* [Hirudinidae] (Whitmania pigra Whitman); *Rheum palmatum* L. [polygonaceae] (Radix Rhei Et Rhizome); *Pseudostellaria heterophylla* (Miq.) Pax [Caryophyllaceae] (pseudostellaria radix); *Lycium barbarum* L. [Solanaceae] (FRUCTUS LYCII).	Y*
Shenshuaining Tablet	Shanxi deyuantang Pharmaceutical Co., Ltd	Z20060030/Z20050503	*Pseudostellaria heterophylla* (Miq.) Pax [Caryophyllaceae] (Pseudostellaria radix); *Coptis chinensis Franch*. [Ranunculaceae] (Coptis chinensis adix et rhizome); *Pinellia ternata (Thunb.)* Makino [Araceae] (Pinellia ternate (Thunb.) Breit.); *Citrus × aurantium* L. [Rutaceae] (Dried ripe peel of Citrus reticulata Blanco); *Poria cocos (Schw.)* Wolf [Polyporaceae] (Poria Cocos Wolf.); *Rheum palmatum* L. [Polygonaceae] (Radix Rhei Et Rhizome); *Salvia miltiorrhiza Bunge* [Lamiaceae] (Salviae miltiorrhizae radix et rhizome); *Achyranthes bidentata Blume* [Amaranthaceae] (Achyranthes bidentata radix et rhizoma); *Carthamus tinctorius* L. [Asteraceae] (safflower Carthamus); *Glycyrrhiza uralensis* Fisch. ex DC. [Fabaceae] (Glycyrrhiza uralensis radix et rhizoma).	Y*

Notes: CPM, Chinese patent medicine; SFDA, State Food and Drug Administration; *, Prepared according to People's Republic of China Pharmacopoeia.

Supplementary Table 4

eTable 4. Basic Characteristics of the Included Studies.

Study ID	Male	Sample size	Mean age (y)	Disease duration (y)	Treatment duration(w)	Interventions	Outcomes
Chen J 2009	C:27<br>T:28	cah	C:58.5±8.0<br>T:59.5±7.8	C:11.5±4.0<br>T:11.6±3.9	C:12<br>T:12	C: Val 160mg, po, qd<br>T: BLC 1g, po, tid+Val160mg, po, qd	cah
Dai X 2017	C:18<br>T:19	cah	C:47.24±11.18<br>T:46.21±12.13	C:6.1±4.1<br>T:5.4±3.9	C:12<br>T:12	C: Val 80mg, po, qd<br>T: HKC2.5g, po, tid + Val80mg, po, qd	cah
Ding HH 2019	C:23<br>T:21	ceb	C:56.75±7.37<br>T:56.35±6.34	C:5~16<br>T:6~15	C:12<br>T:12	C: V al 80mg, po, qd<br>T: HKC 2.5g, po, tid +Val 80mg, po, qd	ceb
Ding T 2014	C+T:52	cd	T+C:51.2±9.5	NR	C:16<br>T:16	C: Irb 75mg, po, qd<br>T: JSB 0.99g, po, tid +Irb 75mg, po, qd	cd
Guan HB 2010	C:17<br>T:16	ce	C:52.2±6.5<br>T:53.3±5.7	C:9.9±2.9<br>T:10.9±3.4	C:24<br>T:24	C: Irb150mg, po, qd<br>T: BLC 1g, po, tid +Irb150mg, po, qd	ce
He XJ 2017	C:36<br>T:37	cbd	C:56.13±6.44<br>T:55.79±6.58	C:11.20±3.66<br>T:10.60±3.74	NR	C: Val 80mg, po, qd<br>T: BLC 2g, po, tid +Val 80mg, po, qd	cbd
Huang JY 2016	C:31<br>T:32	aebfg	C:58.21±5.18<br>T:58.21±6.02	C:8.32±1.43<br>T:8.08±1.13	C:12<br>T:12	C: Los 50mg, po, qd<br>T: BLC 2g, po, bid + Los 50mg, po, qd	aebfg
Jiang T 2021	C:38<br>T:41	cabdgh	C:56.87±5.81<br>T:57.74±5.32	C:9.46±2.13<br>T:9.63±2.48	C:8<br>T:8	C: Ram 2.5mg, po, qd<br>T: JSB 1.98g, po, tid + Ram 2.5mg, po, qd	cabdgh
Jin S 2018	C:27<br>T:30	cbfgh	C:55.4±5.9<br>T:58.4±5.9	C:8.3±3.1<br>T:8.1±3.2	C:8<br>T:8	C: Val 80mg, po, qd<br>T: HKC 2.5g, po, tid + Val 80mg, po, qd	cbfgh
Lei SH 2009	C+T:56	caeb	C+T:59.8±7.1	C+T:8.3±3.03	C:24<br>T:24	C: Irb 150mg, po, qd<br>T: JSB 1.98g, po, tid + Irb 150mg, po, qd	caeb
Li RN 2016	C:17<br>T:19	caeh	C:64.3±5.9<br>T:57.6±6.5	C:7.4±4.5<br>T:8.2±3.6	C:12<br>T:12	C: Ben 10mg, po, qd<br>T: UCG 5g, po, tid + Ben 10mg, po, qd	caeh
Li YT 2014	NR	cafgh	C+T:48.37±11.41	C+T:8.32±5.94	C:6<br>T:6	C: Val 80mg, po, qd<br>T: HKC 2.5g, po, tid +Val 80mg, po, qd	cafgh
Li Z 2019	C:37<br>T:35	ca	C:51~72<br>T:53~71	C:5~11<br>T:6~13	C:8<br>T:8	C: Irb 150mg, po, qd<br>T: BLC 2.5, po, tid + Irb 150mg, po, qd	ca
Liang F 2015	C:24<br>T:23	cafgh	C:43.98±12.03<br>T:45.68±11.97	NR	C:6<br>T:6	C: Val 80mg, po, qd<br>T: HKC 2.5g, po, tid + Val 80mg, po, qd	cafgh

Liu CP 2011	C:13<br>T:15	cah	C:52±14<br>T:54±17	C:5~20<br>T:5~22	C:12<br>T:12	C: Irb 150mg, po, qd<br>T: BLC 1g, po, tid + Irb 150mg, po, qd	cah
Liu JL 2015	C:19<br>T:17	c	C:61.3±7.9<br>T:60.1±8.1	NR	C:12<br>T:12	C: Irb 300mg, po, qd<br>T:TG 1mg/(kg·d), po, tid + Irb 300mg, po, qd	c
Liu WY 2017	C+T:43	ceb	C+T:63.3±5.7	NR	C:8<br>T:8	C: Val 80mg, po, qd<br>T: JSB 0.99g, po, tid + Val 80mg, po, qd	ceb
Luo F 2011	NR	c	NR	NR	C:12<br>T:12	C: Irb 150mg, po, qd<br>T: BLC 1g, po, tid + Irb 150mg, po, qd	c
Luo JG 2018	C:13<br>T:14	bd	C:54.75±10.69<br>T:53.61±12.86	C:4.56±1.74<br>T:3.58±1.14	C:12<br>T:12	C: Irb 150mg, po, qd<br>T: BLC 1g, po, tid+ Irb 150mg, po, qd	bd
Ma YL 2011	C:12<br>T:13	cae	C:46.2±7.9<br>T:48.1±9.3	C:6.8±1.5<br>T:6.5±1.2	C:12<br>T:12	C: Irb 150mg, po, qd<br>T: BLC 1, po, tid + Irb 150mg, po, qd	cae
Pan J 2016	C:24<br>T:28	cfg	C:65.7±5.2<br>T:64.5±4.7	C:11.6±6.3<br>T:12.3±5.9	C:12<br>T:12	C: Tel 80mg, po, qd<br>T: JSB 0.99, po, tid + Tel 80mg, po, qd	cfg
Qi MG 2016	C:23<br>T:20	d	C:60.3±11.2<br>T:61.4±10.7	C:8.78±5.01<br>T:8.46±5.23	C:12<br>T:12	C: Val 80mg, po, qd<br>T: HKC 2.5g, po, tid + Val 80mg, po, qd	d
Qiu FP 2016	C:12<br>T:10	ca	C:50.00±1.5<br>T:49.50±1.35	NR	C:12<br>T:12	C: Tel 40mg, po, qd<br>T: BLC 9g/d, po +Tel 40mg, po, qd	ca
Shen SM 2012	C+T:50	dh	C+T:65.2±15.8	C+T:5~15	C:12<br>T:12	C: Val 80mg/160mg, po, qd<br>T: BLC 1g, po, tid + Val 80mg/160mg, po, qd	dh
Shen XY 2018	C:17<br>T:18	cb	C:59.1±4.3<br>T:58.2±4.5	C:11.8±3.8<br>T:11.5±3.5	C:12<br>T:12	C: Irb 150mg, po, qd<br>T: JSB 0.99g, po, tid + Irb 150mg, po, qd	cb
Shi GC 2014	C:17<br>T:17	cafg	C:50.1±8.7<br>T:51.3±9.2	C:9.6±8.4<br>T:9.4±8,2	C:12<br>T:12	C: Per 4mg/d, po<br>T: UCG 5g, po, tid+10g, po, qn + Per 4mg/d, po	cafg
Tang W 2017	C:25<br>T:24	caedfgh	C:56.1±10.4<br>T:57.0±9.5	C:7.9±2.1<br>T:7.8±1.4	C:8<br>T:8	C: Val 80mg, po, qd<br>T: HKC 2.5g, po, tid + Val 80mg, po, qd	caedfgh
Wang FX 2009	C:18<br>T:20	cafg	C:58.12±8.28<br>T:57.45±9.82	NR	C:24<br>T:24	C: Tel 40mg, po, qd<br>T: UCG 5g, po, tid, qn + Tel 40mg, po, qd	cafg
Wang NN 2012	C:17<br>T:18	cedfgh	C:55.4<br>T:54.5	NR	C:24<br>T:24	C: Los P 50mg, po, qd<br>T: CXC 1.5g, po, tid + Los P 50mg, po, qd	cedfgh
Wang SY 2009	NR	ca	NR	NR	C:12<br>T:12	C: Ben 10mg, po, qd<br>T: BLC 2.5g, po, tid + Ben 10mg, po, qd	ca

Wang T 2019	C:24<br>T:20	ca	C:48.82±8.51<br>T:49.65±7.56	C:9.16±3.54<br>T:8.75±2.36	C:12<br>T:12	C: Val 150mg, po, qd<br>T: SYKFT 2.4g, po, tid + Val 150mg, po, qd	ca
Wang XC 2010	C:16<br>T:16	cad	C:59.46±15.31<br>T:58.89±15.98	NR	C:16<br>T:16	C: Val 80mg, po, qd<br>T: HKC 2.5g, po, tid + Val 80mg, po, qd	cad
Wang YH 2008	NR	ca	NR	NR	C:12<br>T:12	C: Tel 40mg, po, qd<br>T: BLC 2.5g, po, tid + Tel 40mg, po, qd	ca
Wei XF 2018	C:26<br>T:28	cebh	C:65.31±8.37<br>T:64.81±7.97	C:11.24±4.13<br>T:11.36±4.21	C:12<br>T:12	C: Olm 20mg, po, qd<br>T: UCG 5g, po, tid + Olm 20mg, po, qd	cebh
Wei SJ 2010	C+T:38	cd	C+T:51	C+T:10~18	C:12<br>T:12	C: Ben 10mg, po, qd<br>T: JSB 1.65g, po, tid + Ben 10mg, po, qd	cd
Wu P 2021	C:26<br>T:27	cah	C:59.84±3.67<br>T:59.87±3.69	C:8.36±1.57<br>T:8.37±1.58	C:4<br>T:4	C: Val 80mg, po, qd<br>T: UCG 5g, po, qid + Val 80mg, po, qd	cah
Wu QF 2016	C:22<br>T:18	cab	C:50.56±4.12<br>T:52.12±3.23	C:8.24±1.98<br>T:7.84±2.12	C:8<br>T:8	C: Can 4mg, po, qd<br>T: JSB 0.99g, po, tid + Can 4mg, po, qd	cab
Wu L 2014	C:16<br>T:17	cae	C:57.4±9.8<br>T:57.2±10.1	C:7.9±2.1<br>T:8.2±2.2	C:12<br>T:12	C: Val 160mg, po, qd<br>T: BLC 2g, po, tid + Val 160mg, po, qd	cae
Xiao ZZ 2010	C:17<br>T:17	cad	C:58.53±16.24<br>T:57.98±16.07	C+T:8.5±1.5	C:16<br>T:16	C: Val 80mg, po, qd<br>T: HKC 2.5g, po, tid + Val 80mg, po, qd	cad
Xu L 2015	C:29<br>T:30	a	C:52.1±4.9<br>T:50.9±5.1	NR	C:20<br>T:20	C: Irb mg, po, qd<br>T: JSB 0.99, po, tid + Irb 150mg, po, qd	a
Yang CH 2013	C:11<br>T:10	caefg	C:49.1±7.9<br>T:50.8±7.3	C:10.7±5.5<br>T:11.2±5.3	C:12<br>T:12	C: Los 50 mg, po, qd<br>T: JSB 0.99, po, tid + Los 50 mg, po, qd	caefg
Yang G 2016	C:23<br>T:21	cad	C:53.0±9.2<br>T:54.2±9.5	C:4.0±1.2<br>T:4.6±1.1	C:12<br>T:12	C: Irb 150mg, po, qd<br>T: BLC 1g, po, tid+ Irb 150mg, po, qd	cad
Yang WQ 2020	C:23<br>T:22	cab	C:52.5±7.2<br>T:53.3±6.8	C:7.1±1.6<br>T:7.3±1.7	C:12<br>T:12	C: Val 80mg, po, qd<br>T: HKC 2.5g, po, tid + Val 80mg, po, qd	cab
Ye FL 2016	C:57<br>T:65	cabfgh	C:33.05±4.01<br>T:33.25±4.35	C:6.10±3.71<br>T:6.90±3.68	C:12<br>T:12	C: Irb 300mg, po, qd<br>T: TG 1mg/(kg·d), po, tid + Irb 300mg, po, qd	cabfgh
Ye JB 2012	C:15<br>T:12	cad	C:54±5<br>T:55±5	NR	C:4<br>T:4	C: Can 8mg, po, qd<br>T: BLC 1g, po, tid + Can 8mg, po, qd	cad
Yu HT 2013	C+T:52	d	C+T:47.6±3.2	C+T:11.5±2.9	C:12<br>T:12	C: Olm 20mg, po, qd<br>T: JSB 0.99g, po, tid+ Olm 20mg, po, qd	d

Yun P 2013	C:28<br>T:30	aebfgh	C:55.1±7.2<br>T:53.5±6.4	C:6.1±2.7<br>T:6.6±3.1	C:12<br>T:12	C: Los 50mg, po, qd<br>T: CXC 1.5g, po, tid + Los 50mg, po, qd	aebfgh
Zhang C 2014	NR	ca	C+T:50.4±10.2	NR	C:18<br>T:18	C: Los P 50mg, po, qd<br>T: JSB 0.99g, po, tid + Los P 20mg, po, qd	ca
Zhou J 2012	C:19<br>T:18	cbh	C:43.89±13.06<br>T:45.76±12.23	C:7.52±0.87<br>T:8.14±0.93	C:12<br>T:12	C: Irb 300mg, po, qd<br>T: SYKFT 2.4g, po, tid + Irb 300mg, po, qd	cbh
Zhou XJ 2016	C:29<br>T:28	cafgh	C:66.3±2.3<br>T:65.9±2.4	C:6.8±0.4<br>T:6.9±0.5	C:8<br>T:8	C: Val 80mg, po, qd<br>T: HKC 2.5g, po, tid + Val 80mg, po, qd	cafgh
Zhu HY 2015	C:27<br>T:30	caf	C:57.5±6.9<br>T:57.4±6.4	C:10.5±4.8<br>T:10.0±5.1	C:16<br>T:16	C: Los 50mg, po, qd<br>T: BLC 2.5g, po, tid + Los 50mg, po, qd	caf
Cai JY 2010	C:18<br>T:19	cafg	C:52±5<br>T:NR	NR	C:12<br>T:12	C: Olm 20mg, po, qd<br>T: UCG 5g, po, qid + Olm 20mg, po, qd	cafg
Cai XY 2010	C:13<br>T:12	cafgh	C:44.89±12.75<br>T:45.66±12.23	NR	C:8<br>T:8	C: Val 80mg, po, qd<br>T: HKC 2.5g, po, tid+ Val 80mg, po, qd	cafgh
Cao XC 2015	C:30<br>T:31	cagh	C:57.4±6.1<br>T:57.8±6.0	C:10.6±4.4<br>T:10.3±4.9	C:16<br>T:16	C: Val 80mg, po, qd<br>T: BLC 1.0g, po, tid +Val 80mg, po, qd	cagh
Cao XX 2017	C:13<br>T:15	a	C:58.93±11.24<br>T:59.43±14.14	C:8.86±4.98<br>T:8.36±5.28	C:24<br>T:24	C: Val 80mg, po, qd<br>T: JSB 1.98g, po, tid + Val 80mg, po, qd	a
Cao YX 2019	C:21<br>T:23	cab	C:57.2±7.2<br>T:56.3±6.8	C:6.4±1.9<br>T:6.2±1.8	C:12<br>T:12	C: Ben 5mg, po, qd<br>T: JSB 0.99g, po, tid + Ben 5mg, po, qd	cab
Chen F 2010	C+T:26	ad	C+T:36~73	C+T:8.42±3.56	C:8<br>T:8	C: Val 80mg, po, bid<br>T: BLC 1.0g, po, tid + Val 80mg, po, bid	ad
Chen QS 2016	C:19<br>T:17	cabh	C:63.4±5.0<br>T:62.3±4.7	NR	C:12<br>T:12	C: Ben 10mg, po, qd<br>T: BLC 1.0g, po, tid + Ben 10mg, po, qd	cabh
Chen QJ 2017	C+T:62	cb	C+T:66.5±2.7	C+T:10.3±0.8	NR	C: Los P 50mg, po, qd<br>T: JSB 0.99g, po, tid + Los P 50mg, po, qd	cb
Chen SS 2020	C:17<br>T:16	cabdh	C:53.68±6.09<br>T:53.80±6.17	C:5.95±1.71<br>T:5.83±1.63	C:8<br>T:8	C: Ben H10mg, po, qd<br>T: KLX 2.0g, po, tid + Ben H 10mg, po, qd	cabdh
Chen SW 2018	C:23<br>T:22	cb	C:63.81±7.60<br>T:66.23±7.41	C:12.80±3.92<br>T:15.13±3.11	C:12<br>T:12	C: Los P 100mg, po, qd<br>T: SSNT 1.8g, po, tid + Los P 100mg,po,qd	cb
Chen Y 2020	C:18<br>T:20	bh	C:66.38±4.72<br>T:66.29±4.83	C:8.65±2.73<br>T:8.24±2.86	C:2<br>T:2	C: Los P 50mg, po, qd<br>T: SSNT 2.1g, po, tid + Los P 50mg, po, qd	bh

Chen Y 2015	C+T:36	caeh	C+T:56.31±4.16	C+T:10.12±2.16	C:12<br>T:12	C: Irb 75mg, po, qd + Ena 5mg, po, qd<br>T: HKC 2.5g, po, tid + Irb 75mg, po, qd + Ena 5mg, po, qd	caeh
Chi WP 2012	C+T:72	a	C+T:51	C+T:10~18	C:8<br>T:8	C: Ben 10mg, po, qd<br>T: JSB 1.65g, po, tid + Ben 10mg, po, qd	a
Dai XM 2012	C:23<br>T:25	abfg	C:52.2±1.3<br>T:51.7±5.2	C:13.1±5.9<br>T:12.4±6.1	C:12<br>T:12	C: Ben H 10mg, po, qd<br>T: CXC 1.5g, po, tid + Ben H 10mg, po, qd	abfg
Deng SY 2014	C:14<br>T:16	cafgh	C:45.3±12.4<br>T:42.3±11.8	C:9.9±4.6<br>T:11.3±5.1	C:16<br>T:16	C: Val 80mg, po, qd<br>T: HKC 2.5g, po, tid + Val 80mg, po, qd	cafgh
Deng SY 2016	C:26<br>T:25	cb	C:54.7±5.2<br>T:53.4±4.8	NR	C:12<br>T:12	C: Cap 25mg, po, tid<br>T: SSNT 1.75g, po, tid + Cap 25mg, po, tid	cb
Dou JF 2006	NR	cab	C:51.03±11.14<br>T:52.08±8.26	C:5.83±2.90<br>T:5.78±2.54	C:12<br>T:12	C: Val 80mg, po, qd<br>T: HKC 2.0g, po, tid + Val 80mg, po, qd	cab
Fan YT 2015	C+T:47	b	C+T:58.66±2.17	NR	C:8<br>T:8	C: Can 4mg, po, qd<br>T: JSB 0.99g, po, tid + Can 4mg, po, qd	b
Feng ZL 2017	C:24<br>T:23	ebdh	C:47.02±2.06<br>T:46.92±2.11	C:6.02±0.93<br>T:6.34±0.89	C:4<br>T:4	C: Val 80mg, po, qd<br>T: JSB 0.99g, po, tid + Val 80mg, po, qd	ebdh
Gao X 2018	C:50<br>T:49	ce	C:67.19±5.21<br>T:67.20±5.34	C:11.18±3.40<br>T:11.21±3.52	C:6<br>T:6	C: Los P 50mg, po, qd<br>T: JSB 0.99g, po, tid + Los P 50mg, po, qd	ce
Ge QR 2011	C+T:65	abdh	C+T:57.4	NR	C:8<br>T:8	C: Ben H 40mg, po, qd<br>T: JSB 1.65g, po, tid + Ben H 20mg, po, qd	abdh
Gu RY 2018	C+T:36	cah	C+T:45.29±3.62	NR	C:8<br>T:8	C: Val 80mg, po, qd<br>T: HKC 2.5g, po, tid + Val 80mg, po, qd	cah
Guan YH 2020	C:28<br>T:27	b	C:41.65±4.95<br>T:41.98±4.26	C:7.98±1.12<br>T:8.06±1.05	C:8<br>T:8	C: Irb 300mg, po, qd<br>T: SYKFT 2.88g, po, tid + Irb 300mg, po, qd	b
Guan CA 2021	C:26<br>T:28	cabdh	C:52.29±9.06<br>T:51.91±7.72	C:9.11±3.72<br>T:9.27±3.52	C:2<br>T:2	C: Can 4~8mg, po, qd<br>T: BLC 2.0g, po, tid + Can 4~8mg, po, qd	cabdh
Guo G 2015	C:35<br>T:37	cdfgh	C:42.5±11.5<br>T:43.1±10.9	NR	C:8<br>T:8	C: Val 80mg, po, qd<br>T: HKC 2.5g, po, tid + Val 80mg, po, qd	cdfgh
Guo T 2016	C:16<br>T:16	a	C:61.8±6.1<br>T:61.1±6.5	C:14.5±2.2<br>T:14.3±2.3	C:12<br>T:12	C: Ben H 10mg, po, qd<br>T: JSB 0.99g, po, tid + Ben H10mg, po, qd	a
He P 2012	C:24<br>T:28	caedh	C:57.4±8.9<br>T:56.6±8.4	C:10.6±6.9<br>T:10.2±6.3	C:12<br>T:12	C: Val 80mg, po, qd<br>T: JSB 0.99g, po, tid+ Val 80mg, po, qd	caedh

He XH 2012	NR	ca	NR	NR	C:8<br>T:8	C: Can 8mg, po, qd<br>T: KLX 2.0g, po, tid + Can 8mg, po, qd	ca
He YY 2021	C:24<br>T:23	cabdh	C:61.5±8.3<br>T:62.2±8.5	C:6.4±1.2<br>T:6.5±1.4	C:12<br>T:12	C: Val 80mg, po, qd<br>T: UCG 5g, po, qid + Val 80mg, po, qd	cabdh
He YN 2010	C:22<br>T:21	cbh	C:43.3±13.6<br>T:45.5±15.2	C:7.8±4.8<br>T:8.0±5.2	C:12<br>T:12	C: Val 80mg, po, qd<br>T: HKC 2.5g, po, tid + Val 80mg, po, qd	cbh
Hu QS 2007	NR	cbfgh	NR	NR	C:12<br>T:12	C: Val 80mg, po, qd<br>T: BLC 2.0g, po, tid + Val 80mg, po, qd	cbfgh
Hu WF 2012	C:21<br>T:22	aed	C:46.96<br>T:61.98	C:10<br>T:8.5	C:16<br>T:16	C: Irb 150mg, po, qd<br>T: BLC 2.0g,po,tid+Irb 150mg,po,qd	aed
Hu XJ 2019	C:29<br>T:32	cabd	C:55.36±5.28<br>T:55.64±5.42	C:7.56±2.56<br>T:7.26±2.71	C:12<br>T:12	C: Ben 5mg, po, qd<br>T: JSB 0.99g, po, tid + Ben 5mg, po, qd	cabd
Hu YG 2016	C:11<br>T:13	cbd	C:56.3±5.6<br>T:57.5±5.7	C:5.5±1.4<br>T:5.7±1.6	C:8<br>T:8	C: Val 80mg, po, qd<br>T: HKC 2.5g, po, tid + Val 80mg, po, qd	cbd
Hu Y 2016	C+T:87	cbd	C+T:48.7	C+T:9.3	C:12<br>T:12	C: Irb 150mg, po, qd<br>T: BLC 0.4g, po, tid + Irb 150mg,po,qd	cbd
Huang T 2010	C:24<br>T:25	aed	C:71.3±11.6<br>T:71.1±10.7	C:10.9±5.7<br>T:10.4±5.2	C:12<br>T:12	C: Per 4mg, po, qd<br>T: JSB 1.98g, po, tid + Per 4mg, po, qd	aed
Jia ZW 2015	C:18<br>T:27	cedh	C:52.53±14.61<br>T:50.30±15.50	C:6.6±3.8<br>T:6.0±5.6	C:4<br>T:4	C: Can 4mg, po, qd<br>T: HKC 2.0g, po, tid + Can 4mg, po, qd	cedh
Xie WY 2019	C:24<br>T:23	cbd	C:63.51±4.25<br>T:64.53±4.39	C:10.52±2.03<br>T:11.02±2.14	C:12<br>T:12	C: Irb 150~300mg, po, qd<br>T: JSB 0.99g, po, tid + Irb 150~300mg, po, qd	cbd
Jin XB 2016	C:28<br>T:26	cbdfg	C:54.1±10.1<br>T:55.3±9.8	C:11.5±4.3<br>T:11.9±4.6	C:12<br>T:12	C: Val 80mg, po, qd<br>T: BLC 4.0g, po,t id+ Val 80mg, po, qd	cbdfg
Lan YL 2021	C:26<br>T:28	cbh	C:60.15±4.02<br>T:60.08±4.17	C:8.18±1.09<br>T:8.25±1.06	C:12<br>T:12	C: Los P 50mg, po, qd<br>T: UCG 5.0g, po, qid + Los P 50mg, po, qd	cbh
Li HJ 2013	C:16<br>T:18	b	C:39~65<br>T:40~75	NR	C:12<br>T:12	C: Irb 150mg, po, qd<br>T: BLC 5.0g, po, tid + Irb 150mg, po, qd	b
Li BY 2015	NR	c	C:52.50±20.80<br>T:52.00±20.60	NR	C:12<br>T:12	C: Tel 80mg, po, qd<br>T: SSNT 5.0g, po, tid + Tel 80mg, po, qd	c
Li HS 2017	C:32<br>T:33	bh	C:60.2±4.5<br>T:61.5±5.0	C:12.2±4.5<br>T:12.2±5.0	C:8<br>T:8	C: Los P 50mg, po, qd<br>T: HKC 2.5g, po, tid + Los P 50mg, po, qd	bh

Li HN 2022	C:22<br>T:23	cebd	C:56.77±10.32<br>T:57.13±10.44	C:9.64±2.73<br>T:9.78±2.64	C:12<br>T:12	C: Ben 10mg, po, qd/Val 80mg, po, qd<br>T: BLC1.0g, po, tid + Ben 10mg, po, qd/Val 80mg, po, qd	cebd
Li Q 2020	C:23<br>T:24	d	C:65.81±3.49<br>T:65.92±3.57	C:5.36±1.28<br>T:5.40±1.37	NR	C: Ben H 10mg, po, qd<br>T: BLC 1.0g, po, tid + Ben H 10mg, po, qd	d
Li Q 2021	C:27<br>T:24	cbh	C:64.55±11.24<br>T:63.24±11.36	C:10.13±3.75<br>T:9.47±3.21	C:12<br>T:12	C: Irb 150mg, po, qd<br>T: UCG 5g, po, tid,10g, po, qn + Irb 150mg, po, qd	cbh
Li QH 2010	C:19<br>T:22	abh	C:53±7.6<br>T:52±8.1	C:3.2±2.0<br>T:3.1±2.2	C:8<br>T:8	C: Val 80mg, po, qd<br>T: HKC 2.5g, po, tid + Val 80mg, po, qd	abh
Li YN 2013	C:24<br>T:21	cah	C:49.5<br>T:51	C:9.5<br>T:9	C:12<br>T:12	C: Irb 150mg, po, qd<br>T: JSB 0.99g, po, tid + Irb 150mg, po, qd	cah
Liang YP 2014	C+T:27	fgh	C+T:43.2±12.8	NR	C:8<br>T:8	C: Val 80mg, po, qd<br>T: HKC 2.5g, po, tid + Val 80mg, po, qd	fgh
Lin M 2013	NR	c	C+T:45~87	C+T:5~26	C:24<br>T:24	C: Irb 150~300mg, po, qd<br>T: SYKFT 2.4g, po, tid + Irb 150~300mg, po, qd	c
Lin M 2011	C:15<br>T:14	cafgh	C:46.85±4.95<br>T:47.42±5.23	NR	C:8<br>T:8	C: Irb 150mg, po, qd<br>T: HKC 2.5g, po, tid + Irb 150mg, po, qd	cafgh
Lin ZN 2016	C:20<br>T:20	cbh	NR	NR	C:12<br>T:12	C: Ben 10mg, po, qd<br>T: BLC 1.0g, po, tid + Ben 10mg, po, qd	cbh
Liu CP2 2011	C:13<br>T:15	cdh	C:52±14<br>T:54±17	C:5~20<br>T:5~22	C:12<br>T:12	C: Irb 150mg, po, qd<br>T: BLC 1.0g, po, tid +I rb 150mg, po, qd	cdh
Liu L 2021	C:20<br>T:20	b	C:65.11±5.22<br>T:65.22±5.36	C:10.24±2.14<br>T:10.31±2.22	C:12<br>T:12	C: Olm 20mg, po, qd<br>T: UCG 5.0g, po, tid + Olm 20mg, po, qd	b
Liu L 2012	C:12<br>T:11	ca	C:55.1±9.1<br>T:53.5±8.7	NR	C:12<br>T:12	C: Ena 10mg, po, qd<br>T: KLC 2.0g, po, tid+Ena10mg, po, qd	ca
Liu XQ 2012	C:20<br>T:18	ca	C:56.45±9.86<br>T:56.35±9.82	NR	C:24<br>T:24	C: Irb 150mg, po, qd<br>T: UCG 5.0g, po, tid, qn + Irb 150mg, po, qd	ca
Liu XD 2011	C:46<br>T:48	cabd	C:56.8±5.1<br>T:57.7±5.3	C:6.5±0.6<br>T:6.4±0.9	C:8<br>T:8	C: Ben 10mg, po, qd<br>T: BLC 3.0g, po, tid + Ben 10mg, po, qd	cabd
Liu YP 2018	C+T:54	cabf	C+T:56.3±13.9	NR	C:8<br>T:8	C: Tel 40mg, po, qd<br>T: HKC 2.0g, po, tid + Tel 40mg, po, qd	cabf
Liu YH 2011	C:20<br>T:20	cad	C:48.6±5.27<br>T:49.7±5.83	C:5.1±0.38<br>T:5.04±0.32	C:12<br>T:12	C: Irb 150mg, po, qd<br>T: BLC 1.0g, po, tid + Irb 150mg, po, qd	cad

Lou PH 2010	C:15<br>T:16	ch	C:60±8<br>T:61±10	C:9±2.8<br>T:10±2.6	C:12<br>T:12	C: Val 80mg, po, qd<br>T: BLC 1g, po, tid + Val 80mg, po, qd	ch
Luo JJ 2021	C:56<br>T:61	cbh	C:68.05±2.14<br>T:70.12±3.26	C:9.12±2.14<br>T:9.53±1.69	C:12<br>T:12	C: Los 25mg, po, qd<br>T: BLC 2.0g, po, tid + Los 25mg, po, qd	cbh
Lv F 2012	C+T:32	cah	NR	NR	C:12<br>T:12	C: Ena 10mg, po, bid<br>T: JSB 0.99g, po, tid + Ena 10mg, po, bid	cah
Lv MF 2015	C:23<br>T:24	abfgh	C:56.9±2.7<br>T:57.3±3.1	C:7.9±0.2<br>T:7.8±0.3	C:8<br>T:8	C: Val 80mg, po, qd<br>T: HKC 2.5g, po, tid + Val 80mg, po, qd	abfgh
Nie XY 2018	C:33<br>T:35	cbfg	C:55.9±3.9<br>T:56.6±3.3	C:7.9±1.8<br>T:7.1±1.5	C:3<br>T:3	C: Val 80mg, po, qd<br>T: TG 0.3~0.5mg/kg, po, tid + Val 80mg, po, qd	cbfg
Ou YL 2015	C:15<br>T:14	ceh	C:53.6±5.2<br>T:54.3±4.8	C:3.5±1.2<br>T:3.4±1.5	C:12<br>T:12	C: Ena M 5mg, po, qd + Val 75mg, po, qd<br>T: HKC 2.5g, po, tid+ Ena M 5mg, po, qd +Val75mg,po,qd	ceh
Pan CY 2016	C:25<br>T:23	cb	C:52.6±4.3<br>T:54.5±4.2	C:5.1±3.3<br>T:5.3±4.1	C:12<br>T:12	C: Irb 150mg, po, qd<br>T: SSNT 1.4~2.1g, po, tid + Irb 150mg, po, qd	cb
Qi JY 2018	NR	cebfg	NR	NR	C:8<br>T:8	C: Irb 150mg, po, qd<br>T: JSB 1.65g, po, tid + Irb 150mg, po, qd	cebfg
Qiao AM 2013	C:33<br>T:32	ah	C:54.6±12.4<br>T:55.9±11.8	C:11.5±3.7<br>T:11.9±3.9	C:12<br>T:12	C: Tel 80mg, po, qd<br>T: BLC 1.0g, po, tid + Tel 80mg, po, qd	ah
Ren X 2020	C:66<br>T:65	bh	C:62.6±5.5<br>T:62.8±5.4	C:2.2±0.6<br>T:2.3±0.4	C:12<br>T:12	C: Irb 150mg, po, qd<br>T: BLC 1.0g, po, tid + Irb 150mg, po, qd	bh
Shen ML 2015	C:15<br>T:14	ae	C:51.5±6.5<br>T:52.5±6.9	C:3.4±2.7<br>T:3.5±2.8	C:24<br>T:24	C: Can 4mg, po, qd<br>T: JSB 1.98g, po, tid + Can 4mg, po, qd	ae
Su JF 2019	C:12<br>T:13	aebfgh	C:55.36±10.45<br>T:55.41±10.56	C:10.52±2.36<br>T:10.38±3.41	C:8<br>T:8	C: Ben H 10mg, po, qd<br>T: CXC1.05g, po, tid + Ben H 10mg, po, qd	aebfgh
Sun FY 2021	C:23<br>T:22	cebd	C:61.23±8.72<br>T:60.98±8.17	C:6.63±0.61<br>T:6.57±0.64	C:12<br>T:12	C: Irb 150mg, po, qd<br>T: JSB 0.99g, po, tid + Irb 150mg, po, qd	cebd
Sun JY 2015	C:20<br>T:21	ca	C:32.85±5.8<br>T:33.85±5.9	C:3.2±0.5<br>T:3.3±0.6	C:8<br>T:8	C: Los P 50mg, po, qd<br>T: JSB 0.99g, po, tid + Los P 50mg, po, qd	ca
Sun SR 2014	C:59<br>T:60	b	C:59<br>T:59.5	C:1~5<br>T:0.8~5	C:12<br>T:12	C: Irb 150mg, po, qd<br>T: SYKFT 1.92g, po, tid + Irb 150mg, po, qd	b
Tang YZ 2011	C:24<br>T:23	caeb	C:57.7±4.2<br>T:59.2±3.8	C:17±2.8<br>T:16±3.6	C:12<br>T:12	C: Tel 40mg, po, qd<br>T: JSB 0.99g, po, tid + Tel 40mg, po, qd	caeb

Tian XY 2019	C:28<br>T:30	cbdh	C:66.48±6.52<br>T:67.12±6.47	C:11.08±4.82<br>T:10.98±4.65	C:12<br>T:12	C: Val 80mg, po, qd<br>T: BLC 4.0g, po, tid + Val 80mg, po, qd	cbdh
Wang AY 2020	C:21<br>T:20	cefg	C:64.51±5.69<br>T:64.89±5.82	C:7.21±2.50<br>T:7.48±2.37	C:8<br>T:8	C: Can 4mg, po, qd<br>T: JSB 0.99g, po, tid + Can 4mg, po, qd	cefg
Wang G 2006	C:18<br>T:20	abfg	C:45.1±13.3<br>T:43.2±12.1	C:5.69±2.01<br>T:5.92±2.09	C:8<br>T:8	C: Ben H 10mg, po, qd<br>T: JSB 0.99g, po, tid + Ben H 10mg, po, qd	abfg
Wang JM 2015	C+T:28	cd	C+T:54.56±12.46	C+T:5.28±3.67	C:12<br>T:12	C: Los 50mg, po, qd<br>T: BLC 2.0g, po, bid + Los 50mg, po, qd	cd
Wang KY 2020	C:18<br>T:17	aebh	C:49.6±5.9<br>T:49.5±5.8	C:7.45±2.71<br>T:7.33±2.63	C:8<br>T:8	C: Val 80mg, po, qd<br>T: BLC 1.5g, po, tid + Val 80mg, po, qd	aebh
Wang LW 2018	C:24<br>T:23	bfg	C:55.4±10.1<br>T:54.8±11.0	C:4.6±2.0<br>T:4.5±2.2	C:12<br>T:12	C: Can 4~8mg, po, qd<br>T: JSB 0.99g, po, tid + Can 4~8mg, po, qd	bfg
Wang XH 2015	C:37<br>T:35	dh	C:54±8.92<br>T:57±6.46	C:7.6±3.28<br>T:7.2±2.93	C:14<br>T:14	C: Val 80~160mg, po, bid<br>T: BLC 1.5g, po, tid + Val 80~160mg, po, bid	dh
Wu N 2013	C:34<br>T:35	eg	C:58.7±10.2<br>T:56.3±11.5	C:10.4±4.6<br>T:10.6±5.2	C:24<br>T:24	C: Val 80mg, po, bid<br>T: CXC 1.5g, po, tid + Val 80mg, po, bid	eg
Xiao XY 2011	C:22<br>T:21	cah	C:43~70<br>T:42~71	C:6.8~14.9<br>T:6.5~15.3	C:12<br>T:12	C: Val 80mg, po, bid<br>T: UCG 5g, po, qid + Val 80mg, po, bid	cah
Xiao Y 2021	C:35<br>T:37	cabh	C:64.31±4.92<br>T:63.87±4.98	C:9.82±1.94<br>T:9.21±1.81	NR	C: Irb 150mg, po, qd<br>T: BLC 1.0g, po, tid + Irb 150mg, po, qd	cabh
Xu JZ 2016	C:15<br>T:13	ca	C:50.1±4.3<br>T:51.5±5.7	C:7.1±1.9<br>T:6.4±1.6	C:24<br>T:24	C: Irb 150mg, po, qd<br>T: UCG 5g, po, qid + Irb 150mg, po, qd	ca
Xu WM 2013	C+T:32	ch	C+T:36±14.5	C+T:7.5±4.5	C:4<br>T:4	C: Irb 150mg, po, qd<br>T: HKC 2.5g, po, qid + Irb 150mg, po, qd	ch
Xu XM 2020	C:16<br>T:17	caefgh	C:52.5±12.6<br>T:53.2±12.7	C:8.6±1.5<br>T:8.4±1.1	C:8<br>T:8	C: Val 80mg, po, qd<br>T: HKC 2.5g, po, tid +Val 80mg, po, qd	caefgh
Yang P 2014	C:12<br>T:13	caeg	C:53.6±4.9<br>T:52.1±5.3	C:13.2±5.0<br>T:12.8±4.8	C:12<br>T:12	C: Ben H 10mg, po, qd<br>T: CXC1.5g, po, tid + Ben H 10mg, po, qd	caeg
Yang T 2013	C:17<br>T:18	abh	C:35.2±3.7<br>T:34.6±3.6	C:5.0~11<br>T:6.0~10	C:24<br>T:24	C: Val 80mg, po, qd<br>T: TG 1~1.5mg/(kg·d) + Val 80mg, po, qd	abh
Yang XM 2012	C:18<br>T:16	abh	C:56±7<br>T:57±7	C:6.1±2.0<br>T:5.5±2.2	C:8<br>T:8	C: Ena 10mg, po, qd<br>T: JSB 0.99g, po, tid + Ena 10mg, po, qd	abh

Yu HJ 2022	C:18<br>T:19	cad	C:63.53±6.36<br>T:64.04±6.46	C:6.32±1.78<br>T:6.76±1.57	C:8<br>T:8	C: Can 4mg, po, qd<br>T: JSB 0.99g, po, tid + Can 4mg, po, qd	cad
Yu JW 2021	C:28<br>T:29	c	C:61.73±6.58<br>T:60.85±4.62	C:5.98±1.25<br>T:6.02±1.22	C:8<br>T:8	C: Val 80mg, po, qd<br>T: KLX 2.0g, po, tid + Val 80mg, po, qd	c
Yuan JF 2019	C:30<br>T:32	caebh	C:56.89±3.65<br>T:58.01±3.70	C:12.03±3.21<br>T:12.42±3.31	C:8<br>T:8	C: Val 80mg, po, qd<br>T: SSNT 5.0g, po, tid + Val 80mg, po, qd	caebh
Yuan YH 2017	C:26<br>T:28	cafg	C:53.3±2.6<br>T:53.9±2.4	C:7.3±3.3<br>T:7.6±2.9	C:12<br>T:12	C: Tel 40mg, po, qd<br>T: BLC 3.0g, po, tid + Tel 40mg, po, qd	cafg
Zhang LF 2010	C:20<br>T:22	b	C:50.2±10.5<br>T:54.2±8.4	C:0.2~10.8<br>T:0.3~10.3	C:12<br>T:12	C: Can 4mg, po, qd<br>T: JSB 0.99g, po, tid + Can 4mg, po, qd	b
Zhang M 2021	C:19<br>T:18	b	C:54.17±3.45<br>T:54.21±3.42	NR	C:8<br>T:8	C: Val 80mg, po, qd<br>T: SSNT 5.0g, po, tid + Val 80mg, po, qd	b
Zhang Q 2020	C:16<br>T:15	bdh	C:57.48±4.36<br>T:56.71±4.59	NR	C:16<br>T:16	C: Val 80mg, po, qd<br>T: BLC 1.0g, po, tid + Val 80mg, po, qd	bdh
Zhang RX 2016	C:21<br>T:21	bh	C:50.86±7.97<br>T:51.74±8.16	NR	C:8<br>T:8	C: Val 80mg, po, qd<br>T: HKC 2.5g, po, tid + Val 80mg, po, qd	bh
Zhang XD 2016	C:40<br>T:39	abdfgh	C:47.3±5.4<br>T:47.7±5.2	C:6.3±2.4<br>T:6.2±2.1	C:24<br>T:24	C: Irb 150mg, po, qd<br>T: KLX 2.0g, po, tid + Irb 150mg, po, qd	abdfgh
Zhang Y 2021	C:46<br>T:50	cab	C:50.85±4.65<br>T:50.90±4.71	C:12.35±2.98<br>T:12.48±3.05	C:12<br>T:12	C: Irb 150mg, po, qd<br>T: JSB 0.99g, po,t id + Irb 150mg, po, qd	cab
Zhang YY 2019	C:18<br>T:16	cabh	C:50.34±6.57<br>T:51.17±6.96	C:5.74±3.82<br>T:5.56±2.69	C:8<br>T:8	C: Irb 150mg, po, qd<br>T: JSB 1.98g, po, tid+ Irb 150mg, po, qd	cabh
Zhang Z 2019	C+T:60	e	C:59.2±9.7<br>T:58.9±10.1	C:8.45±1.60<br>T:8.49±1.61	C:12<br>T:12	C: Val 80mg, po, bid<br>T: CXC 1.5g, po, tid + Val 80mg, po, bid	e
Zhang ZY 2014	NR	afg	NR	NR	C:8<br>T:8	C: Can 4mg, po, qd<br>T: JSB 0.99g, po, tid + Can 4mg, po, qd	afg
Zhang LP 2021	C:17<br>T:16	cab	C:43.21±3.64<br>T:42.67±3.18	NR	C:8<br>T:8	C: Ben H 10mg, po, qd<br>T: KLX 2.0g, po, tid + Ben H 10mg, po, qd	cab
Zheng JQ 2011	C:19<br>T:15	ca	C:45~71<br>T:47~63	NR	C:12<br>T:12	C: Irb 150mg, po, qd<br>T: BLC 1.0g, po, tid+Irb 150mg, po, qd	ca
Zhou GJ 2013	C+T:58	cdh	C:58.20±7.16<br>T:57.48±6.78	NR	C:8<br>T:8	C: Irb 150mg, po, qd<br>T: KLX 2.0g, po, tid + Irb 150mg, po, qd	cdh

Zhou SQ 2015	C:30<br>T:32	cabdg	C:57±5<br>T:57±5	C:6.3±2.7<br>T:6.0±3.5	C:12<br>T:12	C: Irb 150mg, po, qd<br>T: CXC 1.5g, po, tid + Irb 150mg, po, qd	cabdg
Zhu HY 2020	C:21<br>T:23	h	C:62.11±3.15<br>T:62.15±3.18	NR	C:12<br>T:12	C: Val 80mg, po, qd<br>T: HKC 2.5g, po, tid + Val 80mg, po, qd	h

Abbreviations: mo, month; y, years; w, weeks; T, treatment group; C, control group; ACEI, angiotensin-converting enzyme inhibitors, ARB, angiotensin receptor blockers; BLC, Bailing Capsule; JSB, Jinshuibao Capsule; HKC, Huangkui Capsule; UCG, Uremic Clearance Granule; TG, Tripterygium glycosides; CXC, Compound Xueshuantong Capsule; SYKFT, Shenyan Kangfu Tablet; KLX, Keluoxin Capsule; SSNT, Shenshuaining Table; Val, Valsartan; Irb, Irbesartan; Los, Losartan; Tel, Telmisartan; Los P, Losartan potassium; Olm, Olmesartan; Can, Candesartan; Ram, Ramipril; Ben, Benazepril; Per, Perindopril; Ben H, Benazepril hydrochloride; Ena, Enalapril; Cap, Captopril; Ena M, Enalapril maleate; a, Urinary albumin excretion rate; b, Overall response rate; c, Serum creatinine; d, 24 hours urinary total protein; e, Glycosylated Hemoglobin, Type A1c; f, Total cholesterol; g, Triglyceride; h, Adverse effects; NR, not reported; Data are presented as means ± standard deviation.

Supplementary Table 5: Cost-effective ranking of nine Chinese patent medicines

eTable 6. Cost-effective ranking of nine Chinese patent medicines.

Drugs	Specifications	Dosages	Durations (mo)	Quantities (box)	Unit-price (RMB)	Total price (RMB)	Cost-effective rank
BLC	0.5g×48	2.0g, tid	3	22.5	48	1080	5
JSB	0.33g×63	1.98g, tid	3	25.7	40	1028	4
HKC	0.5g×30	2.5g, tid	3	45	32	1440	9
UCG	5g×15	5g, tid	3	18	60	1080	5
TG	10mg×50	1mg/kg/d	3	10.8	10	108	1
CXC	0.5g×60	1.5g, tid	3	13.5	48	648	3
SYKFT	0.48g×45	2.4g, tid	3	30	20	600	2
KLX	0.5g×60	2.0g, tid	3	18	75	1350	7
SSNT	0.35g×40	1.75g, tid	3	33.8	40	1352	8

BLC, Bailing Capsule; JSB, Jinshuibao; HKC, Huangkui Capsule; UCG, Uremic Clearance Granule; TG, Tripterygium glycosides; CXC, Compound Xueshuantong Capsule; SYKFT, Shenyan Kangfu Tablet; KLX, Keluoxin Capsule; SSNT, Shenshuaining Tablet; mo, month; RMB, Renminbi.
